# Mild dyslipidemia accelerates tumorigenesis through expansion of Ly6C^hi^ monocytes and differentiation to pro-angiogenic myeloid cells

**DOI:** 10.1038/s41467-022-33034-0

**Published:** 2022-09-14

**Authors:** Thi Tran, Jean-Remi Lavillegrand, Cedric Lereverend, Bruno Esposito, Lucille Cartier, Melanie Montabord, Jaouen Tran-Rajau, Marc Diedisheim, Nadège Gruel, Khadija Ouguerram, Lea Paolini, Olivia Lenoir, Emmanuel Pinteaux, Eva Brabencova, Corinne Tanchot, Pauline Urquia, Jacqueline Lehmann-Che, Richard Le Naour, Yacine Merrouche, Christian Stockmann, Ziad Mallat, Alain Tedgui, Hafid Ait-Oufella, Eric Tartour, Stephane Potteaux

**Affiliations:** 1 Université Paris Cité, Inserm, PARCC, F-75015 Paris, France; 2 Université de Reims Champagne Ardenne, IRMAIC EA 7509, 51097 Reims, France; 3 Département de Recherche, Institut Godinot, 51100 Reims, France; 4 Service de diabétologie, Hôpital Cochin APHP. GlandOmics, Cheverny, Paris, France; 5 INSERM U830, Équipe Labellisée LNCC, Diversity and Plasticity of Childhood Tumors Lab, PSL Research University, Institut Curie Research Centre, Institut Curie, 75005 Paris, France; 6 Department of Translational Research, Institut Curie Research Centre, Institut Curie, 75005 Paris, France; 7 Université de Nantes, INRAE, UMR 1280 PhAN, Nantes, France; 8 Faculty of Biology, Medicine and Health, University of Manchester, Manchester, UK; 9 Université Paris Cité, INSERM, U976 HIPI, F-75010 Paris, France; 10 Molecular Oncology Unit, Saint Louis Hospital, APHP, F-75010 Paris, France; 11 Division of Cardiovascular Medicine, Department of Medicine, University of Cambridge, Cambridge, UK; 12 Equipe Labellisée Ligue Contre le Cancer, Paris, France; 13 AP-HP Hôpital Européen Georges Pompidou. Service d’immunologie, Paris, France; 14 Present Address: University of Zurich, Institute of Anatomy, Zurich, Switzerland; 15 Present Address: Comprehensive Cancer Center Zurich, Zurich, Switzerland; 16 Present Address: Université Paris Cité, INSERM, U976 HIPI, F-75010 Paris, France

**Keywords:** Cancer microenvironment, Dyslipidaemias

## Abstract

Cancer and cardiovascular disease (CVD) share common risk factors such as dyslipidemia, obesity and inflammation. However, the role of pro-atherogenic environment and its associated low-grade inflammation in tumor progression remains underexplored. Here we show that feeding C57BL/6J mice with a non-obesogenic high fat high cholesterol diet (HFHCD) for two weeks to induce mild dyslipidemia, increases the pool of circulating Ly6C^hi^ monocytes available for initial melanoma development, in an IL-1β-dependent manner. Descendants of circulating myeloid cells, which accumulate in the tumor microenvironment of mice under HFHCD, heighten pro-angiogenic and immunosuppressive activities locally. Limiting myeloid cell accumulation or targeting VEGF-A production by myeloid cells decrease HFHCD-induced tumor growth acceleration. Reverting the HFHCD to a chow diet at the time of tumor implantation protects against tumor growth. Together, these data shed light on cross-disease communication between cardiovascular pathologies and cancer.

## Abstract (web summary)

Obesity and inflammation have been associated to cancer progression. Here, the authors show that high fat and cholesterol diet, in a non-obese context, promotes tumourigenesis through increasing inflammatory monocytes and myeloid-derived pro-angiogenic factors.

## Introduction

Metabolic syndrome (MS) is an established risk factor for cancer and cardiovascular disease (CVD), the two leading causes of morbidity and mortality worldwide. Components of the MS include excess body fat, increased blood pressure, high blood sugar, and dyslipidemia^1^. The metabolically unhealthy phenotype is defined by two or more of four of these components. In several types of cancers, dyslipidemia and obesity are associated with increased death rate^2,3^. This has also been functionally validated in experimental models of diet-induced obesity, which showed accelerated tumor growth^4,5^. Even though obesity is often associated with metabolic disorders, 24–30% of normal-weight adults are considered metabolically unhealthy, according to several worldwide meta-analyses^6,7^. These metabolically unhealthy normal weight individuals are commonly dyslipidemic. They present high circulating levels of cytokines and cardiometabolic risk factors comparable to those observed in metabolically unhealthy obese individuals^8^. A recent study has proposed chronic systemic low-grade inflammation, observed in non-obese patients with stable CVD, as a contributing factor to cancer incidence^9^. Dysfunctional fatty acid and cholesterol metabolism promotes tumor growth directly through activation of oncogenic signaling pathways and formation of lipid rafts^10,11^, and indirectly through increased production and mobilization of monocytes^12,13^. Generally, high numbers/percentages of circulating monocytes indicate poor clinical prognosis in cancer^14,15^, suggesting that monocytes in blood may influence tumor development. In melanoma patients, enrichment of circulating monocytes/myeloid-derived suppressor cells (MDSCs) is associated with higher risk of disease progression^16^. MDSCs are a heterogeneous group of immature myeloid cells and are the main subset of leukocytes infiltrating the tumors. Two types of MDSC have been identified: in mice, polymorphonuclear MDSC (PMN-MDSC) and monocytic MDSC (M-MDSC) that are phenotypically related to neutrophils (CD11b^+^Ly6C^lo^Ly6G^+^) and monocytes (CD11b^+^Ly6C^hi^Ly6G^–^), respectively. In the tumor, M-MDSC can differentiate into tumor-associated macrophages (TAM) (CD11b^+^ Ly6C^lo^ F4/80^+^ MHCII^+^)^17^. In tumor-bearing host, myeloid cells are recruited to tumor sites by chemokines produced by the tumor, such as CCL2 and CCL5^18,19^. In chronic tumor-associated inflammation, the presence of cytokines, such as IL‐1β, mediate MDSC expansion^20^. TAMs and MDSCs contribute to tumor progression by suppressing T-cell functions and promoting tumor angiogenesis, proliferation, survival, and metastasis^21,22^. VEGF-A is produced by many cell types, including tumor cells and myeloid cells. As a major inducer of blood vessel growth, VEGF-A is involved in tissue remodeling in the context of cancer and cardiovascular diseases. In endothelial cells, VEGF-A and IL-1β share common signaling pathways and may synergistically regulate the expression of inflammatory genes and growth factors^23^. Anti-angiogenic agents and immunotherapies targeting VEGF-A are thought to create a transient window of vessel normalization, which might facilitate the diffusion of therapeutic agents^24,25^. Anti-IL-1β therapies, initially developed in the context of autoimmune diseases, have recently gained a great interest in the cardiovascular and oncology fields^26^. The major randomized trial on the role of IL-1β inhibition in atherosclerosis, CANTOS, reported a significant reduction in the number of incident cases of lung cancer in patients treated with anti-IL-1β antibody compared to placebo-treated patients^27^. This clinical study provided strong evidence that IL-1β antagonism could prevent both the recurrence of cardiovascular events and tumor development in patients with a persistent pro-inflammatory response.

To extend these previous results, we decided to evaluate the impact of mild-dyslipidemia-associated inflammation on solid tumor growth in mice. To address this issue, we employed a high fat/high cholesterol atherogenic diet (HFHCD). We found that low-grade systemic inflammation induced by the HFHCD differed from inflammation generated by classic high-fat diet (HFD) used in mouse models of obesity. Two-week feeding with HFHCD accelerated solid B16 melanoma development in C57BL/6 J mice and induced an angiogenic and inflammatory signature in the tumor. Acceleration of tumor growth was associated with increased CCR2^+^ Ly-6C^hi^ monocyte levels in blood and early recruitment into the tumors. IL-1β deficiency inhibited blood monocytosis and reduced tumor expansion under HFHCD. Other strategies used to interfere with myeloid cell accumulation also reduced the expansion of tumors under HFHCD. In the tumor microenvironment, MDSCs significantly contributed to the increase of VEGF-A, but not of IL-1β, under HFHCD and exerted increased immunosuppressive activities. Specific loss of myeloid-derived VEGF-A recapitulated the protective effect of myeloid cell depletion in mice under HFHCD. Reverting the HFHCD to a chow diet at the time of tumor implantation slowed down tumor growth to the basal level, thus showing that the pro-tumoral consequence of HFHCD regimen is reversible. This study demonstrates that silent low-grade inflammation fuels early-growing solid tumors with a pro-tumoral contingent of myeloid cells, and highlights the crucial and successive roles of IL-1β and VEGF-A in this process.

## Results

### Short term high fat/high cholesterol diet sets up a low-grade inflammation that differs from an obesogenic high fat diet in C57BL/6J mice

**Fig. 1:**
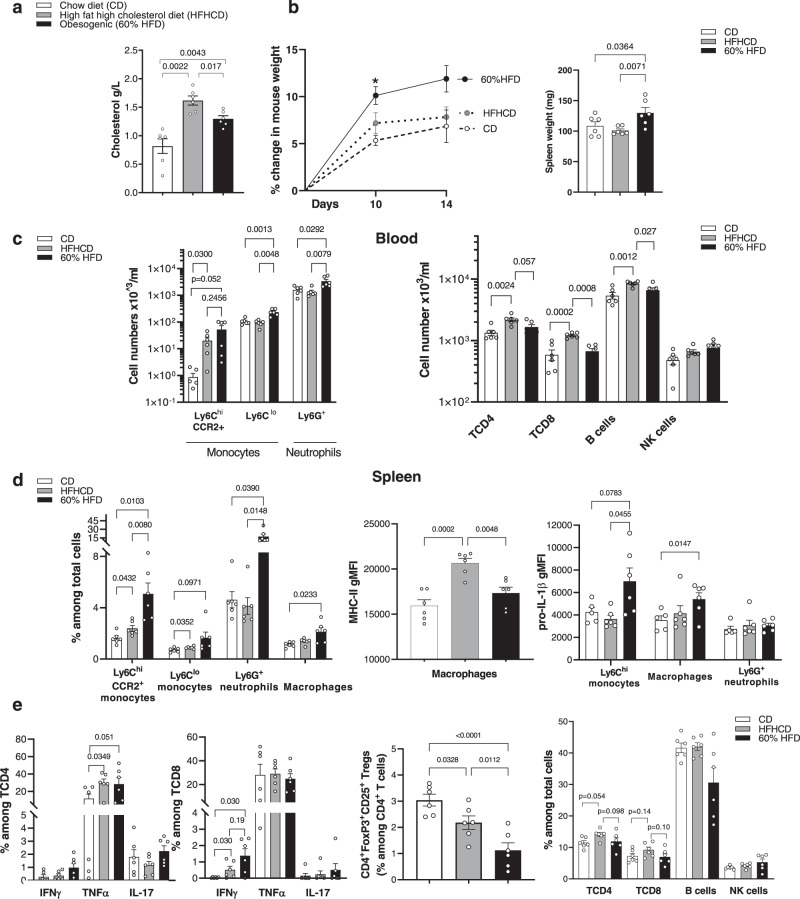
Atherogenic HFHCD induces a specific low-grade inflammation. C57BL/6J mice were fed for 2 weeks with chow diet (CD), high fat high cholesterol diet (HFHCD) or obesogenic diet (60% HFD). **a** Plasmatic cholesterol measured by colorimetric assay. **b** Mouse weight variation (left) and spleen weight (right). **c**–**e** Flow cytometry analysis **c** in blood **d**, **e** in spleen. Splenocytes were incubated with Golgistop™1X/GolgiPlug ™ for 4 h in complete medium before flow analysis of % IFNγ, TNFα, IL-17 in CD4^+^ and CD8^+^ T cells. (gMFI: geo Mean fluorescence intensity). Data are expressed as mean ± s.e.m. *n* = 6 mice/group, one-way ANNOVA with Tukey’s multiple comparison test. Source data are provided as a Source Data file.

To establish the originality of a model of silent dyslipidemia in a non-obese setting, we performed a comprehensive analysis of the inflammatory state of mice under HFHCD (15% FAT, 1.25% cholesterol), in comparison to mice fed with an obesogenic HFD (60% FAT) (Supplemental Table 1). After 2 weeks, cholesterol levels were higher in mice under HFHCD (Fig. 1a). On the other hand, mouse weight gain and spleen weight were significantly increased in mice under 60% HFD in comparison to mice under HFHCD (Fig. 1b). In contrast to the 60% HFD group, where all myeloid subsets were increased in blood, only CCR2^+^ Ly6C^hi^ monocytes were expanded in the HFHCD group (Fig. 1c and Supplementary Fig. 1a). On the other hand, CD8^+^ T cells and B cells were only increased in the HFHCD group (Fig. 1c). In the spleen, HFHCD led to an increase in CCR2^+^Ly6C^hi^ and Ly6C^lo^ monocytes but to a lesser extent than in the 60% HFD group (Fig. 1d and Supplementary Fig. 1b). Splenic macrophages from the HFHCD group displayed a mild M1-like pro-inflammatory phenotype defined by higher level of MHC-II but lower level of pro-IL-1β, as compared to macrophages from the 60% HFD group (Fig. 1d). HFHCD and 60% HFD consumption led to comparable elevated production of TNFα by CD4^+^ T cells and IFNγ by CD8^+^ T cells and decreased levels of regulatory T cells, without change in other T cell populations (Fig. 1e). These data show that the consumption of a HFHCD for 2 weeks leads to a specific low-grade inflammation, which has never been tested in tumorigenesis before.

### HFHCD accelerates melanoma tumor development in C57BL/6J mice

**Fig. 2:**
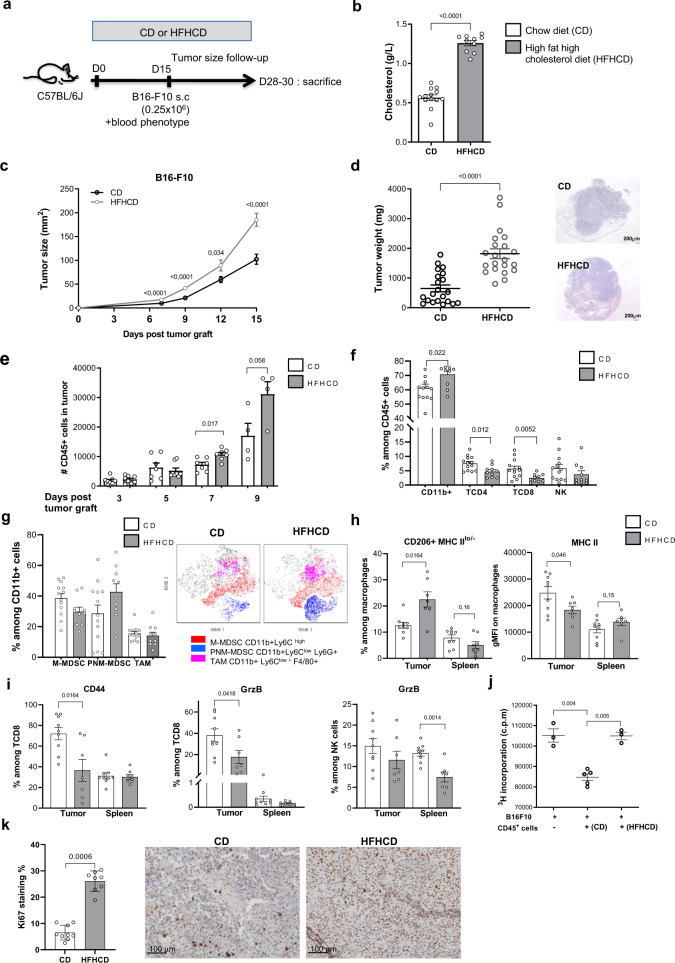
Pro-atherogenic HFHCD accelerates B16-F10 melanoma growth in C57BL/6J mice. **a** Experimental design. **b** Total blood cholesterol (*n* = 13 in CD group, *n* = 10 in HFHCD group, pool of two independent experiments). **c** Tumor growth evolution at day 7 (*n* = 23 in CD group, *n* = 17 in HFHCD group), day 9 (*n* = 35 in CD group, *n* = 24 in HFHCD group), day 12 (*n* = 22 in CD group, *n* = 13 in HFHCD group) and day 15 (*n* = 26 in CD group, *n* = 19 in HFHCD group). Data were pooled from five experiments. **d** tumor weight at the time of sacrifice (*n* = 21 mice/group, pool of five independent experiments). Representative microphotographies of tumor sections after hematoxilin/eosin coloration. **e** Quantification of CD45^+^ leukocytes in the tumor, by flow cytometry (*n* = 8 mice/group for evaluation at day 3, 5, 7 and *n* = 8/group at day 9. Data were pooled from three experiments). Flow cytometry analysis of **f** main subsets of leukocytes (%) in tumors at the time of sacrifice (*n* = 13 in CD group and *n* = 10 in HFHCD group), **g** and of M-MDSC (CD11b^+^ Ly6C^hi^), PNM-MDSC (CD11b^+^ Ly6C^lo^ Ly6G^+^) and TAM (CD11b^+^Ly6C^lo^ Ly6G^-^ F4/80^+^) among CD11b^+^ cells in the tumor (*n* = 13 in CD group and *n* = 10 in HFHCD group, pool of two independent experiments) (left), and t-SNE plots representing an overlay of total CD45^+^ cells (gray) and M-MDSC (red), PNM-MDSC (blue) and TAM (purple) (right). **h** gMFI of MHC II and (%) CD206^+^MHC II^lo^ in macrophages (M2 markers) in tumor and spleen (*n* = 9 in CD group and *n* = 7 in HFHCD group, unpaired *t*-test). **i** Expression of CD44 (%) and granzyme B (%) among CD8^+^ and NK cells in the tumor and spleen (*n* = 9 in CD group and *n* = 7 in HFHCD group). **j** B16-F10 proliferation was measured by 3H-Thymidine incorporation. B16-F10 were cultured, for 24 h, alone (*n* = 3) or with CD45^+^ cells isolated from CD (*n* = 5) or HFHCD tumors (*n* = 3). **k** Ki-67 immunohistochemistry staining was performed on FFPE tumor. (left) Quantification of % DAB staining and (right) representative picture of Ki-67 staining in the CD group (*n* = 9) and in the HFHCD group (*n* = 8). Scale bar = 100 µm. Data are expressed as mean ± s.e.m. Analysis of difference within groups were performed with Mann–Whitney *t*-test, and with one-way ANOVA with Bonferroni’s multiple adjustment test. Source data are provided as a Source Data file.

We aimed to investigate the impact of the HFHCD on tumorigenesis in vivo. After 2 weeks of diet, we subcutaneously injected B16-F10 melanoma cells to mice and followed tumor growth (Fig. 2a). Mice were sacrificed up to 15 days post tumor cell injection, when tumor size reached the ethic endpoint limit. Within this timeframe, we did not observe any metastasis in the lung nor in the liver. Increased blood cholesterol levels in mice remained different between the HFHCD and the CD group at the end of the experiment (Fig. 2b). We found that HFHCD increased tumor size and weight (Fig. 2c, d), in comparison with the CD group. Similar results were obtained, although to a lesser extent, when mice were grafted with TC1 tumor cell, a more immunogenic cell line expressing human papillomavirus type 16 protein E6/E7 (Supplementary Fig. 2a). Therefore, we chose to focus on the melanoma model for the rest of the study.

We studied the early kinetics of leukocyte accumulation in the growing tumors. Using flow cytometry, we found a progressive accumulation of CD45^+^ leukocytes within the tumors over time (Fig. 2e). Nevertheless, leukocytes accumulated more extensively as early as 7 days in tumors of HFHCD group, suggesting a temporal link between immune cell infiltration and tumor expansion.

Next, we characterized the accumulation of leukocyte subtypes at the day of sacrifice. CD11b^+^ myeloid cells dominated the CD45^+^ infiltrates in both groups but frequency was higher in HFHCD tumors (Fig. 2f). In contrast, T cell and NK cell proportions were decreased in the tumors of HFHCD group in comparison to CD group. Among CD11b^+^ cells, percentages of CD11b^+^Ly6C^hi^ (M-MDSC), CD11b^+^Ly6C^lo^Ly6G^+^ (PNM-MDSC) cells and F4/80^+^ tumor-associated macrophages (TAM) were not significantly different between the two groups (Fig. 2g and Supplementary Fig. 2b). Nevertheless, CD11b^+^ Ly6C^hi^ (M-MDSCs) of the HFHCD group were enriched in CCR2^+^ cells with lower expression of CD11b (Supplementary Fig. 2c). Tumor macrophages of HFHCD fed mice displayed a M2-like phenotype characterized by higher percentage of CD206^+^MHC-II^lo^ cells and lower MHC-II expression (Fig. 2h). Local activation and cytotoxicity marker expression of recruited CD8^+^ T cells and NK cells was diminished in the HFHCD group as confirmed by the detection of membrane marker CD44 and Granzyme B (Fig. 2i). No difference was observed in PD-1 and PDL-1 expression on infiltrating myeloid cells and CD8^+^ T cells between the 2 diets (Supplementary Fig. 2d). Interestingly, the phenotype of splenic macrophages did not differ between groups (Fig. 2h, i), suggesting that the tumor microenvironment shapes the recruited leukocytes in situ, promoting immunosuppressive activities while dampening anti-tumoral functions. In line with that, we found that CD45^+^ immune cells extracted from HFHCD-derived tumors were less effective than their counterparts isolated from CD tumors, in inhibiting tumor cell growth ex vivo (Fig. 2j).

To investigate whether the HFHCD affected the metabolism of either immune cells infiltrating the tumor or cancer cells, we first used the fluorescent D-glucose derivative (2-NBDG) probe to monitor glucose uptake in vivo. Glucose was preferentially consumed by myeloid cells. We found that incorporation of 2-NBDG by non-immune cells (CD45^−^) and immune cells (CD45^+^) was similar in tumors from mice fed CD or HFHCD (Supplementary Fig. 3a). Concordant results were obtained with FDG-PET-Scan (fluorodeoxyglucose positron emission tomography), where mean FDG uptake intensity was similar in tumors of HFHCD-fed mice compared to tumors of CD-fed mice (Supplementary Fig. 3b–d). Thus, glucose combustion differs between cellular subsets in vivo in tumors but the pattern is not modulated by the diet. Then we used fluorescently labeled palmitate (C16 BODIPY) to assess lipid uptake in vivo. Palmitate was taken up by both immune and non-immune (containing tumor cells) cells, with no significant difference between CD and HFHCD tumors (Supplementary Fig. 3e). To evaluate if the HFHCD could impact tumor lipogenesis, as increase in fatty acid production can enhance tumor cell proliferation and survival^28^, we compared fatty acid composition in tumors by gas chromatography–flame ionization detection (GC–FID). We found a significant increase in proportion of mono-unsaturated fatty acids (MUFAs) and a higher omega-6/omega-3 ratio in the HFHCD-derived tumors (Supplementary Fig. 3f and through https://osf.io/te8s2/). The increased proportion of MUFAs suggests that lipogenesis is increased in HFHCD fed-mice tumors compared to CD. High omega-6/omega-3 ratio is known to promote the pathogenesis of many diseases such as cardiovascular diseases, cancers and inflammatory diseases^29^. Accordingly, we observed that the addition of an excess of theses fatty acids in vitro enhanced B16-F10 proliferation (Supplementary Fig. 3g). These data indicate that the HFHCD does not affect the metabolism of cells in the tumor microenvironment, but rather promotes the accumulation of MDSC and modulates cell lipogenesis, which probably additionally support tumor cell survival and proliferation. Adding to evidence, B16-F10 cells proliferated more when cultured in medium supplemented with tumor explant supernatant (TES) from HFHCD compared to TES from CD-derived tumors (Supplementary Fig. 3h), and higher Ki67 proliferation index in situ was observed in tumors under HFHCD (Fig. 2k).

### Circulating Ly6C^hi^ monocytes accumulate more in tumors under HFHCD

**Fig. 3:**
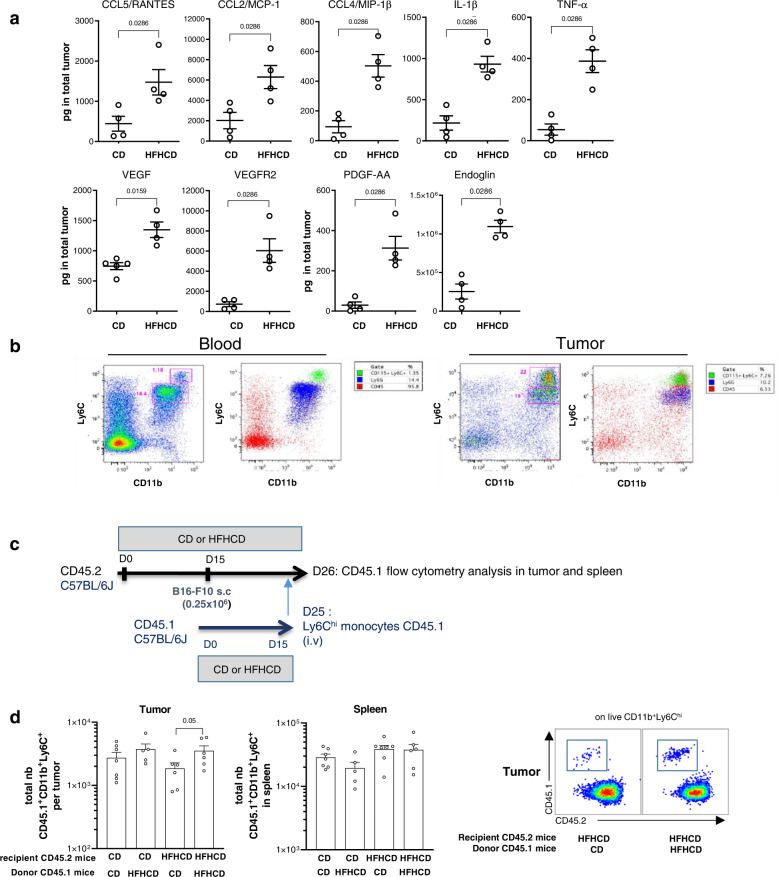
HFHCD favors migration of myeloid cells into the tumor. C57BL/6J mice were fed for 2 weeks with chow diet (CD) or high fat high cholesterol diet (HFHCD), then grafted with B16-F10 melanoma cells. **a** Cytokines and chemokines were measured by bead-based multiplex immunoassay (*n* = 4 mice/group) or Elisa (*n* = 5 in CD group and *n* = 4 in HFHCD group) in total B16-F10 tumor in CD or HFHCD mice at day 15 after tumor graft. **b** In vivo labeling of monocytes and neutrophils at day 8 after tumor graft: anti-Ly6G and anti-CD115 antibody were injected i.v. Six hours after injection, labeled cells were identified in blood and tumors by flow cytometry. Representive dot plots were showed. **c**, **d** Adoptive transfer of Ly6C^hi^ monocytes from CD45.1 donor mice fed with CD or HFHCD into CD45.2 recipient mice bearing B16-F10 tumor fed with CD or HFHCD. **c** Experimental design. **d** Quantification of CD45.1 Ly6C^hi^ monocytes in tumors and spleen 18 h after monocyte transplantation into CD45.2 recipient mice. Representative flow cytometry large dot plots illustrating gating of CD45.2 and CD45.1 among CD11b + Ly6C^hi^ cells in tumors HFHCD. Data are expressed as mean±sem. Analysis of difference within groups were performed with two-sided Mann–Whitney *t*-test. Source data are provided as a Source Data file.

As we showed that HFHCD significantly increased monocyte numbers in blood and in tumors, we asked how the diet modulated monocyte production and whether it may change the migratory behavior of circulating monocytes. First, we found that HFHCD increased hematopoietic progenitor cell (HSPCs) amount and retention in the bone marrow (Supplementary Fig. 4). This could be explained by an increase of growth factors, with leptin being significantly augmented in the medullar niche, and by the induction of *CXCR4* expression in progenitor cells (Supplementary Fig. 4a–c). Besides, SDF-1/CXCL12 chemokine gradient appeared to be altered by HFHCD, which was paralleled by a decreased expression of CXCR4 on mature myeloid cells in the bone marrow, in favor of increased monocyte egress to the blood compartment (Supplementary Fig. 4d, e). The expression level of *CCR2* in Ly6C^hi^ monocytes was similar in both groups (Supplementary Fig. 4f). Second, analyses performed on tumor lysates revealed that chemokines involved in myeloid cell migration (CCL5, CCL2, CCL4), together with inflammatory (IL-1β and TNFα) and pro-angiogenic factors (VEGF-A, VEGFR2, PDGF, Endoglin) were increased in tumors of mice fed a HFHCD (Fig. 3a). We validated in our model that myeloid cells in the tumor derived, at least in part, from circulating blood cells. In vivo labeling of circulating monocytes and neutrophils was performed with intravenous injection of a combination of labeled anti-CD115 plus anti-Ly6G in mice. After 6 h, we found labeled monocytes in the CD11b^+^Ly6C^hi^ population, and labeled neutrophils in the CD11b^+^Ly6C^int^ population (Fig. 3b).

To investigate whether HFHCD increased the recruitment of Ly6C^hi^ monocytes to the tumor, we performed adoptive transfer of Ly6C^hi^ monocytes from bone marrow of CD45.1 mice into congenic CD45.2 recipient tumor-bearing mice (Fig. 3c). The recruitment of Ly6C^hi^ monocytes isolated from HFHCD-fed mice was slightly, but not significantly increased in tumors of CD-fed mice, as compared with monocytes isolated from CD-fed mice (Fig. 3d). When HFHCD-fed tumor-bearing mice were used as recipients (at a time-point where their tumors were of comparable size to tumors of CD-fed mice), the recruitment of CD45.1 monocytes from a HFHCD environment was significantly increased as compared with monocytes from CD mice. In contrast, no statistical differences were observed in the spleen (Fig. 3d). Altogether, these data show that priming of both Ly6C^hi^ monocytes and tumors are involved in the increased accumulation of tumor myeloid cells under HFHCD.

### Blocking myeloid cell accumulation in the tumor limits tumor growth under HFHCD

**Fig. 4:**
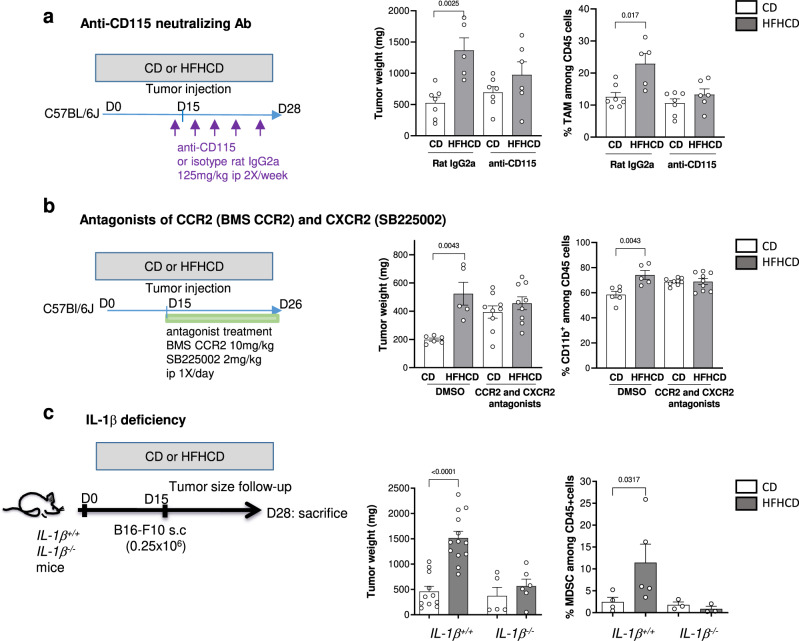
Impaired myeloid cell accumulation in the tumor limits tumor growth under HFHCD. C57BL/6J mice, fed with CD or HFHCD and grafted with B16-F10 cells. **a** Neutralizing anti-CD115 (CSF1R) or isotype (125 mg/kg, ip) was injected one day before tumor graft then every 3 days. (left) Experimental design, (middle) tumor weight and (right) flow cytometry analysis of TAM in the tumor (*n* = 14 mice on CD, *n* = 11 mice on HFHCD, among which *n* = 7 and *n* = 6 mice were respectively treated with anti-CD115 antibody). **b** CCR2 antagonist (BMS CCR2, 10 mg/kg, ip) and CXCR2 antagonist (SB225002, 2 mg/kg) were injected every day from the day of tumor graft. (Left) Experimental design, (middle) Tumor weight and (right) flow cytometry analysis of CD11b^+^ myeloid cells in the tumor. (*n* = 15 mice on CD, *n* = 14 mice on HFHCD, among which *n* = 9 mice per group were treated with the agonist combination). **c** *IL-1β*^*−/−*^ mice (*n* = 11) and control *IL-1β*^*+/+*^ mice (*n* = 23) were fed for 2 weeks with CD (*n* = 5 and *n* = 10 respectively in each group) or HFHCD (*n* = 6 and *n* = 13 respectively in each group), grafted with B16-F10 cells and sacrificed at day 13 post injection. (Left) Tumor weight and (right) flow cytometry analysis of total MDSC (%) in the tumor (*n* = 4 *IL-1β*^*-/-*^ mice on CD, *n* = 5 *IL-1β*^*−/−*^ mice on HFHCD, *n* = 4 *IL-1β*^*+/+*^ mice on CD, *n* = 5 *IL-1β*^*+/+*^ mice on HFHCD). Data are expressed as mean ± s.e.m. Analysis of difference within groups were performed with two-sided Mann–Whitney *t*-test. Source data are provided as a Source Data file.

We next tested if the increased availability and recruitment of Ly6C^hi^ monocytes was required for HFHCD-accelerated tumor growth. First, we treated mice with a neutralizing anti-CD115 antibody, which has been shown to alter the differentiation of tumor-associated macrophages due to impaired CSF1/CSF1R signaling, without modifying monocytes in the circulation^30^. We found that blocking CD115 signaling slightly altered tumor progression in tumors of mice under HFHCD and reduced the accumulation of tumor macrophages in this group (Fig. 4a). Second, we pharmacologically blocked myeloid cell migration into the tumor using a combination of CCR2 (BMS CCR2) and CXCR2 (SB225002) chemokine antagonists. Upon treatment, the aggravating effect of HFHCD on tumor size was abrogated, as was the HFHCD-dependent accumulation of myeloid cells into the tumors (Fig. 4b). We next tested whether limiting blood monocyte number could recapitulate those findings. In dyslipidemic mice, IL-1β has been established as a central regulator of myelopoiesis^31^. Although the quantity of IL-1β was under the level of detection in the plasma of mice from the two groups, the increased production of IL-1β found in tumors under HFHCD led us to address the role of IL-1β in promoting circulating Ly6C^hi^ monocyte expansion in our model. *IL-1β*-deficient mice and control mice were subjected to HFHCD or CD. Under steady state, IL-1β deficiency was characterized by a decreased level of blood neutrophils (Supplementary Fig. 5a) and was associated with partial limitation of tumor growth (Fig. 4c), as previously reported^32^. Under HFHCD, apart from maintaining differences in neutrophils, IL-1β deficiency completely inhibited the expansion of blood monocytes and protected mice from tumor growth acceleration (Fig. 4c and Supplementary Fig. 5a). IL-1β deficiency also impaired MDSC accumulation into the tumors of mice under HFHCD (Fig. 4c), but did not impair MDSC function as they retained a high to even greater immunosuppressive activity against proliferative OT-1 T cells (Supplementary Fig. 5b). In the tumor microenvironment, flow cytometry analysis showed that among immune cells, pro-IL-1β was mainly expressed by myeloid cells as no expression was detected in T cells or in CD45^-^ cells (Supplementary Fig. 5c). We confirmed the lack of IL-1β expression in B16-F10 cell line by qPCR. Because monocyte-derived cells can increase their IL-1β production in mouse models of atherosclerosis^33^, we addressed the possible role of myeloid cell-derived IL-1β in developing tumors under HFHCD. Quite surprisingly, we found that the expression levels of IL-1β in MDSC isolated from tumors of CD and HFHCD-fed mice were similar, despite high immunosuppressive activities in HFHCD group (Supplementary Fig. 5d). We tested the role of myeloid cell-derived IL-1β in tumors by using mice with specific conditional alteration of IL-1β signaling in myeloid cells. These mice were generated by crossing *IL-1R1*^*fl/fl*^ ^34^ to *LysMCre* mice. When put on HFHCD, *IL-1R1*^*ΔLysM*^ mice increased tumor size, despite impaired IL-1β signaling and decreased pro-IL-1β expression (Supplementary Fig. 5e). These data show that IL-1β signaling and production in tumor myeloid cells is not responsible for tumor expansion in response to a HFHCD. Together, these data show that priming of both Ly6C^hi^ monocytes and the tumor add to the surplus of monocyte to maximize monocyte recruitment into the tumor in condition of HFHCD.

### Under HFHCD, infiltrating myeloid cells control tumor growth via VEGF-A production

**Fig. 5:**
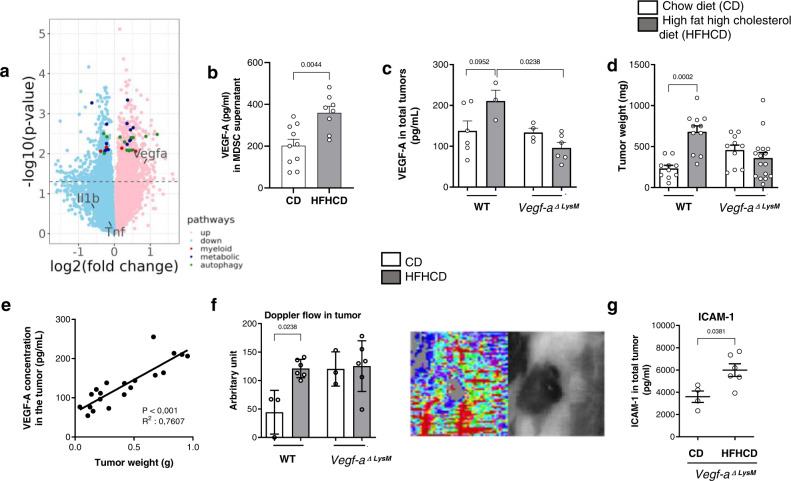
Myeloid-derived VEGF-A controls tumor growth under HFHCD. C57BL/6J mice, fed with CD or HFHCD and grafted with B16-F10 cells. **a** Volcano plot of all expressed genes, with *Vegfa* and differentially expressed genes from significantly enriched pathway highlighted. **b** MDSCs were isolated from B16-F10 tumor at day 13 after tumor graft, and cultured for 18 h in complete medium. VEGF-A was measured by bead-based multiplex immunoassay in the supernatant (*n* = 10 mice in CD group, *n* = 8 mice in HFHCD group). **c**–**e** *LysMCre*^*+/−*^/*Vegf-a*^*f/f*^ (*Vegf-a *^*ΔLysM*^) and their control littermate *LysMCre*^*−/−*^*/Vegf-a*^*f/f*^ (WT) mice were fed for 2 weeks with CD or HFHCD, and were transplanted with B16-F10 cells (s.c.). Mice were sacrificed at day 15 post injection. **c** VEGF-A production in total tumors was measured by ELISA (*n* = 6 in WT mice on CD, *n* = 3 in WT mice on HFHCD, *n* = 4 in *Vegf-a* ^*Δ LysM*^ mice on CD, *n* = 6 in *Vegf-a* ^*ΔLysM*^ mice on HFHCD). **d** Tumors weight (*n* = 10 in WT mice on CD, *n* = 11 in WT mice on HFHCD, *n* = 10 in *Vegf-a* ^*Δ LysM*^ mice on CD, *n* = 16 in *Vegf-a *^*ΔLysM*^ mice on HFHCD, two independent experiments combined. Analysis of difference within groups were performed with two-sided Mann–Whitney *t*-test). **e** Correlation of VEGF-A concentration in tumor with tumor weight, in VEGF-A^ΔLysM^ mice and control littermates, under CD and HFHCD. **f** Laser Doppler perfusion imaging (PDPI) of tumors at day 9 (Right: representative picture). Mice were positioned on their back on a light-absorbing pad. LDPI image post-processing and measurement standardized protocol: the mean intensity of the Doppler signal was registered in ROI (Region of interest) encompassing the tumor and expressed as numerical value normalized for their area. (*n* = 3 mice/group in WT mice on CD, *n* = 6 in WT mice on HFHCD, *n* = 3 in *Vegf-a* ^*ΔLysM*^ mice on CD, *n* = 6 in *Vegf-a *^*ΔLysM*^ mice on HFHCD. Analysis of difference within groups were performed with two-sided Mann–Whitney *t*-test). **g** ICAM-1 level measured in total tumors of *Vegf-a *^ΔLysM^ mice on CD (*n* = 4) and HFHCD (*n* = 6) by bead-based multiplex immunoassay. Mean ± s.e.m. Two-sided Mann–Whitney *t*-test and Spearman correlation. Source data are provided as a Source Data file.

We performed a transcriptome analysis of total tumors in response to HFHCD: 1935 genes were differentially expressed, 1076 upregulated, including *Vegf-a* (fold change 3.4, *p* = 0.018) and 859 downregulated in tumors from HFHCD group (Fig. 5a, top 100 differentially expressed genes are shown in heatmap in Supplementary Fig. 6 and data file is available through GEO Series accession number GSE211392). Interestingly, despite the use of tumor bulk transcriptomes, “regulation of myeloid leukocyte differentiation” and “negative regulation of myeloid leukocyte differentiation” pathways were enriched (*p* = 0.013 and 0.015 respectively), as well as several metabolic pathways (“low-density lipoprotein particle remodeling”, “glycerolipid metabolic process”, “phospholipid metabolic process”) and autophagy (Top genes are colored in Fig. 5a).

In line with those data, luminex assay showed that VEGF-A (Fig. 5b), but not IL-1β and TNF-α (Supplementary Fig. 7a), was significantly increased in supernatants of MDSCs from HFHCD group in comparison to the CD group. We therefore hypothesized that myeloid-derived VEGF-A within the tumor microenvironment may contribute to tumor growth under HFHCD. We used *LysMCre*^*+/−*^*/Vegf-a *^*f/f*^ (*Vegf-a *^*ΔLysM*^) mutant mice with a specific deletion of VEGF-A in the myeloid cell compartment, and *LysMCre*^*−/−*^*/Vegf-a *^*f/f*^ (WT) as control littermate mice. Mouse weight, blood cell numbers (not shown) and cholesterol levels were unaffected by VEGF-A deletion in myeloid cells (Supplementary Fig. 7b). VEGF-A production was increased in tumors of WT mice fed a HFHCD, and was abolished in tumors of *Vegf-a *^*ΔLysM*^ mice under HFHCD (Fig. 5c), suggesting that the increase in VEGF-A in tumors in response to HFHCD was mainly of myeloid cell origin. In addition, the absence of myeloid-derived VEGF-A inhibited HFHCD-induced tumor growth amplification, suggesting that the pro-tumoral activity of myeloid cells under HFHCD is VEGF-A-dependent (Fig. 5d). The positive correlation between the levels of VEGF-A protein in tumors and the weight of tumors emphasizes VEGF-A involvement in tumor growth (Fig. 5e). VEGF-A deletion in myeloid cells maintained similar tumor vessel perfusion in both groups, as assessed by echo Doppler imaging (Fig. 5f). In agreement with previous studies showing that VEGF-A can inhibit ICAM-1 expression^35^, we found an increase in ICAM-1 total expression in the tumor in response to HFHCD (Fig. 5g). In vitro stimulation of bone marrow-derived cells and B16-F10 cells with VEGF-A or IL-1β did not influence the production of IL-1β and VEGF-A respectively (Supplementary Fig. 7c, d). Altogether, these data show that both IL-1β and VEGF-A orchestrate HFHCD-induced tumor growth acceleration through independent mechanisms.

### Switching HFHCD to CD prevents the deleterious effect of HFHCD on tumor growth

**Fig. 6:**
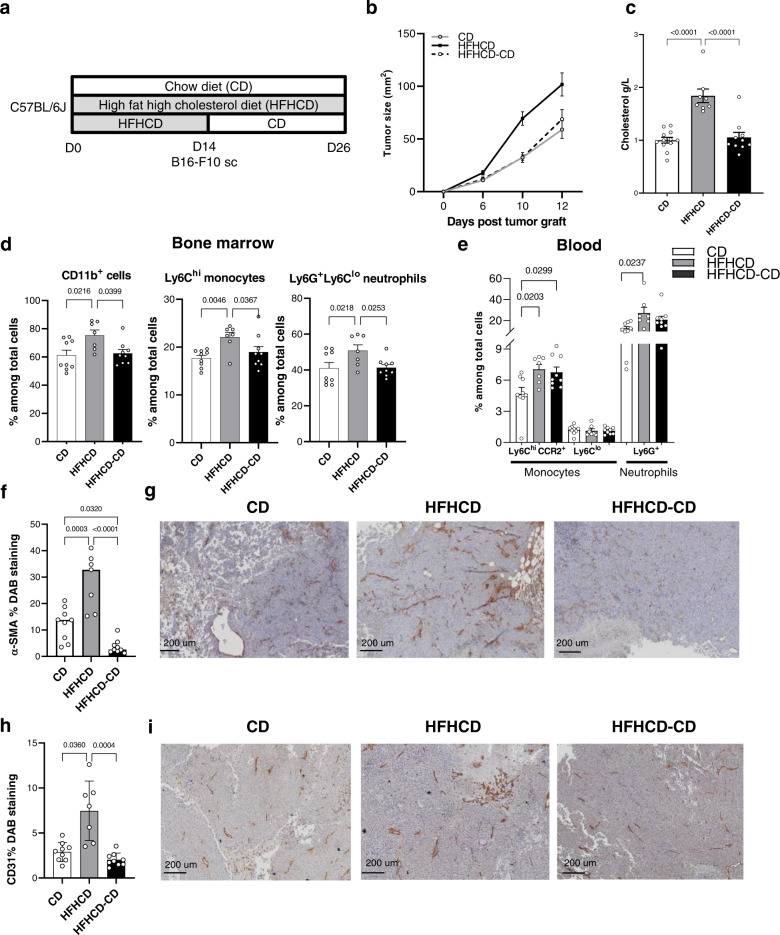
Switching diet avoid the effect of HFHCD on tumor growth. C57BL/6J mice were fed for 2 weeks with CD or HFHCD, part of HFHCD –fed mice was switched to CD the day of tumor graft. Mice were transplanted with 0,25 × 10^6^ B16-F10 cells (s.c.) and sacrificed at day 12 post injection. **a** Experimental design. **b** Tumor size evolution over time (at day 10 *p* = 0.0003 CD versus HFHCD and *p* = 0.002 HFHCD-CD versus HFHCD; at day 12 *p* = 0.014 CD versus HFHCD and *p* = 0.010 HFHCD-CD versus HFHCD). **c** Plasmatic cholesterol concentration (*n* = 12 in CD group, *n* = 8 in HFHCD group, *n* = 10 in HFHCD-CD group). **d** Flow cytometry analysis of (%) CD11b^+^myeloid cells, Ly6C^hi^ monocytes and Ly6G^+^ neutrophils in the bone marrow and **e** (%) of inflammatory monocytes (Ly6C^hi^ CCR2^+^), non-classical monocytes (Ly6C^lo^) and neutrophils (Ly6G^+^Ly6C^lo^) in blood. **f**–**i** Immunohistochemical staining on FFPE tumors. **f** Quantification of α-SMA DAB staining and **g** representative pictures of tumor tissue sections. Scale bar 200 mm. **h** quantification of CD31 DAB staining and **i** representative pictures of tumor tissue sections. Scale bar 200 mm. Data are expressed as mean ± s.e.m., one-way ANOVA with Tukey’s multiple comparisons test. *N* = 9 in CD group, *n* = 7 in HFHCD group, *n* = 9 in HFHCD-CD group, unless specified. Source data are provided as a Source Data file.

Finally, we wondered whether HFHCD discontinuation would impact on tumor growth. C57BL/6J mice were fed a CD or HFHCD for 2 weeks; after which a third group of mice under HFHCD was switched back to CD. B16-F10 cells were injected to the three groups (Fig. 6a). Switching HFHCD diet to the CD at the time of tumor cell injection protected against HFHCD tumor growth acceleration overtime, and was comparable to mice under CD (Fig. 6b). Switching to a normal CD normalized blood cholesterol levels and reversed the HFHCD-induced increase of Ly6C^hi^ monocytes and neutrophils in the bone marrow (Fig. 6c, d). At the same time, myeloid progenitors in bone marrow tended to normalize as well (Supplementary Fig. 8a, b). The level of circulating myeloid cells remained elevated at the end of the experiment (Fig. 6e). In situ immunostainings on paraffin-embedded tumor sections revealed that the angiogenic markers CD31 and smooth muscle actin (SMA), increased by HFHCD, were normalized to the CD level, when switching HFHCD to CD (Fig. 6f–i). Interestingly, this was paralleled by a significant increase in plasma levels of VEGF-A in the HFHCD group when compared to the CD group and a decrease when mice under HFHCD were transitioned to CD (Supplementary Fig. 8c). Therefore, flipping the early metabolic switch effectively protects against HFHCD tumor exacerbation.

## Discussion

Chronic low-grade inflammation, well characterized in individuals with metabolic disorders, has become an interesting therapeutic target in oncology. Here, we report that low-grade inflammation, caused by an atherogenic HFHCD, accelerates the growth of melanoma in mice. Detailed phenotyping revealed that HFHCD drives Ly6C^hi^ monocytosis, which directly supplied myeloid-derived cell accumulation into the growing tumor, a process controlled by IL-1β and driven by chemokine production. Under HFHCD condition, MDSCs promoted tumor growth through higher immunosuppressive capacities and increased VEGF-A production. We demonstrated, using tracking techniques, that Ly6C^hi^ monocytes are recruited into the tumor, with more efficient capacities when mice are under HFHCD. This is in line with studies showing that MDSCs arise from circulating monocytes and neutrophils, and does not rule out the possibility of accumulation of resident myeloid cells or circulating precursors^17,36^, which was not addressed in this study. We then used several strategies to limit myeloid cell accumulation in the tumor, to directly point out its role in tumor development, in response to HFHCD. Beside its undisputed effect on myeloid cell accumulation in the tumor, we found that HFHCD probably also directly affected tumor cell proliferation by activating lipogenesis, an anabolic pathway that may be favored even in the presence of exogenous lipid sources^37^. Importantly, our data showed that VEGF-A expression was boosted in tumors and plasma of mice on HFHCD, and that was mainly due to excessive myeloid accumulation in the tumor microenvironment at early time points. Normalizing dyslipidemia and monocyte production at the time of tumor cell injection, by switching HFHCD to CD, efficiently protected against cancer exacerbation. This happened despite moderate effect on blood monocyte count, which was also observed in another study where obese HFD discontinuation efficiently induced weight loss^38^.

The model of deleterious enrichment of myeloid cells in inflammatory tissues through a surplus of monocytes has been documented in cardiovascular diseases, where resolution of inflammation is altered after HFHCD^39–42^. However, in cancer, only a few studies previously pointed out the effect of Western diet on tumorigenesis in C57BL/6J mice, but they focused on tumor cells, not on host immune cells^43,44^.

In our present work, we propose the concept that silent mild-dyslipidemia without obesity creates a favorable environment for the accumulation of pro-tumoral myeloid cells, leading to tumor growth. This echoes a recent publication where irreversible acute inflammation, induced by myocardial infarction, accelerates breast cancer in mice through increase of circulating Ly6C^hi^ monocyte levels^45^. Herein, we demonstrate the deleterious, but reversible, low-grade inflammation impacts tumor development. Our work also emphasizes the sequential roles of IL-1β and VEGF-A; while systemic delivery of IL-1β would favor the production of myeloid cells, VEGF-A would shape the tumor microenvironment.

The recent emergence and efficacy of immunotherapy, in particular in melanoma, aimed to stimulate the immune system and fight cancer cells, has led to the need of understanding how immune cells are educated in the systemic and local pro-tumoral environments. Moreover, growing recognition of the potential contributions of innate immune effectors to anti-tumor immunity, especially in the context of combination immunotherapies, opens the way to novel therapeutic strategies aimed to generate a more integrated immune response against cancer^46^. Since innate cells are central actors of the inflammatory response, it is most likely that their contribution is enhanced during chronic inflammatory conditions.

Our present study paves the way to new opportunities in the field of immunotherapy, targeting Ly6C^hi^ monocytes in individuals with known low-grade inflammation, in particular in the context of mild-dyslipidemia. The importance of monocyte subsets was recently pointed out in several publications, suggesting their importance in cancer therapy. For instance, classical Ly6C^hi^ monocytes were proposed to predict response to anti-PD-1 immunotherapy^47^, and pro-angiogenic non-classical Ly6C^lo^ monocytes were thought to contribute to resistance against anti-VEGF therapies in mouse models of colorectal cancer, and in human^15,48^. These discoveries might even be more relevant in the context of low-grade chronic inflammation, as we found a predominant role of monocytes on tumor growth in this particular context. Remarkably, we found that both pharmacologic and immunotherapy treatments limited tumor development in mice under HFHCD, whereas they had minimal anti-tumor effects in mice under CD. Although we do not have clear explanations for that, several possibilities can be raised. First, excess of VEGF-A in the tumor, which is known to increase hyperpermeability and interstitial fluid pressure, may have ameliorated antibody diffusion within the tumor of mice under HFHCD. Second, the increased availability of myeloid cells in the circulation of mice under HFHCD may have facilitated their targeting. Third, modulation of gut microbiota by HFHCD could have ameliorated the response to treatments herein outlined, as specific commensals are related to treatment effectiveness in patients with melanoma^49^.

As we found that MDSCs from HFHCD fed mice tended to be more immunosuppressive, it would be interesting to evaluate the link, in conditions of chronic low-grade inflammation, between VEGF-A and the expression of inhibitory checkpoints on immune cells, as VEGF-A produced in the tumor microenvironment was recently shown to enhance expression of several inhibitory checkpoints involved in CD8^+^ T cell exhaustion^50^. In view of these results, association of anti-angiogenic molecules or statins with immunomodulators of inhibitory checkpoints may be of particular interest in solid tumors in individuals with pre-metabolic syndrome status.

## Methods

### Animals

All experiments were conducted according to European Community for experimental animal use guidelines (EC2010/63), and have been approved by the Ethical committee of the University Paris Descartes (CEEA 34) and the French Ministry of Agriculture (APAFIS #17112- 2018040515553905 – V3). Female mice (JAX strain, Charles River) were purchased at the age of 6 weeks; they were left for resting for 1 week. Mice were then either fed with specific diets for 2 weeks and sacrificed at the age of 9 weeks, or they were implanted with cancer cell lines at the age of 9 weeks (after 2-week diet) and sacrificed as specified in each experiment. OT-1 mice (CD8 transgenic Tcr for ovalbumin _257-264_) were also purchased from Charles River. *IL-1β*^*−/−*^ mice were kindly given by Pr Yoichiro Iwakura (Tokyo University of Science, Japan). *IL1R1 flox* mice were given by Dr. Pinteaux. *LysM*Cre^−/−^*Vegf-a *^*f*^^/^^*f*^ (WT) and *LysMCre*^*+/−*^*Vegf-a *^*f/f*^ *(Vegf-a *^*ΔLysM*^) mice were provided by Dr Christian Stockmann. All mice were on C57BL/6J background.

Mouse breeding occurred in our animal facility in accordance with local recommendations. Control mice were matched with littermates of the appropriate, age, sex, and genetic background to account for any variation in data, when specified. Mice received a standard chow diet (Safe, A03), a pro-obese high fat diet (Ssniff DIO-60 kJ% fat, Catalog No. E15742-347) or pro-atherogenic high fat/high cholesterol diet (Ssniff Paigen mod., 15% cocoa butter and 1.25% cholesterol, Catalog No. E15106-347) (Supplemental Table 1). Mice were housed under a 12 h light-dark cycle with ad libitum access to food and water, 50–70% humidity, and 18–22 °C ambient temperature. Tumor size never reached the maximum allowable size of 20 mm in diameter. Mouse euthanasia was performed by cervical dislocation after anesthesia with 4% isoflurane (IsoVet 100%; Centravet, France) in 100% oxygen in an anesthetic chamber which was not prefilled to prevent distress. Plasma cholesterol was measured using a commercial kit (DiaSys Cholesterol FS*, Germany).

### Cell line and tumor challenge

The B16-F10 was purchased from ATCC (CRL6475) and authentified by the manufactor (cytochrome oxidase 1 (CO1) barcoding assay). TC-1 cells (non-commercial cell line) expressing the HPV16 E6-E7 proteins were obtained from the laboratory of T.C. Wu (Dept of Pathology, School of Medicine, Johns Hopkins University, Baltimore, MD). Cells were cultured in RPMI supplemented with 10% fetal bovine serum (FBS), 2 mM glutamine, 50 U/ml penicillin and 100 μg/ml streptomycin, 1% sodium pyruvate, 1% MEM NAA and 1% 2-mercaptoethanol (RPMI complete medium). Mice were subcutaneously (s.c.) injected with 0.25 × 10^6^ B16-F10 or TC1 cells in 100 µl of saline buffer in the shaved abdominal flank. Tumor growth was monitored every 2–3 days using a caliper until 15 days post-injection. Tumor size was calculated as: width × length in mm^2^.

### Cells isolation, staining, and flow cytometry

Blood samples were collected in EDTA tubes by submandibular punction with 20-gauge needle under isofluorane anesthesia. Leukocytes were counted with Turk’s solution. Spleens were dissected and pressed through a 40‐μM cell strainer. Tumor were isolated from mice, minced and placed into GentleMACS C-tube with PBS-2%FCS, dissociated mechanically with GentleMACS dissociator (Miltenyi) according to manufacturer's standard protocol, then filtered on 70um strainer. Red blood cells were lysed with a osmotic lysis buffer (Ammonium-Chloride-Potassium). Bone marrow from both tibias was harvested, and the cells were collected by inserting a needle into the bone and washing with PBS-2%FCS. Single-cell suspensions were first blocked with anti-CD16/32 antibody (93; eBioscience) 5 min 4 °C, (except for bone marrow staining), then stained for surface molecules in PBS-2%FCS. For intracellular staining, after surface staining, cells were permeabilized using the FoxP3/transcription Factor staining buffer set (eBiosciences) according to manufacturer's protocol, then stained with intracellular mAb. For intracellular cytokines staining, cells were first incubated 4 h at 37 °C in the presence of Golgistop (Monensin) and Golgiplug (Brefeldin A) (both from BD Biosciences) in complete medium, then staining for surface molecules and intracellular staining as described above (Supplemental Table 2).

Dead cells were excluded using live/dead fixable aqua dead cell kit (Invitrogen). Samples were acquired on Fortessa X20 and on a LSR Fortessa Analyser (BD Biosciences) and analyzed with FlowJo software (V10).

For Dimensionality reduction using t-SNE and automatic clustering: CD45 live cells were manually gated from multicolor flow cytometry and exported in a FCS file. T-Distributed Stochastic Neighbor Embedding (t-SNE) dimensionality reduction was performed using BH t-SNE, an implementation of t-SNE via Barnes-Hut approximations. 20,000 events were used for t-SNE dimensionality reduction.

### Gene expression analysis

For the transcriptomic analysis, 4 tumors per group were harvested from mice fed with CD or HFHCD at Day 9 post injection of B16-F10 melanoma cells. Tumors were minced and lysed into RLT buffer containing 1% β-mercaptoethanol. RNA was extracted using Qiagen RNeasy kit according to the manufacturer’s instructions.

Analysis of gene was done by Affymetrix MouseGene2.0ST array at the GENOM’IC core facility (Cochin Institute). *P*-values were obtained by a two-sided Student’s t-test with group variance within limma package. Complete analysis is provided through GEO Series accession number GSE211392 (https://www.ncbi.nlm.nih.gov/geo/query/acc.cgi?acc=GSE211392). Genes with *p*-value < 0.05 were used for enrichment pathway analysis with R library clusterProfiler with Gene Ontology knowledgebase^51^.

### In vivo cells labeling

For intravital neutrophil and monocyte labeling, mice were retro-orbitally injected i.v with anti-Ly6G PE and anti-CD115 APC (5μg each) on day 8 post tumors injection. 6 h later mice were sacrificed. Labeled cells were analyzed in blood and tumors by flow cytometry.

### Monocytes adoptive transfer into tumor-bearing mice

Bone marrows from CD45.1 mice (Charles River) fed 2 weeks with CD or HFHCD, were collected and monocytes were isolated by magnetic negative selection (EasySep™ Mouse Monocyte Isolation Kit, Stem cell). Purity was checked by flow cytometry (>90%) before injection. 10^6^ cells were intravenously injected into B16-F10 tumor-bearing recipient mice (C57Bl6/J mice CD45.2), fed with CD or HFHCD, on day 11 after tumor graft. Mice were killed 18 h after injection and quantification of CD45.1 monocytes number in CD45.2 tumors was done by flow cytometry.

### In vitro cell culture

Bone marrow cells were collected from femurs and tibias by insertion of a needle into the bone and flushing with RPMI supplemented with 0.2% BSA and 1% FCS as previously described. Macrophages were differentiated after culture for 9 days with 20% L929 conditioned medium.

BM-DM were cultured in RPMI 1640 supplemented with Glutamax, 10% FCS, 0.02 mmol/L *β*‐mercaptoethanol and antibiotics Penicillin and Streptomycin. Cells were stimulated in RPMI complete medium with LPS (10 µg/ml, Sigma Aldrich), mIFNγ (100 U/ml) then supernatant was harvested. Cytokines were measured by ELISA (R&D Systems).

For B16-F10 in vitro proliferation, 10 000 B16-F10 cells were co-cultured with 2500 CD45 cells isolated from tumors or with BM-DM, in RPMI complete medium. 24 h later, B16-F10 proliferation was measured by 3T-Thymidine incorporation or MTT assay (Sigma). For Thymidine incorporation one Ci [^3^H] thymidine (Amersham) was added to each well during the last 18 h. Thymidine incorporation was assessed using a TopCount NXT scintillation counter (Perkin Elmer).

### MDSC isolation and assay

For suppressive proliferation assay, MDSC (CD11b^+^ Ly6C^+^ Ly6G^+^) enriched cells were obtained from spleen of tumor-bearing mice by magnetic sort (Myeloid-Derived Suppressor Cell Isolation Kit, Miltenyi). CD8 T cells were isolated from spleen of OT-I mice by magnetic sort (CD8a + T Cell Isolation Kit, Miltenyi), and labeled with 5 μM CFSE (Vybrant™ CFDA SE Cell Tracer Kit, Invitrogen). OT-I CD8 T cells were incubated with OVA _257-264_ (SIINFEKL) at 0.001μg/ml (Polypeptide) with various ratio of MDSC for 72 h. On day 3, CFSE dilution was analyzed by flow cytometry.

The percentage of proliferating cells was then used to calculate the percent suppression of proliferation. Percent suppression of proliferation was calculated using the following formula: (1 − (% proliferation with MDSC /% proliferation without MDSC))* 100.

For isolation of MDSCs, tumors were harvested and dissociated as described before, then dead cells were removed with EasySep™ Dead Cell Removal Kit (StemCell). MDSC (CD11b^+^Gr1^+^) were enriched by magnetic negative selection (EasySep™ Mouse MDSC Isolation Kit, StemCell). Purity was checked by flow cytometry (>88%). 40,000 cells were cultured in RPMI complete medium for 24 h, then supernatant were collected for multiplex immunoassays, and cells harvested and lysed with RLT lysis buffer (Qiagen) for RNA extraction.

### Cytokines and chemokines assay, ELISA

Total protein from tumors was extracted using Bio-plex lysis buffer (Bio-Rad) according to manufacturer's instructions, and concentration was determinated with BCA assay (Pierce).

The bead-based multiplex immunoassay (Luminex) was used to measure the levels inflammatory and angiogenesis mediators from tumors protein lysates or from supernatant of MDSCs isolated from tumors. A 23-plex inflammatory immunoassay panel (IL-1α, IL-1β, IL-2, IL-3, IL-4, IL-5, IL-6, IL-9, IL-10, IL-12 (p40), IL-12 (p70), IL-13, IL-17A, Eotaxin, G-CSF, GM-CSF, IFN-γ, KC, MCP-1 (MCAF), MIP-1α, MIP-1β, RANTES, TNF-α) (Bio-Rad) and 5-plex angiogenesis immunoassay panel (PDFG-a, Endoglin, VEGFR2, Fas-L, ICAM-1)(R&D systems), were performed. A bead-based multiplex immunoassay for GM-CSF, G-CSF, M-CSF, SDF-1/CXCL12, and Leptin was assessed on bone marrow supernatant and on blood plasma (R&D systems). All immunoassays were performed according to manufacturer's protocol and analyzed on Bio-Plex 200 V6.1 (Bio-rad). The analytes concentration was calculated using a standard curve (5 PL regression), with Bioplex 200 manager software (Bio-Rad). Non-detectable or non-relevant cytokines were not presented. TNF-α, IL-12, IL−10, IL-1β were evaluated by ELISA (R&D system) for BMDM supernatant. VEGF-A concentration was evaluated by ELISA (mouse VEGF DuoSet, R&D system) or by bead-based multiplex immunoassay (R&D Biotechne) in tumors lysates and in plasma. For ELISA, analysis were done on Spectrostar microplate reader (BMG labtech), analytes concentration was calculated using a standard curve with linear regression (R^2^ > 0.99) with MARS Data Analysis Software V5.

### Immunohistochemistry

Formalin-fixed paraffin-embedded (FFPE) tumors were sliced at a thickness of 4 µm with a microtome. Tissue sections were deparaffinized with xylene (VWR Chemicals, 28973.294) and rehydrated with bath of decreasing concentration of ethanol (VWR Chemicals, 20821.365 P). Then, a Heat-Induced Epitope Retrieval was performed according manufacturer of primary antibody (buffer pH6 or pH9) during 20 min at 95 °C.

Immunohistochemistry was realized with the detection kit “Mouse and rabbit specific HRP/DAB and Abcam primary antibodies: a rabbit monoclonal CD31, a rabbit monoclonal Ki67 and a rabbit monoclonal alpha smooth muscle Actin (Supplemental table 2).

A step of permeabilizing was done with PBS + 1% of Triton X-100 and endogenous peroxydases and non-specific binding sites were blocked respectively with a ready-to-use hydrogen peroxide blocking reagent and a protein blocking reagent (Abcam, ab126466). Primary antibodies were incubated overnight (anti-Ki67: 1/200, anti-CD31: 1/2000 and anti-αSMA: 1/2000) in humidify chamber. Secondary antibody is a ready-to-use HRP micro-polymer conjugated goat anti-rabbit and he was incubated 15 min at room temperature. Finally, the DAB chromogen/substrate of detection kit was used to reveal the secondary antibody location and sections were counterstained with hematoxylin and then cover-slipped.

The quantification of DAB staining was performed with ImageJ (FIJI) through a color deconvolution. Then, the software is able to detect and measure stained pixels. The quantification of DAB staining is obtained by dividing the section pixel measure by the stained pixels measure.

### Anti-CD115 neutralizing and antagonists treatments

For the treatment with CD115 antibody (inVivoMab Clone AFS98, BioXcell), mice were injected with 125 mg/kg one day before tumor injection, then twice a week until sacrifice, control mice receive 125 mg/kg of rat IgG2a (inVivoMab Rat, Clone − 2A3, BioXcell).

CXCR2 antagonist SB225002 (Tocris Bioscience) was administered at dose 2 mg/kg, and CCR2 antagonist BMS CCR2 (RD Biotechne) at 10 mg/kg, 6 days a week i.p. Treatment was started next day after tumor injection and continued until sacrifice. Control mice receive DMSO 20%-PBS.

### Doppler imaging and quantification

Mice were shaved, anesthetized with isoflurane and kept under monitored temperature during the time of imaging. Vessel density was evaluated by laser-Doppler perfusion imaging to assess in vivo tissue perfusion in the tumor (Moor Instrument). Quantification of the vessel density was performed with MoorLDIReview V6.1 software.

### Fatty acids analysis

Fatty acids were extracted from 50 mg homogenised tumor aliquots by the addition of 25 μl of 150 mmol/L sodium chloride, 25 μl of 10 mg/ml heptadecanoic acid as an internal standard (C17:0; Sigma, France) and 100 μl of methanol-chloroform mixture (1:1, v/v). The samples were centrifuged (10,000 × g for 10 min) and the lower chloroform phase containing the lipids was recovered. Two additional extractions each with 50 μl of chloroform were performed and chloroform phases recovered. The organic phases were dried under N2. One ml of toluene was added to dissolve the triglycerides. Total fatty acids were transesterified using 1 ml boron trifluoride in methanol (BF3; Sigma France) for 45 min at 100 °C^52^. Fatty acid methyl esters were extracted by adding 0.25 ml of water and 0.50 ml of cyclohexane, and analyzed by gas chromatography using an Agilent Technologies 7890 A instrument equipped with a splitless injector (20:1) and a fused silica Elite 225 capillary column (30 m × 0.32 mm, 0.25 μm, Perkin Elmer France). H2 was used as the carrier gas at 1 ml/min. The column temperature program started at 35 °C for 2 min, ramped from 15 °C/min to 180 °C for 2 min, then ramped from 10 °C/min to 220 °C for 10 min. The injector and flame ionisation detector temperatures were set at 250 °C. The individual GAs were identified by comparing their retention times with those of the standards: FAMEmix (cat 47885-U 37, Supelco and American Oil Chemists’ Society (AOCS) marine oil standard. The peak areas were integrated and corrected using the response factors for each fatty acid. Each fatty acid was expressed as a percentage of the total identified fatty acids (available through https://osf.io/te8s2/). The sum of the fatty acid contents expressed was assumed to reflect the total lipid content (free fatty acids, triacylglycerols and phospholipids).

### WST-1 proliferation assay

Cell proliferation was evaluated in triplicates by a colorimetric WST-1 assay (Abcam) according to the manufacturer’s protocol. B16-F10 cells were seeding (4 × 10^3^) in 96-well plates at 37 °C in 100 µL RPMI 1640 medium, 1% free fatty acid BSA (Sigma), 1% penicillin/streptomycin, 1% glutamine, 1% sodium pyruvate, 1% MEM NAA and 1% 2-mercaptoethanol. After 24 h, cells were stimulated with chloroform (diluted 1/2000 as control vehicle of cholesterol or 1/666 as control of mixed fatty acids) (VWR), cholesterol (50 µg/mL, Sigma) or a mix of fatty acids: palmitic acid, oleic acid, stearic acid (50 µM, VWR) for 72 h. 10 µl of WST-1 (10% of total volume) was added to the cells, and the cells were incubated for 3 h. The plate was read using iMark Microplate Reader (Bio-Rad) by measuring the absorbance of the dye at 450 nm, with 655 nm set as the reference wavelength. Averages of the absorbance values were calculated and plotted.

### Real-time PCR

Total RNA was isolated using Purelink RNA mini kit (Thermofisher Scientific). The first-strand cDNA was prepared using Maxima First Strand cDNA Synthesis Kit for RT-qPCR, with dsDNase (Thermofisher Scientific) according to the manufacturer’s protocol up to 1 μg of total RNA. All RT-PCR reactions were performed in a 20 μl mixture containing 1× Power SYBR™ Green PCR Master Mix (Thermofisher Scientific), 0.5 μmol/L of each primer, and 4 μl of cDNA template. Primers (Eurofins) for detection of mouse *Il-1β, Vegf-a, Il-6, Cxcl1, Cx3cl1, Cx3cr1, Ccl2, Ccr2, Ccl5, Ccr5, Glyceraldehyde 3-Phophate Dehydrogenase (GAPDH), Cxcr4* and *Rpl13A*. cDNAs are listed in Supplemental Table 2. Real-time PCR was performed using the Applied Biosystem 7500 Fast system under the following cycling conditions: 95 °C for 10 min, 40 cycles of 95 °C for 15 s, and 60 °C for 1 min, followed by the melting curve stage. The relative RNA expression level was normalized to that of GAPDH except for *Cxcr4* normalized to *Rpl13A*.

### Bone marrow-derived macrophage and B16-F10 stimulations

Primary macrophages were derived from mouse bone marrow-derived cells (BMDM). Tibias and femurs of mice were dissected and their marrow flushed out. Cells were seeding (500 × 10^3^) in 12-well plates and grown for 7 days at 37 °C in 1 mL of RPMI 1640 medium (Sigma), 20% FCS (*PAN*-*Biotech*), 1% penicillin/streptomycin (Eurobio), 1% glutamine (Gibco) and 50 ng/ml M-CSF (BioLegend). Cancer cell line B16F10 in 1 mL RPMI 1640 medium, 20% FCS, 1% penicillin/streptomycin, 1% glutamine, 1% sodium pyruvate (Gibco), 1% MEM NAA (Gibco) and 1% 2-mercaptoethanol (Gibco) were seeded (100 × 10^3^) in 12-well plates. After 7 days or 24 h for BMDM or B16-F10 respectively, cells were stimulated overnight without or with LPS (100 ng/mL, Ozyme) + 30 last minutes ATP (5 mM, VWR), IL-1β (50 ng/mL, BioLegend) or VEGF-A (50 ng/mL, BioLegend). Culture supernatants were collected, centrifuged to pellet any detached cells, and measured using IL-1β (R&D Systems) or VEGF-A (R&D Systems) ELISA Kit. The ELISAs were performed according to the manufacturer’s instructions.

### Statistics

Graphs were generated and statistical analyses were performed with Prism software (GraphPad software, La Jolla). Results are expressed as means ± SEM. The Mann–Whitney *t*- or ANOVA tests were used as specified in the legends. Comparison between tumor growth curves have been performed using a two-way ANOVA test, and multiple comparisons have been corrected with the Bonferroni coefficient. The association between two variables was done by spearman correlation. *P* values < 0.05 was considered significant.

### Reporting summary

Further information on research design is available in the Nature Research Reporting Summary linked to this article.

## Supplementary information

Supplementary Information

Reporting Summary

Peer Review File

## Data Availability

All data are available in the article, supplementary information and source data. The lipidomic data generated in this study have been deposited in the Open Science Framework database under accession code https://osf.io/te8s2/. The transcriptomic data are accessible through GEO Series accession number GSE211392 Source data are provided with this paper.

## Source data

Source Data
