# Supplementary material for: Mild dyslipidemia accelerates tumorigenesis through expansion of Ly6Chi monocytes and differentiation to pro-angiogenic myeloid cells
Source: Nat Commun. 2022 Sep 14;13:5399. doi: 10.1038/s41467-022-33034-0 (PMC9475043; doi:10.1038/s41467-022-33034-0)
Supplement: Supplementary file 1 — Supplementary Information [file 41467_2022_33034_MOESM1_ESM.pdf]

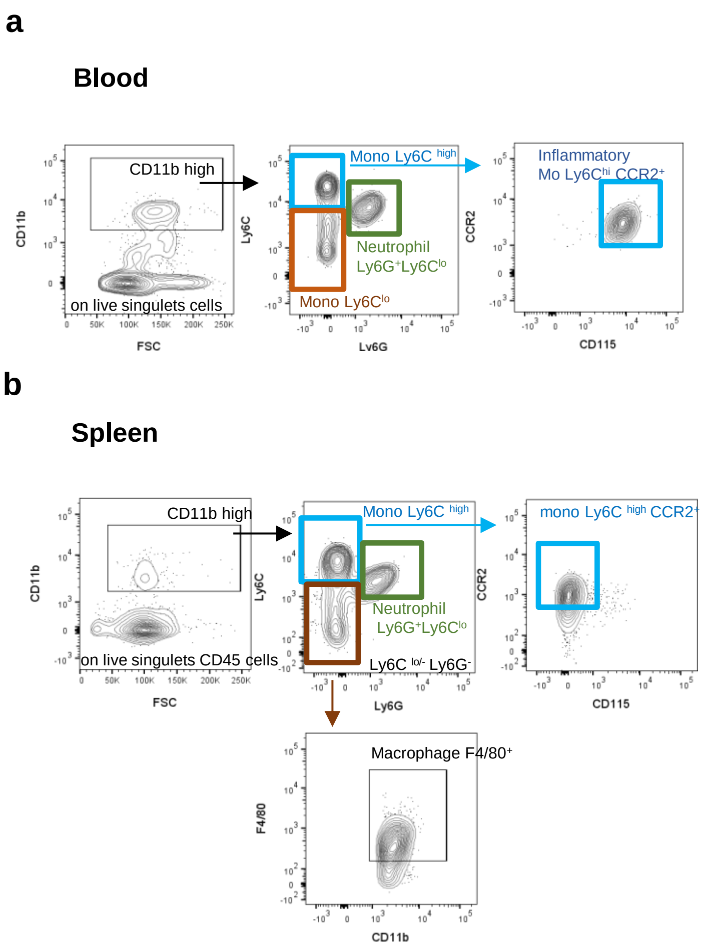

Supplementary Figure 1: Flow cytometry gating strategy

Flow cytometry gating strategy for myeloid subsets in **a)** blood and **b)** spleen.

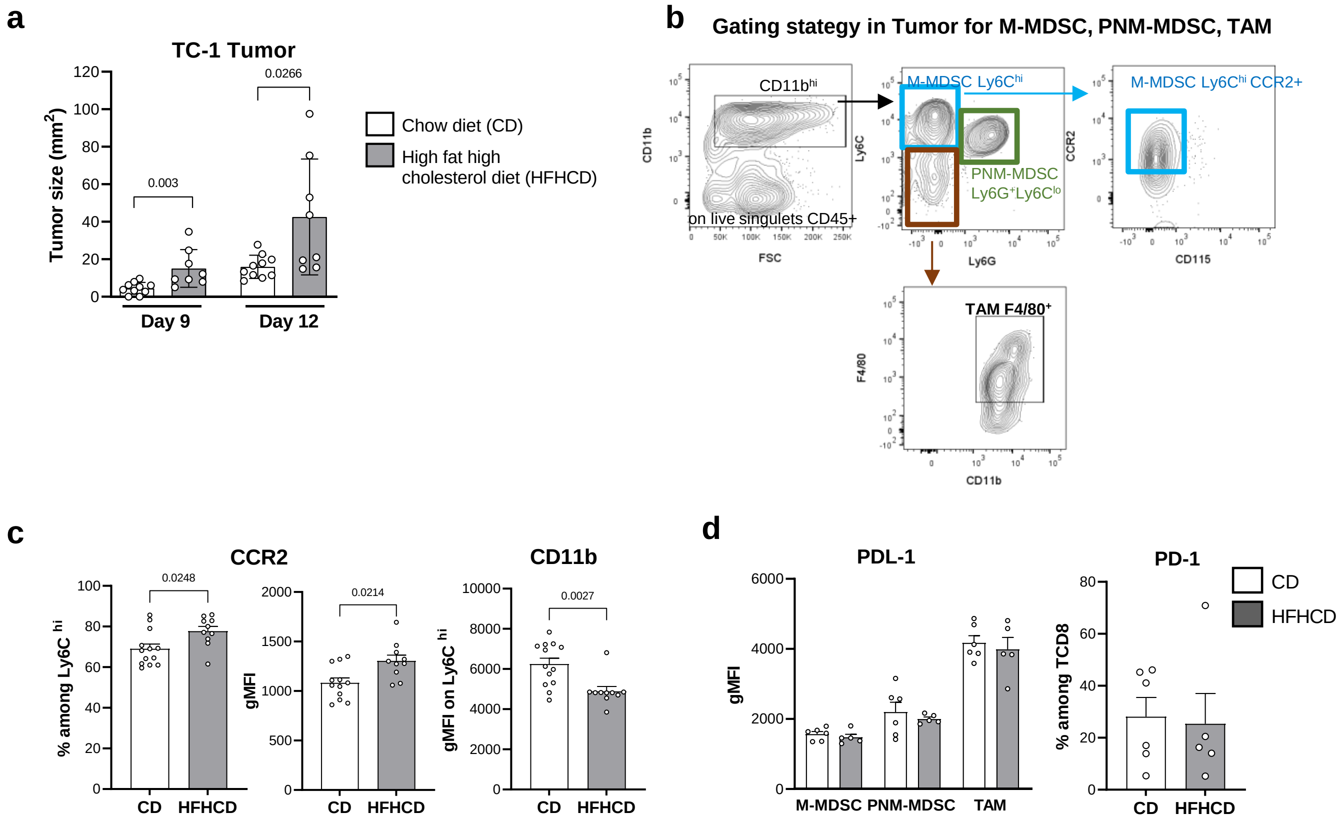

Supplementary Figure 2: Pro-atherogenic HFHCD accelerates B16-F10 melanoma growth in C57BL/6J mice
a) C57BL/6J mice were fed for 2 weeks with CD (n=10) or HFHCD (n=8), then grafted withTC-1 tumor cells (10^5 cells s.c). TC-1 tumor size (8-10 mice/group, pool of 2 independent experiments) **b-d** C57BL/6J mice were fed for 2 weeks with CD or HFHCD, then grafted with B16-F10 tumor cells **b)** Flow cytometry gating strategy in the tumor for M-MDSC ($CD11b^+Ly6C^{hi}$), PNM-MDSC ($CD11b^+Ly6C^{lo}$ $Ly6G^+$) and TAM ($CD11b^+Ly6C^{lo}$ $Ly6G^-$ $F4/80^+$). **c)** Flow cytometry analysis of expression of CCR2 (gMFI and %) *(left)* and CD11b among M-MDSC $Ly6C^{hi}$ in the tumor *(right),* n=13 in CD group, n=10 in HFHCD group. **d)** Flow cytometry analysis of expression of PDL1 (gMFI) among M-MDSC, PNM-MDSC and TAM *(left)* , and PD-1 (%) among CD8 T cells in the tumor *(right)*, *),* n=6 in CD group, n=5 in HFHCD group.. Datas are expressed as mean±sem. Analysis of difference within groups were performed with two-sided Mann-Whitney t-test. Source data are provided as a Source Data file.

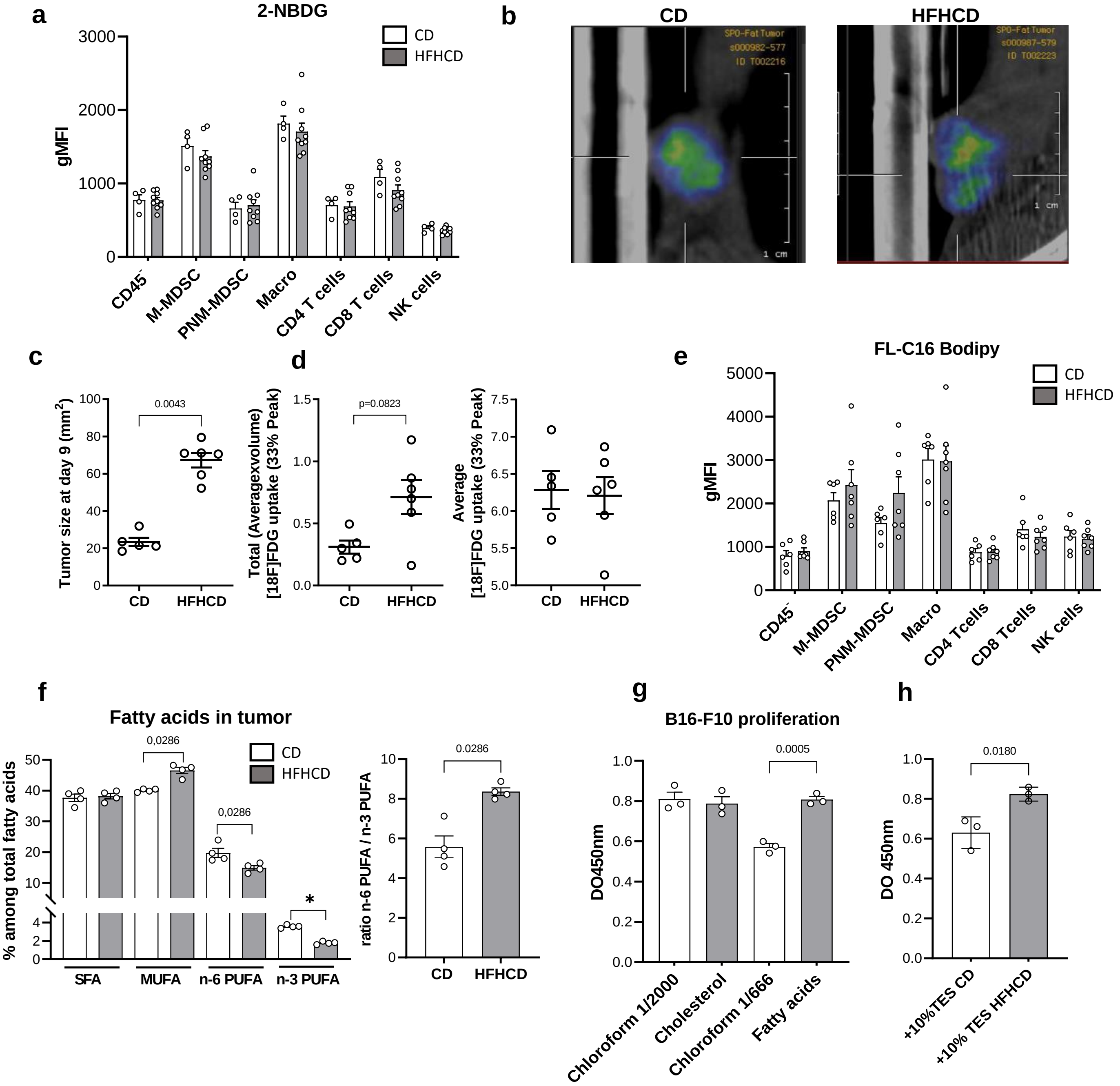

Supplementary Figure 3: Effect of HFHCD on metabolism and proliferation of tumor cells

C57BL/6J mice were fed for 2 weeks with CD or HFHCD, then grafted with B16-F10 cells (0.25x10^6 cells s.c) **a)** at D13-15 after tumor graft, mice were fasten overnight, then injected with an analogue of glucose 2-NBDG (200µg iv) 15min before sacrifice, n=4 in CD, n=9 in HFHCD. **b-d)** *in vivo* 18F FDG-PET monitoring in tumor bearing mice (n=5 in CD and n=6 in HFHCD group) day 9 post tumor cell injection, **b)** representative picture. **c)** tumor size **d)** 18F FDG total captation (left) and average captation (right) are shown, **e)** at D13-15 after tumor graft, mice were fasten overnight, then injected with FL-C16-BODIPY (50µg ip), mice were sacrificed 1h before sacrifice. Quantification of *in vivo* uptake of 2-NBDG or FL-C16-BODIPY(gMFI) by CD45 negative cells and main subset leukocytes infiltrating tumor was analyzed by flow cytometry. **f)** Composition (%) of Saturated fatty acids (SFA), Mono-unsaturated fatty acid (MUFA), Poly-unsaturated *n*-6 fatty acid (*n*-6 PUFA), Poly-unsaturated *n*-3 fatty acid (*n*-3 PUFA) and ratio *n*-6 PUFA/ *n*-3 PUFA in tumor by Gas chromatography with flame ionization detector (GC/FID). % of total SFA, MUFA, PUFA, was calculated by the sum of all FA of each family (*please see details in supplementary table 2)* (n=4 mice/group). The data generated in this study have been deposited in the Open Science Framework database under the DOI DOI 10.17605/OSF.IO/TE8S2. **g-h)** *in vitro* proliferation of B16-F10 cell (4x10^3cells/ well) cultured 72h in medium supplemented with **g)** cholesterol (50µg/ml) or control (Chloroform 1/2000), or with a mix of oleic, palmitic and stearic acid (50µM each) or control (Chloroform 1/666) or **h)** with10% of tumor explant supernatant (TES) from CD or HFHCD tumor (n=3 mice/group) (unpaired t-tests). Proliferation was measured by WST-1 colorimetric assay. Datas are expressed as mean±sem. Analysis of difference within groups were performed with two-sided Mann-Whitney t-test. Source data are provided as a Source Data file.

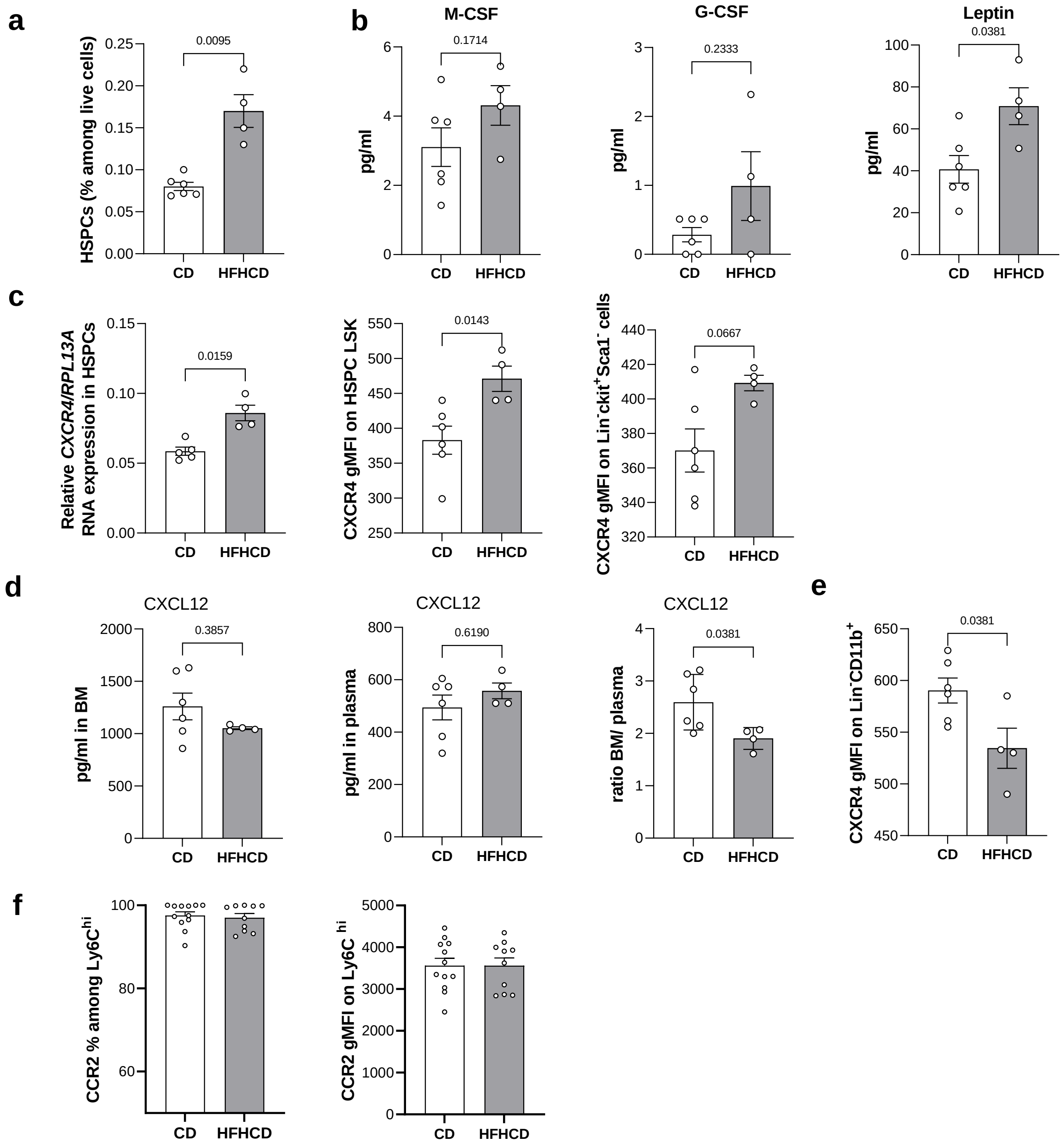

Supplementary Figure 4 : HFHCD stimulates the production and retention of HSPCs and favors the exit of monocytes from the bone marrow

C57BL/6J mice were fed for 2 weeks with CD (n=6 mice) or HFHCD (n=4 mice). **a)** Flow cytometry analysis of bone marrow HSPC level (Lin^-CD11b^- $ckit^+Sca1^+$). **b)** cytokines were measured in bone marrow by bead-based multiplex immunoassay. **c)** relative expression of Cxcr4 mRNA in HSPC isolated from bone marrow and expression of CXCR4 (gMFI) analyzed by flow cytometry in HSPC and myeloid progenitors (Lin^-ckit^+Sca1). **d)** CXCL12 production in the bone marrow and the plasma (multiplex assay). **e)** expression of CXCR4 (gMFI) in mature $CD11b^+$ cells in bone marrow. **f)** CCR2 expression level on blood $Ly6C^{hi}$ monocytes of mice on CD (n=12) and HFHCD (n=10). Data are expressed as mean±sem. Two-sided Mann-Whitney t-test. Source data are provided as a Source Data file.

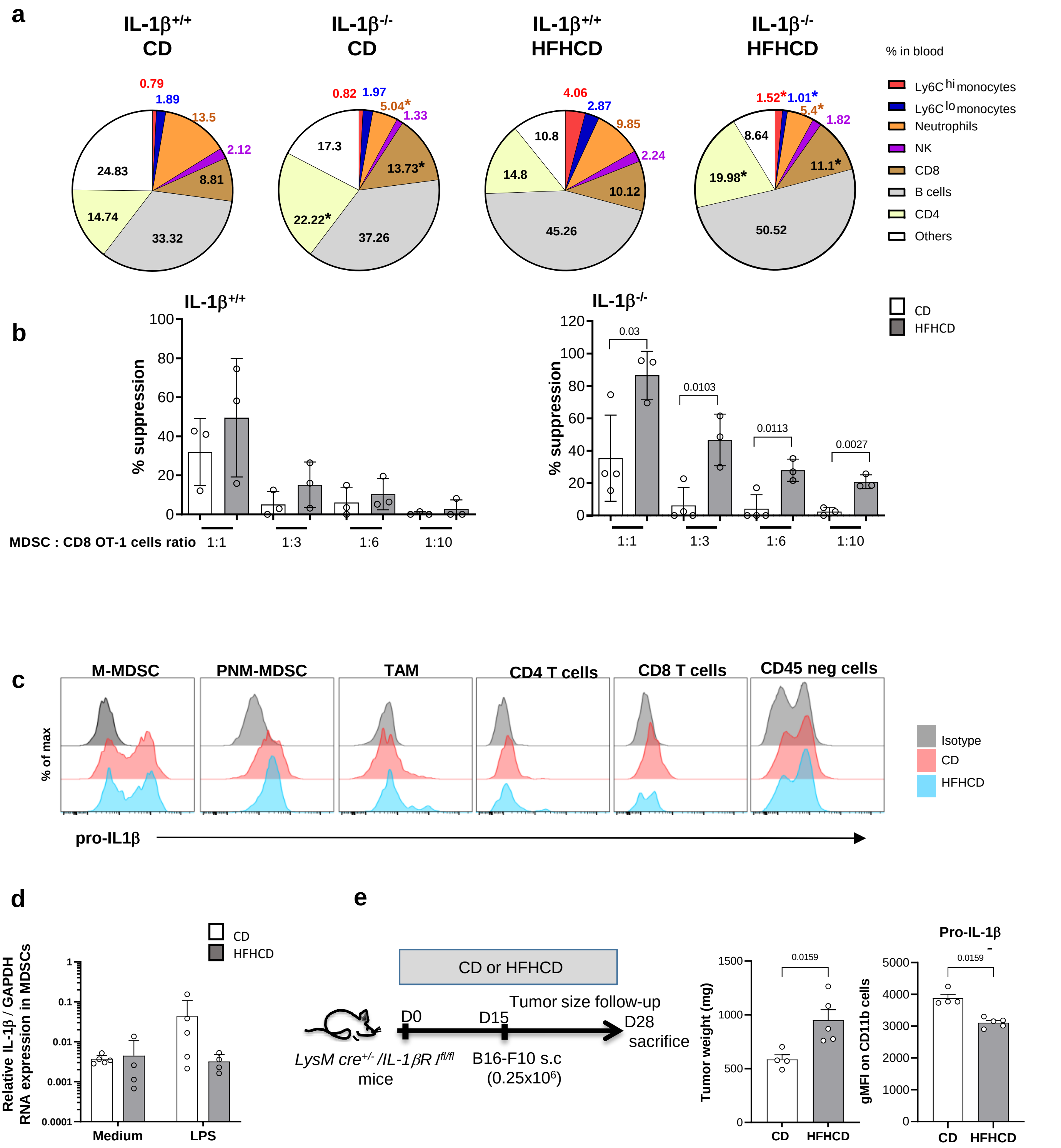

Supplementary Figure 5 : Impaired myeloid cell accumulation in the tumor limits tumor growth under HFHCD. **(a-b)** IL-1β$^{-/-}$ mice and control IL-1β$^{+/+}$mice were fed for 2 weeks with CD or HFHCD, then were transplanted with 0,25x10^{6} B16-F10 cells (s.c). Mice were sacrificed at day 15 post injection. **a)** Proportion of immune cells in blood are shown in the pie chart (n=5/group), statistical difference between the 2 groups, for the same leukocyte subset, was mentioned on the pie chart by * p<0,05. **b)** Suppressive activity of myeloid-derived suppressor cells (MDSC), isolated from spleen of *IL-1β$^{-/-}$* and *IL-1β$^{+/+}$* tumor-bearing mice, evaluated by CFSE dilution of CD8^{+} OT-I T cells incubated with OVA$_{257\text{-}264}$ (SIINFEKL, 0,001µg/ml). Proliferation was measured after 72 hours by flow cytometry. (n=3/group except IL-1β$^{-/-}$ on CD n=4; from 1 representative experiment of 2). **c)** Flow cytometry analysis of pro-IL1β expression (gMFI) among M-MDSCs, PNM-MDSCs, TAMs, CD4 T cells, CD8 T cells and CD45 negative cells in tumor CD or HFHCD. **d)** Relative expression of IL-1β mRNA in MDSCs isolated from tumor CD (n=5) or HFHCD (n=4) and stimulated overnight or not with LPS (1µg/ml). **e)** Mice with specific deletion of IL-1β receptor in myeloid cells (*LysM$^{cre+/-}$ IL-1R1$^{fl/fl}$*) were fed for 2 weeks with CD (n=4) or HFHCD (n=5), and transplanted with B16-F10 cells (s.c). *(left)* Experimental design, *(middle)* Tumor weight at day 14 post tumor graft and *(right)* flow cytometry analysis of pro-IL1β expression (gMFI) among CD11b^{+} myeloid cells in the tumor. (n=4-5 mice/group from 1 representative experiment). Data are expressed as mean±sem. Two-sided Mann-Whitney t-test. Source data are provided as a Source Data file.

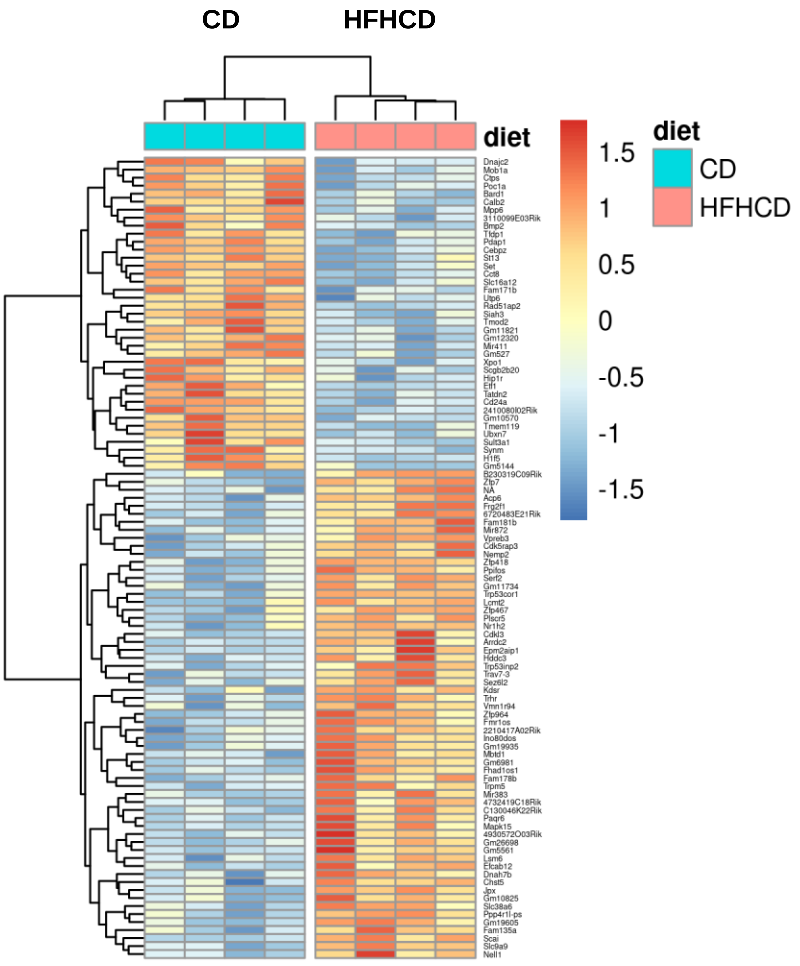

Supplementary Figure 6 : Transcriptomic analysis of melanoma tumors from CD versus HFHCD-treated mice.
Heatmap of top 100 genes differentially expressed between CD or HFHCD diet at day 9 post B16-F10 cell injection (n=4 mice/group). P-values were obtained by Student's t-test with group variance within limma package. The data generated in this study have been deposited in the Open Science Framework database under the DOI DOI 10.17605/OSF.IO/TE8S2

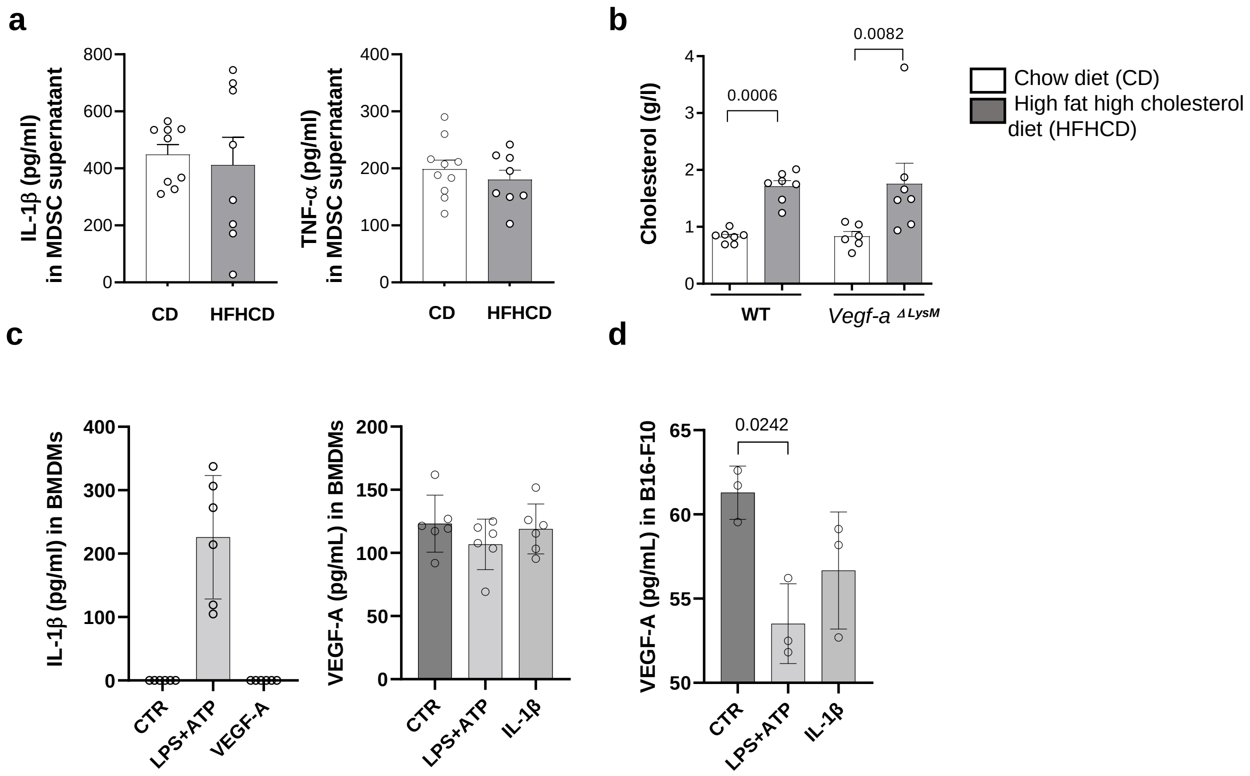

Supplementary figure 7: **Myeloid-derived VEGF-A controls tumor growth under HFHCD**
C57BL/6J mice, fed with CD or HFHCD and grafted with B16-F10 cells **a)** MDSCs were isolated from B16-F10 tumors at day 13 after tumor graft, and cultured for 18h in complete medium. IL-1β and TNF-α were measured by bead-based multiplex immunoassay in the supernatant (n=10 mice on CD and n=8 mice on HFHCD).**b)** *LysMCre*$^{+/-}$/ *Vegf-a* $^{f/f}$ (*Vegf-a* $^{\Delta LysM}$) and their control littermate *LysMCre*$^{-/-}$/ *Vegf-a* $^{f/f}$ (WT), fed with CD or HFHCD for 2 weeks, were transplanted subcutaneously with 0,25x10^{6} B16-F10 cells. Total cholesterol level in plasma measured by colorimetric assay. n=7 mice/group except for n=6 in *Vegf-a* $^{\Delta LysM}$ mice on CD. **c)** Bone-marrow-derived macrophages from C57Bl/6J WT (n=6) mice were stimulated with LPS (100ng/ml) + ATP (5mM), VEGF-A (50ng/ml), IL-1β (50ng/ml), or unstimulated (CTR) for 24h. IL1β *(left)* and VEGF-A *(right)* were measured in the supernatant by ELISA (n=6/group). **d)** B16-F10 cells were stimulated *in vitro* with LPS+ATP or with IL-1β or unstimulated (CTR) for 24h (same concentrations). VEGF-A were measured in the supernatant by ELISA (n=3 independent experiments). Data are expressed as mean±sem. Analysis of difference within groups were performed with Two-sided Mann-Whitney t-test (a-b), and with one-way ANOVA with Bonferroni's multiple adjustment test (c-d). Source data are provided as a Source Data file.

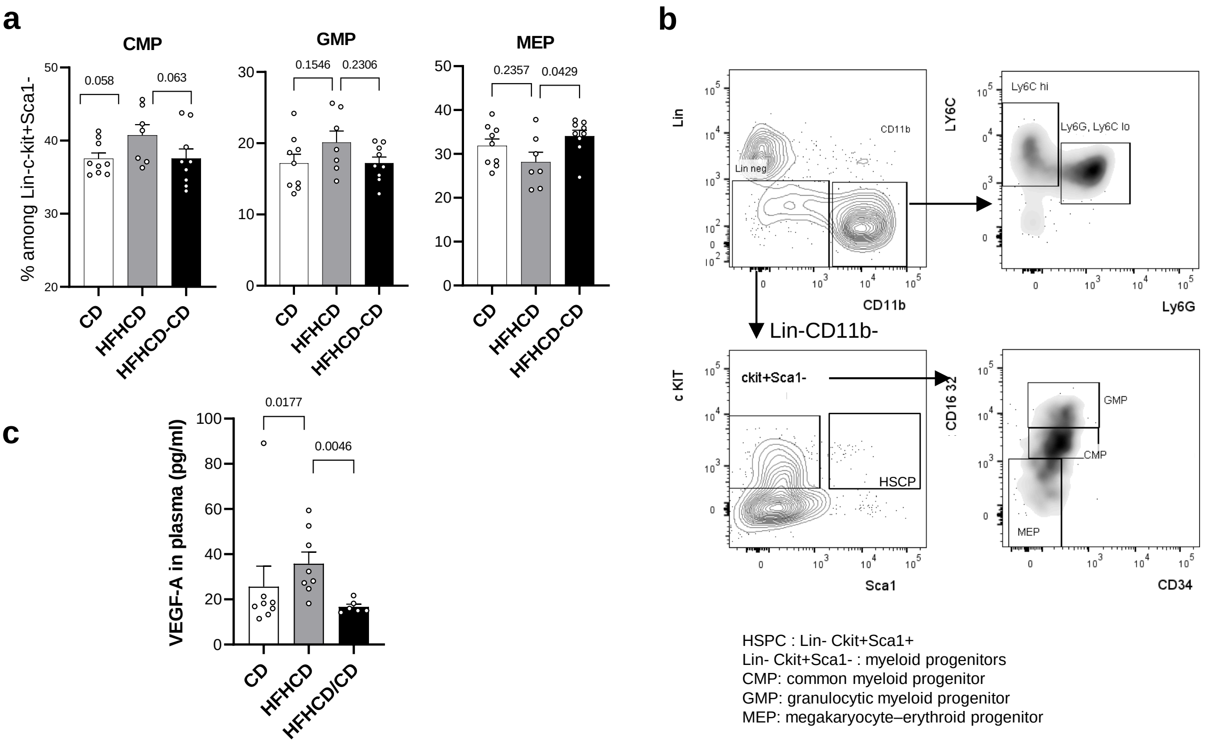

Supplementary Figure 8: Switching diet avoid the effect of HFHCD on tumor growth
C57BL/6J mice were fed for 2 weeks with CD or HFHCD, one group HFHCD was switched to CD the day of tumor graft. Mice were transplanted with B16-F10 cells (s.c) and sacrificed at day 12 post injection. **a)** Flow cytometry analysis of bone marrow myeloid progenitors (CMP: common myeloid progenitor; GMP: granulocytic myeloid progenitor; MEP: megakaryocyte–erythroid progenitor). (n=9 in CD group, n=7 in HFHCD group, n=9 in HFHCD-CD group). **b)** Gating stategy for analysis of bone marrow myeloid progenitors, monocytes and neutrophils. **c)** VEGF-A level in plasma was measured by bead-based multiplex immunoassay. (n=8 in CD group, n=8 in HFHCD group, n=6 in HFHCD-CD group). . Data are expressed as mean ± s.e.m., one-way ANOVA with Tukey's multiple comparison test. Source data are provided as a Source Data file.

Diet nutritional composition

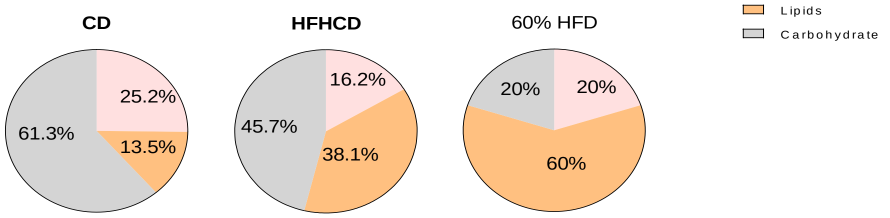

%Fatty acids	Chow Diet	HFHCD	60% HFD
Palmitic	0.76%	4.07%	7.93%
Palmitoleic	0.05%	0.06%	0.94%
Stearic	0.15%	5.22%	4.37%
Oleic	1%	5.23%	13.97%
Linoleic	2.5%	1.56%	4.64%
linolenic	0.28%	0.21%	0.49%
Myristic	-	0.02%	0.44%
Arachidic	-	0.16%	0.11%

**% cholesterol**	0%	1.25%	0%

Supplemental table 1
compositon (%) of fatty acids and cholesterol in CD, HFHCD and in 60% HFD
CD Safe, A03; HFHCD Ssniff Paigen mod., 15% cocoa butter and 1.25% cholesterol, Catalog No. E15106-347; 60%HFD Ssniff DIO-60 kJ% fat, Catalog No. E15742-347

Flow cytometry reagents and anti-mouse antibodies

	Manufactor	clone	Cat#	Dilution and amount per test
FC block anti CD16/32 purified	eBiosciences	93	14-0101-86	1/100=0,5ug
Live/dead aquablue	Life Technologies	-	L34957	0,5ul/test
Foxp3 / Transcription Factor Staining Buffer Set	eBiosciences	-	00-5523-00	Fix/Perm 1X : 100ul/test, Perm Buffer 1X : 200ul/test
CD45 AlexaFluo700	eBiosciences	30-F11	56-0451-82	1/200=0,05ug
CD45 BV605	BD Biosciences	30-F11	563053	1/200=0,05ug
CD11b APCefluo780	eBiosciences	M1/71	47-0112-82	1/400=0,025ug
CD11b PECY7	Biolegend	M1/71	101215	1/400=0,025ug
Ly6C PercpCy5.5	eBiosciences	HK4.4	45-5932-82	1/100=0,1ug
Ly6C BV421	BD Biosciences	AL-21	562727	1/200=0,05ug
Ly6G PE; AlexaFluo700	BD Biosciences	1A8	551461;561236	1/200=0,05ug
GR-1 Percp Cy5.5	Biolegend	RB6-8C	108428	1/100=0,1ug
F4/80 BV421	Biolegend	BM8	123131	1/75=0,13ug
F4/80 BUV395	BD Biosciences	T45-2342	565614	1/75=0,13ug
CD8a APC	eBiosciences	56-6.7	17-0081-83	1/400=0,025ug
CD8b BUV495	BD Biosciences	H35-17.2	741127	1/500=0,02ug
CD4 APC	eBiosciences	GK1.5	17-0041-82	1/400=0,025ug
CD4 Pacific Blue	Biolegend	RM4-5	100531	1/200=0,125ug
CD3e PercpCy5.5	eBiosciences	145-2C11	45-0031-82	1/100=0,1ug
CD3e BUV395	BD Biosciences	145-2C11	563565	1/100=0,1ug
CD25 BV711	Biolegend	PC61.5	102049	1/100=0,1ug
CD25 APCefluo780	eBiosciences	PC61.5	47-0251-82	1/100=0,1ug
FoxP3 PE	eBiosciences	FJK-16s	12-5773-82	1/100=0,1ug
PD1 FITC	eBiosciences	J43	11-9985-85	1/100=0,25ug
PD-L1 APC	Biolegend	10F.9G2	124311	1/100=0,1ug
NK1.1 APC CY7	Biolegend	PK136	108724	1/100=0,1ug
NK BV650	BD Biosciences	PK136	564143	1/100=0,1ug
CD115 BV711	Biolegend	AFS98	135515	1/100=0,1ug
CCR2 PE	Biolegend	QA18A56	160105	1/100=0,1ug
CX3CR1 PercpCy5.5	Biolegend	SA011F11	149009	1/100=0,1ug
CMH II APC	Biolegend	M5/114.15.2	107614	1/300=0,033ug
CD206 PECY7	Biolegend	C068C2	141720	1/50=0,2ug
Ki67 BV786	BD Biosciences	B56	563756	1ul/test
pro-IL1b FITC	eBiosciences	NJTEN3	11-7114-82	1/50=0,5ug
TNFa PECY7	eBiosciences	MP6-XT22	25-7321-82	1/100=0,1ug

IL10 APC	eBiosciences	JES5-16E3	17-7101-82	1/50=0,2ug
IL-17 PE	Biolegend	TC11-18H10.1	506904	1/100=0,1ug
IFNg AF488	Biolegend	XMG1.2	505813	1/100=0,25ug
Granzyme B AlexaFluo647	Biolegend	GB11	515406	0,1ug
CD44 AlexaFluo700	Biolegend	IM7	103026	1/100=0,25ug
CD62L PercpCy5.5	Biolegend	MEL-14	104432	1/100=0,1ug
CD2 biotin	Biolegend	RM2-5	100103	1/200=0,125ug
CD3e biotin	Biolegend	145-2C11	*100303*	1/200=0,125ug
CD4 biotin	Biolegend	GK1.5	100403	1/200=0,125ug
CD19 biotin	Biolegend	6D5	115504	1/200=0,125ug
CD8a biotin	Biolegend	56-6.7	100703	1/200=0,125ug
CD45R/B220 biotin	Biolegend	RA3-6B2	103203	1/200=0,125ug
Ter119 biotin	Biolegend	Ly-76	116203	1/200=0,125ug
Streptavidin FITC	Biolegend	-	405201	1/200=0,125ug
c-kit 4APC-Cy7	Biolegend	2B8	105825	1/100=0,1ug
Sca1 BV605	Biolegend	D7	108134	1/100=0,1ug
CD16/32 PE	Biolegend	93	101308	1/100=0,1ug
CD34 Percpcy5.5	Biolegend	HM34	128607	1/100=0,1ug
CD45.1 PE	Biolegend	A20	110708	1/100=0,1ug
CD45.2 AlexaFluo700	eBiosciences	104	56-0454-82	1/200=0,12ug
CXCR4 AF488	eBiosciences	2B11	53-9991-80	1/50=0,5ug
Streptavidin BV650	Biolegend	-	405231	1/100=0,05ug
BODIPY FL C16	Life Technologies	-	D3821	50ug i.p /mouse
2-NBDG	Life Technologies	-	N13195	200ug i.v /mouse

Immunohistochemistry reagent and antibodies

	Manufactor	clone	Cat#	
Mouse and Rabbit Specific HRP/DAB IHC Detection Kit - M	Abcam	-	ab236466	
Recombinant Anti-CD31 antibody	Abcam	EPR17259	ab182981	1/2000
Recombinant Anti-Ki67 antibody	Abcam	SP6	ab16667	1/200
Recombinant Anti-alpha smooth muscle Actin antibody	Abcam	EPR5368	ab124964	1/2000
Aqueous mounting medium	Abcam		ab64230	

Primer sequences

Name	Sequence	
IL-1β_F	TGGACCTTCCAGGATGAGGACA	
IL-1β_R	GTTCATCTCGGAGCCTGTAGTG	
VEGF-A_F	CTGCTGTAACGATGAAGCCCTG	
VEGF-A_R	GCTGTAGGAAGCTCATCTCTCC	
IL-6_F	TACCACTTCACAAGTCGGAGGC	
IL-6_R	CTGCAAGTGCATCATCGTTGTTC	
CXCL1_F	TCCAGAGCTTGAAGGTGTTGCC	
CXCL1_R	AACCAAGGGAGCTTCAGGGTCA	
CX3CL1_F	CAGTGGCTTTGCTCATCCGCTA	
CX3CL1_R	AGCCTGGTGATCCAGATGCTTC	
CX3CR1_F	GAGCATCACTGACATCTACCTCC	
CX3CR1_R	AGAAGGCAGTCGTGAGCTTGCA	
CCL2_F	GCTACAAGAGGATCACCAGCAG	
CCL2_R	GTCTGGACCCATTCCTTCTTGG	
CCR2_F	GCTGTGTTTGCCTCTCTACCAG	
CCR2_R	CAAGTAGAGGCAGGATCAGGCT	
CCL5_F	CCTGCTGCTTTGCCTACCTCTC	
CCL5_R	ACACACTTGGCGGTTCCTTCGA	
CCR5_F	GTCTACTTTCTCTTCTGGACTCC	
CCR5_R	CCAAGAGTCTCTGTTGCCTGCA	
CXCR4_F	GACTGGCATAGTCGGCAATGGA	
CXCR4_R	CAAAGAGGAGGTCAGCCACTGA	
GM-CSFR_F	CAGTTTGAGGTCCAGTGGCAGA	
GM-CSFR_R	CCAGTGCTTCATCCTCGTGTCG	
RPL13A_F	CTGCTCTCAAGGTTGTTCGGCT	
RPL13A_R	CCTTCCGTTTCTCCTCCAGAGT	

Supplemental table 2
Reagents and antibodies

Supplemental materiel

2’-deoxy-2’-[18F]fluoro-D-glucose (FDG) positron emission tomography–computed tomography (PET-CT) imaging

Prior to each imaging session, mice were fasted overnight with water ad libitum. Mice were anesthetized with isoflurane (IsoVet 100%; Centravet, France) in 100% oxygen (4% isoflurane for induction; 1–2% for maintenance). Mice were weighted, and placed on a heated plate (Minerve, France). Glycemia was measured in blood drawn from the caudal ventral artery using an Accu-Chek® Aviva Nano A (Accu-Chek, France). A customized catheter with a 29 G needle (Fischer Scientific, France) connected to a 5-cm polyethylene tubing (Tygon Microbore Tubing, Cole-Parmer0.020” × 0.060“OD; Fisher Scientific, France) was installed in the lateral tail vein of the mice. 10 MBq of FDG (Gluscan, Advanced Applied Applications, France) in 200 µl of saline solution were injected in the mice. The mice were then put back in their cages and left awake for 30 min. Mice were anesthetized again and installed in prone position in the imaging bed of the camera (NanoScan PET-CT, Mediso Medical Imaging Systems, Hungary). Respiration and body temperature were registered. Body temperature was maintained at 37°C and anesthesia was controlled on the breathing rate throughout the entire examination. CT scans were performed first using the following parameters: mode semi-circular, tension of 50 kV, 720 projections full scan, 300 ms per projection, binning 1:4. CT data were reconstructed using filtered back projection (filter : Cosine ; cutoff: 100%). List-mode PET data were collected between 45 and 60 min post injection of FDG, binned using a 5-ns time window, a 400- to 600-keV energy window, and a 1:5 coincidence mode. *In vivo* PET acquisitions were reconstructed using the Tera-Tomo reconstruction engine (3D-OSEM based manufactured customized algorithm, Mediso Medical Imaging Systems, Hungary) with expectation maximization iterations, scatter and attenuation corrections. Images were analyzed using the software PMOD (PMOD Technologies LLC, Switzerland). Standardized Volume of Interest (VOI) was drawn in each organ of interest and Standardized Uptake Values (SUV) were calculated by dividing the mean tissue radioactivity concentration by the whole body concentration of the injected radioactivity. The Peak value was calculated as the maximum average SUV within a 1-cm3 spherical volume of interest and the tumor volume was automatically segmented at 33% of this value. Total FDG uptake was estimated as the product from the volume by the mean uptake of the segmented region.

***In vivo* uptake of 2-NBDG and C16 BODIPY**

At day 12-13 after tumor graft, mice were fastened overnight. To test *in vivo* uptake of glucose, mice receive 200µg (iv) of the fluorescent glucose analogue 2-NBDG (2-[*N*-(7-nitrobenz-2-oxa-1, 3-diazol-4-yl) amino]-2-deoxyglucose) (Life Technologies) 15 min before sacrifice. To test *in vivo* uptake of lipid, mice were injected with 50µg of fluorescent palmitate Bodipy FL C16 (Life Technologies) 1h before sacrifice. Uptake among main immune cells and CD45 negative cells (mostly tumor cells) was analyzed by flow cytometry.

Bone marrow supernatant

Bone marrow supernatants were obtained from mice fed during 2 weeks with HFHCD or CD. The metaphysis of one end tibia was removed; the tibia was centrifugated 6000g 10 min at 4°C (‘open end tibia’ facing down). Then the bone marrow pellet was resuspended in 100 µl PBS-BSA 0.5%, mixed, then centrifugated down again. Collected supernatants were stored at -80°c for Luminex assay.

Bone marrow HSPC isolation

Bone marrow cells were flushed from femur and tibia of mice fed during 2 weeks with HFHCD or CD, **by** using a syringe 23 gauge needle filled with PBS. Cell suspension was prepared by passing through a 70 µm mesh nylon strainer. Then hematopoietic progenitors cells (HSPC) were isolated with EasySep™ Mouse Hematopoietic Progenitor Cell Isolation Kit (Stem Cell), and lysed in RLT buffer (Qiagen).
