# Supplementary material for: Mild dyslipidemia accelerates tumorigenesis through expansion of Ly6Chi monocytes and differentiation to pro-angiogenic myeloid cells
Source: Nat Commun. 2022 Sep 14;13:5399. doi: 10.1038/s41467-022-33034-0 (PMC9475043; doi:10.1038/s41467-022-33034-0)
Supplement: Supplementary file 2 — Reporting Summary [file 41467_2022_33034_MOESM2_ESM.pdf]

nature portfolio

Corresponding author(s): Stephane Potteaux

Last updated by author(s): Jul 22, 2022

Reporting Summary

Nature Portfolio wishes to improve the reproducibility of the work that we publish. This form provides structure for consistency and transparency in reporting. For further information on Nature Portfolio policies, see our Editorial Policies and the Editorial Policy Checklist.

Statistics

For all statistical analyses, confirm that the following items are present in the figure legend, table legend, main text, or Methods section.

n/a	Confirmed	
☐	☒	The exact sample size (*n*) for each experimental group/condition, given as a discrete number and unit of measurement
☐	☒	A statement on whether measurements were taken from distinct samples or whether the same sample was measured repeatedly
☐	☒	The statistical test(s) used AND whether they are one- or two-sided<br>*Only common tests should be described solely by name; describe more complex techniques in the Methods section.*
☒	☐	A description of all covariates tested
☒	☐	A description of any assumptions or corrections, such as tests of normality and adjustment for multiple comparisons
☐	☒	A full description of the statistical parameters including central tendency (e.g. means) or other basic estimates (e.g. regression coefficient) AND variation (e.g. standard deviation) or associated estimates of uncertainty (e.g. confidence intervals)
☐	☒	For null hypothesis testing, the test statistic (e.g. *F*, *t*, *r*) with confidence intervals, effect sizes, degrees of freedom and *P* value noted<br>*Give P values as exact values whenever suitable.*
☒	☐	For Bayesian analysis, information on the choice of priors and Markov chain Monte Carlo settings
☐	☒	For hierarchical and complex designs, identification of the appropriate level for tests and full reporting of outcomes
☒	☐	Estimates of effect sizes (e.g. Cohen's *d*, Pearson's *r*), indicating how they were calculated

Our web collection on statistics for biologists contains articles on many of the points above.

Software and code

Policy information about availability of computer code

Data collection	Flow cytometry data were collected on a Fortessa X20 and on a LSR Fortessa Analyser with BD DIVA software V8 (BD Biosciences) and analyzed with FlowJo software (V10).<br>Luminex assay datas were collected on Bioplex 200 manager software (Bio-Rad)<br>Vessel density was evaluated by laser-Doppler (Moor Instrument)<br>in vivo 18F FDG-PET was monitoring with NanoScan PET-CT, Mediso Medical Imaging Systems<br>for transcriptomic analysis :Affymetrix MouseGene2.0ST array at de GENOM'IC core facility (Cochin Institute)
Data analysis	Flow cytometry data were analysed on FlowJo V10 (BD)<br>Luminex assay data were analyzed on BioPlex manager software with 5-PL regression (Bio-Rad)<br>Statistical analysis were performed on prism V9 (Graph Pad)<br>Quantification of the vessel density was performed with MoorLDIReview V6.1 software<br>In vivo PET acquisitions images were analyzed using the software PMOD (PMOD Technologies LLC, Switzerland<br>for transcriptomic analysis :Affymetrix MouseGene2.0ST array at de GENOM'IC core facility (Cochin Institute). P-values were obtained by two-sided Student's t-test with group variance within limma package. Genes with p-value < 0.05 were used for enrichment pathway analysis with R library clusterProfiler with Gene Ontology knowledgebase<br>Immunohistochemistry stainings were quantified with ImageJ (FIJI)

For manuscripts utilizing custom algorithms or software that are central to the research but not yet described in published literature, software must be made available to editors and reviewers. We strongly encourage code deposition in a community repository (e.g. GitHub). See the Nature Portfolio guidelines for submitting code & software for further information.

Data

Policy information about availability of data

All manuscripts must include a data availability statement. This statement should provide the following information, where applicable:

- Accession codes, unique identifiers, or web links for publicly available datasets
- A description of any restrictions on data availability
- For clinical datasets or third party data, please ensure that the statement adheres to our policy

Source data are provided with this paper.
The transcriptomic and lipidomic data generated in this study have been deposited in the Open Science Framework database under the DOI 10.17605/OSF.IO/TE8S2 and accession code https://osf.io/te8s2/.

Field-specific reporting

Please select the one below that is the best fit for your research. If you are not sure, read the appropriate sections before making your selection.

☒ Life sciences ☐ Behavioural & social sciences ☐ Ecological, evolutionary & environmental sciences

For a reference copy of the document with all sections, see nature.com/documents/nr-reporting-summary-flat.pdf

Life sciences study design

All studies must disclose on these points even when the disclosure is negative.

Sample size	No sample size calculation was performed. Sample size was determined based on similar studies by the group of Pr Tartour and based on our experience (doi: 10.1038/ncomms15221). Experiments were designed to get at least 3 mice in each group. The value of n is indicated in the figure and legend.
Data exclusions	As requested by Reviewer 1, we have performed an outlier test (Grubbs's test) to remove possible outliers (Fig. 2D, 1 mouse in HFHCD group; Fig. 6D, 1 mouse in WT-CD group; Fig. 5C, 1 mouse in IL-1b+/+ CD group), and excluded tumors that did not developed well when this was related to technical reasons
Replication	experiment repetitions are reported in the figure legends. All experiments were reproduced to reliably support conclusions stated in the manuscript. All attempts at replication was successful
Randomization	The animals were grouped randomly and blindly to researchers. Mice were matched by age. For in vitro and ex vivo studies, cells were allocated to experimental group randomly.
Blinding	Diet administration was done by the animal-technicians who had no information about the expected outputs and the nature of the used diets. Whenever possible during the experiments, the investigators were blinded except when specific reagents or diets were used. Tumor size monitoring was done in the presence of two persons. With the consumption of fat diet, mice were eventually distinguishable from CD fed mice, which could make blinding opsolete after couple of weeks.

Reporting for specific materials, systems and methods

We require information from authors about some types of materials, experimental systems and methods used in many studies. Here, indicate whether each material, system or method listed is relevant to your study. If you are not sure if a list item applies to your research, read the appropriate section before selecting a response.

Materials & experimental systems

n/a	Involved in the study	
☐	☒	Antibodies
☐	☒	Eukaryotic cell lines
☒	☐	Palaeontology and archaeology
☐	☒	Animals and other organisms
☒	☐	Human research participants
☒	☐	Clinical data
☒	☐	Dual use research of concern

Methods

n/a	Involved in the study	
☒	☐	ChIP-seq
☐	☒	Flow cytometry
☒	☐	MRI-based neuroimaging

Antibodies

Antibodies used	Please see details in supplemental table 2.

Validation

All the antibodies are from commercial source and have been validated by the vendors and their validation data are available on the manufacturers' website. Control isotypes and secondary antibosy alone were used as control. No validation statements were done on the manufacturer's website
Bone marrow progenitors markers and gating stategy were based on Koelwyn GJ. Nat Med 2020.
in vivo uptake of 2-NBDG and Bodipy-FL-C16 experiments were based on Pacella I. PNAS 2018 with some modification in concentration of 2-NBDG more suitable for our tumor model.

Eukaryotic cell lines

Policy information about cell lines

Cell line source(s)

The B16-F10 was purchased from ATCC (CRL6475) and authentified by the manufactor (cytochrome oxidase 1 (CO1) barcoding assay). TC-1 cells (non commercial cell line) expressing the HPV16 E6-E7 proteins were obtained from the laboratory of T.C. Wu (Dept of Pathology, School of Medicine, Johns Hopkins University, Baltimore, MD).

Authentication

none of the cell lines were authenticated

Mycoplasma contamination

mycoplasma contamination was tested once per year by using PlasmoTest (Invivogen). All cell lines used in the manuscript were tested negative for mycoplasma contamination

Commonly misidentified lines
(See ICLAC register)

No commonly misidentified cell lines were used in the study.

Animals and other organisms

Policy information about studies involving animals; ARRIVE guidelines recommended for reporting animal research

Laboratory animals

All experiments were conducted according to European Community for experimental animal use guidelines (EC2010/63), and have been approved by the Ethical committee of University Paris Descartes (CEEA 34) and the French Ministry of Agriculture (APAFIS #17112- 2018040515553905 – V3). To investigate the effect of dyslipidemia on tumor development, we implanted the murine cancer lines in female C57BL/6J aged 8-10 weeks (Charles River). OT-1 mice (CD8 transgenic Tcr for ovalbumin 257-264) were also purchased from Charles River. IL-1β-/- mice were kindly given by Pr Yoichiro Iwakura (Tokyo University of Science, Japan). IL1R1 flox mice were given by Dr. Pinteaux. LysMCre-/-VEGFf/f (WT) and LysMCre+/-VEGFf/f (VEGF-A LysM) mice were provided by Dr Christian Stockmann. All mice were on C57BL/6J background.
Mouse breeding occurred in our animal facility in accordance with local recommendations. Control mice were matched with littermates of the appropriate, age, sex, and genetic background to account for any variation in data, when specified. Mice received a standard chow diet (Safe, A03), a pro-obese high fat diet (Ssniff DIO-60 kJ% fat, Catalog No. E15742-347) or pro-atherogenic high fat/high cholesterol diet (Ssniff Paigen mod., 15% cocoa butter and 1.25% cholesterol, Catalog No. E15106-347) (Supplemental Table 1). Mice were housed under a 12 h light-dark cycle with ad libitum access to food and water, 50–70% humidity, and 18–22 °C ambient temperature. Tumor size never reached the maximum allowable size of 20 mm in diameter. Mouse euthanasia was performed by cervical dislocation after anesthesia with 4% isoflurane (IsoVet 100%; Centravet, France) in 100% oxygen in an anesthetic chamber which was not prefilled to prevent distress. Plasma cholesterol was measured using a commercial kit (DiaSys Cholesterol FS*, Germany).

Wild animals

No wild animals were used in the study.

Field-collected samples

No field collected samples were used in the study

Ethics oversight

All experiments were conducted according to European Community for experimental animal use guidelines (EC2010/63), and have been approved by the Ethical committee of University Paris Descartes (CEEA 34) and the French Ministry of Agriculture (agreement 17112).

Note that full information on the approval of the study protocol must also be provided in the manuscript.

Flow Cytometry

Plots

Confirm that:

☒ The axis labels state the marker and fluorochrome used (e.g. CD4-FITC).

☒ The axis scales are clearly visible. Include numbers along axes only for bottom left plot of group (a 'group' is an analysis of identical markers).

☒ All plots are contour plots with outliers or pseudocolor plots.

☒ A numerical value for number of cells or percentage (with statistics) is provided.

Methodology

Sample preparation

Cell surface and intracellular staining were performed on single-cell suspensions. For intracellular staining, cell were fixed and permeabilized using the Foxp3/Transcriptional Factor Staining Buffer Set (Thermo Fisher).
Single-cell suspensions were prepared from tumor by mechanic dissociation in PBS-FBS 2% with GentleMacs dissociator

(Miltenyi Biotec), then crushing on 70um cell strainers. Single-cell suspensions were prepared from spleen were prepared by crushing on 40um cell strainers, red blood cells were lysed with osmotic lysis buffer for 2min (ACK).
Blood staining were performed on total blood after red blood cell lysis.
Cell surface staining were performed after Fc receptor blocking with anti CD16/32 (except for bone marrow progenitor staining). For intracellular staining, cell were first fixed and permeabilized using the Foxp3/Transcriptional Factor Staining Buffer Set (Thermo Fisher), before intracellular staining

Instrument

Fortessa X20 (BD) flow cytometer was used for analysis, and AriaII (BD) was used for cell sorting

Software

FACSDiva software (BD) and FlowJo v10 (BD)

Cell population abundance

at least 20 000 events in CD45+ live singulets cells gate were acquired for analysis.
For MDSC isolation purety was checked and cells numbers was ajusted before performing experiment
Total numbers were calculated by muliplying the total cells by percent cells within the gate

Gating strategy

cells were first selected on FSC-A and SSC-A on known size and granularity of leukocytes, doublets were excluded on FSC-A/FSC-H. CD11b+ cells were gated among CD45+ live cells, then sub-populations of myeloid cells were analyzed among CD11b+ on Ly6C, Ly6G and F4/80 expression.
For bone marrow progenitors, doublets were first excluded on FSC-A/FSC-H, then Lineage + and CD11b+ cells. Then among c-kit+Sca1+ cells, progenitors (CMP, GMP, MEP) were analyzed.
Positive and negative population were identified with isotype and FMO controls.
Please see gating stategy in supplemental Fig S1a and Fig S1b; Fig S2b and Fig S7b.

☒ Tick this box to confirm that a figure exemplifying the gating strategy is provided in the Supplementary Information.
