# Supplementary material for: Mild dyslipidemia accelerates tumorigenesis through expansion of Ly6Chi monocytes and differentiation to pro-angiogenic myeloid cells
Source: Nat Commun. 2022 Sep 14;13:5399. doi: 10.1038/s41467-022-33034-0 (PMC9475043; doi:10.1038/s41467-022-33034-0)
Supplement: Supplementary file 3 — Peer Review File [file 41467_2022_33034_MOESM3_ESM.pdf]

nature portfolio

# Peer Review File

## Mild dyslipidemia accelerates tumorigenesis through expansion of Ly6C$^{hi}$ monocytes and differentiation to pro-angiogenic myeloid cells

**Open Access** This file is licensed under a Creative Commons Attribution 4.0 International License, which permits use, sharing, adaptation, distribution and reproduction in any medium or format, as long as you give appropriate credit to the original author(s) and the source, provide a link to the Creative Commons license, and indicate if changes were made. In the cases where the authors are anonymous, such as is the case for the reports of anonymous peer reviewers, author attribution should be to 'Anonymous Referee' followed by a clear attribution to the source work. The images or other third party material in this file are included in the article's Creative Commons license, unless indicated otherwise in a credit line to the material. If material is not included in the article's Creative Commons license and your intended use is not permitted by statutory regulation or exceeds the permitted use, you will need to obtain permission directly from the copyright holder. To view a copy of this license, visit http://creativecommons.org/licenses/by/4.0/.

**REVIEWER COMMENTS</B>**

Reviewer #1 (Remarks to the Author); expert on high-fat diet, mouse models and immunology:

Manuscript NCOMMS-20-39668

In this manuscript Tran et al. address the role of low-grade inflammation caused by mild-dyslipidemia on tumor growth. In particular they show that feeding with high fat/high cholesterol (HFHCD) causes the increase of Ly-6C+ monocytes in the blood and amplifies myeloid cell recruitment in melanoma tumors. The HFHCD induces pro-angiogenic, pro-inflammatory and immunosuppressive phenotype in myeloid cells. The authors further assessed the role of Ly-6Chigh monocytes in tumor development. Depletion of monocytes limits immune cell recruitment and tumor growth, which is recapitulated by IL-1β deficiency and macrophage-specific VEGF-A deficiency. The findings may provide important information about the mechanisms that underlie tumor development in normal weight individuals with dyslipidemia, suggesting a potential benefit of targeting IL-1β and VEGF-A in cancer patients with dyslipidemia.

The understanding of how dyslipidemia affects cancer development and growth is a very interesting topic, since a large number of cancer patients present metabolic disorders at the moment of diagnosis or during treatment. However, the present study lack in a clear mechanism, and describes IL-1β or VEGF as major players in tumor associated macrophage-dependent tumor growth, which has been already reported in normal feeding scenarios in several types of cancer (PMID: 12598651, PMID: 23475218, PMID: 18509509). In general, the conclusions are not very strongly supported by the data and the authors should revise their raw data and perform better statistical analysis to detect outliers, that clearly affect the interpretation of the results in some cases. There is a clear strong variability in tumor weight in WT mice after HFHCD feeding along the study (Figure 1D, Figure 6B, and Figure 7C). The manuscript would also benefit from a better characterization of the changes in the immune cell compartment induced by short term HFHCD feeding.

Below there are some comments that may help to improve the manuscript:

-The authors show that tumor growth is boosted in mice fed with HFHCD for 15 days prior to tumor cell injection. The authors also show that the HFHCD causes an enrichment of circulating Ly-6Chigh monocytes that they claim support cancer cell proliferation and T cells immunosuppression. However, the manuscript lacks in data showing how cholesterol causes the enrichment of Ly-6Chigh monocytes. Can presence of cholesterol shift naïve monocytes to Ly-6C+ inflammatory monocytes in vitro? Does cholesterol uptake inhibition block Ly-6C+ monocytes expansion?

-Inflammatory monocytes are classically defined as Ly-6ChighCD11b+CD115+CCR2highCX3CR1low and patrolling monocytes are Ly-6Clow CD11b+CD115+CCR2lowCX3CR1high. It is not clear the gating strategy used in figure 1A and 1B, but presence of CCR2 marker is fundamental for migration to inflamed tissue or tumors and should be added to the gating strategy.

-In supplemental figure 1B, it seems that the effect of HFHCD feeding on TC1 cells growth is not that strong, showing only around 50 % of the mice with higher tumor size compare to CD feeding at the endpoint. This may suggest a direct effect of HFHCD in cancer cells proliferation additional to the monocytosis and increased recruitment of immune cells to the tumor, being then TC1 cells less sensitive than B16F10 cells.

-If dietary cholesterol is reduced by changing HFHCD feeding to CD at the moment of tumor cells injection or days after, do circulating Ly-6Chigh cells levels go back to those observed in mice fed with CD from the beginning of the experiment? Then, does tumor growth slow down? Is it possible that cholesterol rich diet cause irreversible epigenetic changes in circulating monocytes?

-The transcriptomic changes in the tumors may not be representative of tumor-associated macrophages transcriptome. Moreover, the representation of transcriptomic data is very poor, enriched pathways and most differentially expressed genes should be shown more clearly in the paper (including fold change and p-value), highlighting genes involved in angiogenesis, myeloid cells activation and immunosuppression.

-FDG uptake monitored by PET-CT scan shows increase total FDG uptake (but not mean intensity) in tumors of HFHCD-fed mice but the authors claim that it might be due to tumor size and not increased metabolic activity in tumor cells. Activated inflammatory macrophages show increased glycolytic activity and therefore they also uptake FDG similar to cancer cells. If there is an enrichment of monocytes/macrophages in the tumors (as shown in figure 3B) and we assume that the proliferative rate of cancer cells is higher (because the tumor growth is boosted) as suggested in figure 5D), after HFHCD feeding, then the FDG-PET-CT results are hard to reconcile.

-Figure 4B does not show increased levels of Ly-6Chigh in blood after HFHCD feeding in PBS liposomes treated group, which does not agree with data in figure 1A and the main hypothesis of the manuscript. What does it make Ly-6Chigh cells in HFHCD fed mice more susceptible to clodronate-mediated depletion if Ly-6Clow are thought to be more efficient phagocytes? There are many controversial data in the literature about the effect of clodronate liposomes and neutrophils depletion, it is shown in supplemental figure 3 that HFHCD increased neutrophils count in the blood and they increased even more after clodronate. Is there increased recruitment in the tumors? Some reports have suggested a prognostic value for neutrophil blood count, is it possible that neutrophils/monocytes ratio correlated with tumor size or growth?

-Data in figure 5 shows CD45+ cells but it is not clear whether Ly-6Chigh population is responsible for the increased cytokine and chemokine production in the tumor.

-A role for IL-1$\beta$ and VEGF-A in HFHCD induced tumor growth is evaluated but it is not fully clear the link between these two molecules rather than that both are mainly produced by myeloid cells in the tumor.

Reviewer #2 (Remarks to the Author); expert on inflammation and cancer:

In this paper, Tran et al. aim to assess the role of mild dyslipidemia in promoting a systemic inflammatory phenotype, accelerating tumor initiation in solid tumors. For this purpose, a mouse model of melanoma was used to show that short-term (two-week) feeding with a high fat/high cholesterol diet (HFHCD) leads to an IL-1β-dependent increase in circulating inflammatory Ly6Chi monocytes, associated with heightened infiltration of myeloid cells within the tumor and accelerated tumor initiation and outgrowth. In this HFHCD setting, myeloid cells are the primary source of proangiogenic VEGFA in the tumor microenvironment. Monocyte depletion reduced immune cell infiltration as well as tumor growth in mice fed with HFHCD, which was recapitulated by specific inhibition of VEGF-A in myeloid cells and in IL1β constitutive KO mice, suggesting that VEGFA secreted by myeloid cells is an important mediator of tumorigenesis in mice on a HFHCD.
The originality of this paper largely lies in investigating the impact of low-grade inflammation and silent dyslipidemia on tumorigenesis but also on the immune microenvironment, rather than in the context of symptomatic dyslipidemia in obese/metabolic syndrome settings, as has been previously done in different tumors models. However, the present study falls short on thoroughly analyzing the impact of HFHCD-driven mild dyslipidemia on the full immune landscape, and the relevance of this model used to generate low-grade inflammation and subclinical dyslipidemia is poorly defined. Moreover, the lack of mechanistic insights into how mild dyslipidemia functionally regulates systemic and local inflammation limits the originality of this study, especially in light of the fact that the majority of the findings of this paper pertaining to IL1B and VEGFA roles in tumorigenesis are already known in different tumor types and context.

General/major points:

1) The authors have not clearly defined the “low-grade inflammation” associated with their mouse model. A thorough analysis of the leukocyte blood content is lacking, as the authors only show a quantification of circulating Ly6Chi and Ly6Clo monocytes (and later mention a neutrophilia in Supplementary figure 3). A comprehensive analysis of all leukocyte populations is needed to characterize “low-grade” systemic inflammation, both in control mice and tumor-bearing mice, to confirm that it differs from inflammation induced by classic high fat diets in mouse models of obesity or metabolic syndromes.

2) On a similar note, the choice of melanoma as a model system is not obvious, and should be justified.
3) To establish the originality of their model of silent dyslipidemia in a non-obese setting, the study should present a comprehensive analysis of the dyslipidemic state (blood cholesterol but also lipid accumulation in tumor tissue) as well as mouse weights, and compare this to classic high fat diet and obesity/metabolic syndrome mouse models (e.g. Song et al Oncotarget 2017, PMID: 28410190). It is not clear how the HFHCD used in this paper is different, neither are the key cell types responding to this diet.
4) No proper justification has been given regarding the choice of studying silent dyslipidemia, e.g. do cases of silent dyslipidemia have increased tumor incidence or a shorter survival when diagnosed with cancer? Further correlation should be done using datasets of clinical samples of lean/overweight/obese cancer patients to ascertain the relevance of studying mild dyslipidemia, especially in melanoma- could this be associated with differences in immune checkpoint blockade response for instance.
5) While the authors have quantified the different immune cell populations infiltrating the tumor (Figure 3), additional characterization of the immune microenvironment should be addressed. What is the functional role of these immune cells, e.g. do macrophages display a more immunosuppressive phenotype compared to CD?
6) The RNAseq data set is a valuable resource of information which is not optimally used in this study (Figure 2). Not only does it contain information regarding immune activation, it also contains information about the activity of metabolic pathways. It will be important to show how the diet effects the metabolism of the tumor cells. Do they rely more on lipid-metabolism? How does this correlate with the corresponding immune response? Linking changing metabolic profile of the tumor in response to the diet to the immune response is needed to provide valuable insights regarding the underlying mechanism of the effect of mild dyslipidemia.
7) The choice of using IL-1b KO mice to is disputable. Indeed, Il-1b is produced by many immune and non-immune cells and has been broadly involved in a large number of inflammatory responses (Dinarello Mol Med 2014, PMID: 25549233). The authors suggest that it is IL-1b secreted by immune cells that controls the inflammation observed in their HFHCD model, but there is no data showing the potential cellular source of IL-1b; use of a conditional KO model where IL-1b is only suppressed in myeloid cells would be necessary to support that statement. Furthermore, IL-1b is well known to drive many inflammatory responses, including cancer-associated systemic inflammation and infiltration of immune cells in tumor tissues (Wellenstein et al Nature 2019 PMID: 31367040, Incio et al Cancer Discov. 2016, PMID: 27246539, Catstano et al Nat Cell Biol 2018 PMID: 30154549). It was therefore expected that the inflammatory response caused by HFHCD would be impacted by full loss of IL-1b, and further studies to establish a mechanistic link between pro-atherogenic diet and IL-1b-mediated monocytosis would be required to make this finding novel.
8) The authors chose to use clodronate liposomes to deplete myeloid cells, which has a few caveats including lack of specificity. The use of transgenic mouse models of inducible monocyte/macrophage depletion (Kanter et al Proc Natl Acad Sci U S A. 2012 PMID: 22308341, Goren Am J Pathol 2009 PMID: 19528348) or specific targeting of Ly6Chi monocytes by antibody-mediated depletion (Mack et al J Immunol 2001 PMID: 11254730) would be more appropriate to answer a specific question about the role of increased Ly6Chi monocytes in tumor growth acceleration. Also, the specific depletion of Ly6C low monocytes using this technic is surprising in light of these mentioned studies, the authors should

elaborate on these results.
9) The molecular mechanisms by which mild dyslipidemia affects monocytosis is not addressed in this study- is this related to systemic regulation of monocytic precursors' differentiation or does this regulation take place in the bone marrow? These are important aspects that should be experimentally addressed in this study.
10) Little to no analyses of spatial distribution of immune cells in these tumors is presented- this should be included to determine whether niche effects occur related to hypoxia, vascularization etc..in the context of HFHCD.

Specific comments:

11) Fig 1A: is it a percentage of CD45+ cells? Or total leukocytes?
12) Fig 1E: this data is somewhat unconvincing, it does not show a correlation
13) Fig 1D: PET-scan is not an appropriate method to measure tumor metabolism in this context, as this will mostly reflect tumor size. Further metabolic analyses should be performed.
14) Supp fig 2A: with this method, could they also label monocytes directly in the tumor and not just circulating monocytes?
15) Line 382: the authors conclude on increased monocyte infiltration of the tumor in HFHCD mice, however this is not clearly supported by the data shown in Supp fig 2B (small number of biological replicates, difference not statistically significant). This experiment should be repeated to strengthen the conclusion.
16) Fig 3A: this graph represents a count of CD45+ cells, however the increase observed could simply be due to a larger tumor size. This data needs to be normalized to tumor size (e.g. # CD45+ cells per 100g tumor)
17) Fig 3D: it seems like the figure legend is inaccurate. The colors on the tSNE plot represent different cell populations, not different expression levels.
18) Fig 4: clodronate liposome injections are not described in the method section, nor referenced
19) Fig 4B and C: why did the authors perform a Mann-Whitney test instead of ANOVA?
20) Fig 4D: matching data from the control CD group should be represented in this figure
21) Fig 5: why have the authors performed these assays on the whole CD45+ population? It would be interesting to see this data for more specific subsets, particularly on LyChi monocytes.
22) Fig 5A: are there any differences between CD and HFHCD without LPS and IFNgamma stimulation (basal levels)? Why use splenocytes and not monocyte-derived cells for example?
23) Fig 5B: There is a large difference in the number of data points for IL-1b KO compared to WT mice, which might cause the difference to be not significant for IL1b KO but significant for WT. A repeat experiment would be needed to have comparable group sizes.
24) Fig 6C: Il-1b is not just dispensable for MSDC activity; it looks like there is a higher suppression in Il-1b KO mice, suggesting that Il-1b reduces MSDC immunosuppressive activity.
25) Fig 5D: it would be interesting to see the matching data for mice on control diet.
26) Fig 7D: it is not specified whether the data shown comes from mice on CD or HFHCD
27) Supp fig 4c and d: do the authors have an explanation for the decrease in %CD11b+ in blood and %MDSC in tumor in HFD mice compared to CD, when previous figures show increased number of

CD11b+ cells in blood and accumulation of MSDC in tumor? Is it a % of CD45+ cells that is shown in this supplementary figure? In supp fig 4c, is there also no change if we look more specifically at 11b+ Ly6Chi subset in blood?
28) Fig 7A: The authors should indicate which dots belong to the CD or the HFHCD?
29) Fig 7F: can the authors develop on the meaning of ICAM-1 increase in total tumor?

Reviewer #3 (Remarks to the Author); expert on angiogenesis:

The link between high-fat diet and cancer progression, has been previously studied. There are several mechanisms have been proposed including the secretion of different fatty acids to promote resistance of tumor cells and their increased stemness. Immune cells, mesenchymal stem cells and even adipocytes have been demonstrated to affect tumor progression. In this study the authors focused on an in-depth mechanisms of tumor progression in mice fed with high-fat diet involving myeloid derived suppressor cells (MDSCs), which has been previously demonstrated as well. However, the involvement of IL1b and VEGF in this process has not been studied before.
The authors demonstrated that high-fat diet promotes the infiltration of MDSCs to tumors. In turn they secrete VEGFA and IL1b which contribute to tumor progression. The study uses different tools, mostly in vivo tools, to evaluate the immune cell composition and the effects of immune cells on tumor growth, cytokine secretion among other parameters.
The reviewer feels that this manuscript does not fit this journal for the following reasons:
First, the novelty of the study is questionable, as many additional studies described the basic phenomenon mentioned in this study (Clements 2018 PMID 29345342).
Second, there are some sophisticated methods that should have been used in order to study the immune composition in the tumor and the involvement of specific MDSC population which promotes tumor growth, e.g., adoptive transfer experiments of myeloid cells from mice fed with high fat diet compared to control mice.
Third, several possible mechanisms involved microbiome, angiogenesis per se, and other direct tumor activities have not been evaluated.
Fourth, the use of primary tumors until they reach 200mm3 raises many questions. Why the authors terminate the experiment in this early phase?
Fifth, the lack of data on metastasis is also surprising especially when studying IL1b and angiogenesis both of which are known to promote metastasis, and the use of B16F10 melanoma promotes pulmonary metastasis.
Sixth, some data of IL1b does not fit with previous published studies by Apte’s group, one of the major expert in the field. The authors did not find it important to address these discrepancies, e.g., IL1b KO mice in B16 melanoma tumors.
Seventh, the presentation of the results is no consistent. For example, the number of mice per each immune cell evaluation by flow cytometry raises question (n=4-5 mice per group) when the number of mice in the tumor growth studies was above 10 per group.
Based on all of these general comments, this reviewer feels that the manuscript is not suitable for

publication in this journal.
Below, the reviewer included additional specific comments for the benefit of the authors.
1. I am not sure that the style of the abstract is acceptable. This reviewer suggests that the style of the abstract will be more conservative.
2. OT mice are not defined in methods
3. Many abbreviations throughout the text are not defined first time mentioned.
4. In many places the Greek alphabet letters e.g., beta and gamma are written as b and g. This should be corrected.
5. As the authors probably aware of, MDSC is a heterogenous population including both monocytic and granulocytic phenotypes with sometimes distinct function in tumors. Can the authors comment on which MDSC populations. This should be define already in the intro and method.
6. Studies have already demonstrated that MDSCs promote angiogenesis via VEGF. There are studies published a decade ago, showing that VEGF expressed explicitly on myeloid cells, support angiogenesis. This is not a novel part in the study.
7. In the era of microbiome, one would ask whether the effect seen on tumor growth is associated with changes in the gut flor, which then affect the cellular composition of immune cells. While addressing this question is not in the scope of this manuscript, while still can be a mechanism, it should be mentioned at least in the discussion. In addition, an adoptive transfer of MDSC from mice fed with high-fat diet should be given to mice fed on normal diet in order to evaluate whether the effect on tumor growth disappears. In addition, a reverse experiment of adoptive transfer from mice on normal diet to mice with high-fat diet should change the growth of tumors. These studies are critical to evaluate the impact of MDSC on tumor growth in the context of high fat diet.
8. The metabolic reprogramming and hypoxia described are not shown in the transcriptomic analysis. how come?
9. It is not clear to this reviewer how tumor size of 100-200mm3 weigh 1000mg. For tumors to reach 1000mg they should be in a size of at least 1000-1500mm3. Something is wrong here.
10. Why the authors did not let the tumor growth much longer to see whether the differences in tumor growth are real? It seems unreasonable to terminate the experiment in 200mm3.
11. Why VEGF was tested only on 4 mice instead of 12 shown in the tumor growth curves? VEGF should be validated in all other tumors and not only the ones shown in the transcriptomic data.
12. The tSNE plot (figure 3D) is redundant to the results provided in Figure 3BC and can be included in supplemental data.
13. The B16F10 tumors are metastatic. The fact that the authors have seen an increase in several types of myeloid cells, including monocytic MDSCs suggests that the effect is not only in the primary tumor but also at the metastatic sites. What was the condition of the lungs in mice fed with high fat diet compared to normal diet.
14. Choldronate liposomes are not specific, and deplete several types of phagocytic cells including some macrophages, alveolar macrophages, neutrophils and others. A better approach should be carried out especially when macrophages and other CD45 monocytic cells enriched in the tumors of high fat diet mice compared with normal diet mice. In addition, the authors did not indicate the mortality rate of the mice administered with chlodronate injections, nor did they mention it in methods.
15. The authors focused on the stroma of the tumor to recruit myeloid cells. However, they failed to

show whether tumor cells secrete similar factors, therefore indicating that the effect is more general in the tumor microenvironment.

16. The results with IL1b KO (Figure 6A) do not fit with previously published studies. IL1bKO mice on their own display reduced tumor growth as tested in B16 melanoma. This has not been seen in the authors' data, therefore the reviewer wonder whether IL1b is indeed a factor involved in the data presented. Thus, the fact that the authors demonstrated reduced tumor growth in IL1b KO mice is misleading and the conclusions do not support the results as well as do not fit with the literature.

Author responses to review comments on Nature Communications manuscript NCOMMS-20-39668

**Mild dyslipidemia accelerates tumor growth in mice through expansion of Ly6C$^{hi}$ monocytes and acquisition of a myeloid cell-derived VEGF-A signature.**

We would like to thank the reviewers for their time and effort in reviewing our manuscript. Your comments, suggestions, and criticisms greatly helped us to improve the quality of this manuscript. We have extensively reformatted the manuscript to take into account your inputs and the requirements of Nature Communications,. We hope that the revised manuscript satisfies the standards required to warrant publication of our research in Nature Communications. Below is a point-by-point response to your comments.

**Reviewer # 1**

**Reviewer:** In this manuscript Tran et al. address the role of low-grade inflammation caused by mild-dyslipidemia on tumor growth. In particular they show that feeding with high fat/high cholesterol (HFHCD) causes the increase of Ly-6C+ monocytes in the blood and amplifies myeloid cell recruitment in melanoma tumors. The HFHCD induces pro-angiogenic, pro-inflammatory and immunosuppressive phenotype in myeloid cells. The authors further assessed the role of Ly-6Chigh monocytes in tumor development. Depletion of monocytes limits immune cell recruitment and tumor growth, which is recapitulated by IL-1β deficiency and macrophage-specific VEGF-A deficiency. The findings may provide important information about the mechanisms that underlie tumor development in normal weight individuals with dyslipidemia, suggesting a potential benefit of targeting IL-1β and VEGF-A in cancer patients with dyslipidemia.

The understanding of how dyslipidemia affects cancer development and growth is a very interesting topic, since a large number of cancer patients present metabolic disorders at the moment of diagnosis or during treatment. However, the present study lack in a clear mechanism, and describes IL-1β or VEGF as major players in tumor associated macrophage-dependent tumor growth, which has been already reported in normal feeding scenarios in several types of cancer (PMID: 12598651, PMID: 23475218, PMID: 18509509). In general, the conclusions are not very strongly supported by the data and the authors should revise their raw data and perform better statistical analysis to detect outliers,

that clearly affect the interpretation of the results in some cases. There is a clear strong variability in tumor weight in WT mice after HFHCD feeding along the study (Figure 1D, Figure 6B, and Figure 7C).

The manuscript would also benefit from a better characterization of the changes in the immune cell compartment induced by short term HFHCD feeding.

**Response:** We sincerely thank the Reviewer for her/his valuable comments, which improve the quality of our study.
We characterized in Fig. 1 and Supplemental Fig.1 the changes in the immune cell compartment and activation state induced by short term HFHCD feeding, and compared it to short term 60% HFD (obesogenic diet). To our knowledge, this has never been done before and should be instructive for future research. We have found that HFHCD induced a specific change in immune cell distribution and activation that is clearly different from the HFD (60% FAT) and had never been tested in tumorigenesis before.
We agree that we very often have quite strong variability in tumor weights. We have now performed outlier test (Grubbs's test) to remove possible outliers (Fig. 2D, 1 mouse in HFHCD group; Fig. 6D, 1 mouse in WT-CD group; Fig. 5C, 1 mouse in IL-1$\beta^{+/+}$ CD group), and excluded tumors that did not developed well as we think this was related to technical reasons (Suppl Fig. 2a, d9/12 group HFHCD). These modifications have not changed our interpretation of the results.

**Reviewer:** Below there are some comments that may help to improve the manuscript:

-The authors show that tumor growth is boosted in mice fed with HFHCD for 15 days prior to tumor cell injection. The authors also show that the HFHCD causes an enrichment of circulating Ly-6Chigh monocytes that they claim support cancer cell proliferation and T cells immunosuppression. However, the manuscript lacks in data showing how cholesterol causes the enrichment of Ly-6Chigh monocytes. Can presence of cholesterol shift naïve monocytes to Ly-6C+ inflammatory monocytes *in vitro*? Does cholesterol uptake inhibition block Ly-6C+ monocytes expansion?

**Response:** Data from the literature have shown that high cholesterol and fatty acid levels increase monocyte number in circulation by activating hematopoietic stem and progenitor cell (HSPC) proliferation, making more monocytes available for recruitment (Murphy AJ et al, J. Clin. Invest. 2011, PMID: 21968112; Yvan-Charvet L et al, Science 2010, PMID:20488992, Pernes G et al, Clin Transl Immunology, 2019 PMID 31890207). In mouse models of atherosclerosis, we and others have demonstrated that HFHCD increases the production of Ly6C$^{hi}$ monocytes (Baragetti A et al, Immunometabolism 2021, PMID 33859831; Combadière C et al, Circulation 2008, PMID 18347211)
In our current model, we demonstrate for the first time the HFHCD rapidly increases the production of Ly6C$^{hi}$ monocytes in wildtype mice. We have now added results showing that Common Myeloid Progenitors and Ly6C$^{hi}$ monocytes were increased in the bone marrow of tumor-bearing mice under HFHCD (Fig.7a).
We also observed progenitor cell increase in the bone marrow of mice without tumors (data below are not presented in the manuscript in order to avoid making the text too confusing) :

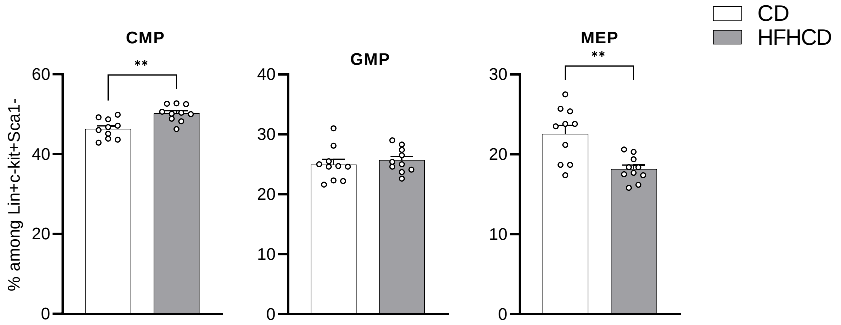


**Fig 1: HFHCD induces hematopoietic progenitor cell production in the bone marrow**. Flow cytometry analysis of bone marrow myeloid progenitors in mice under CD or HFHCD for 2 weeks (CMP: common myeloid progenitor; GMP: granulocytic myeloid progenitor; MEP: megakaryocyte–erythroid progenitor).

Interestingly, interruption of the HFHCD reduced Ly6C$^{hi}$ monocytes production in the bone marrow, showing that normalization of cholesterol levels regulate monocyte levels. We also performed

additional experiments where we purified Ly6C$^{hi}$ monocytes from the bone marrow (Miltenyi kit) of CD and HFHCD-fed mice and *in vitro* stimulated them with a combination of 3 lipids that are found in the diet, or with cholesterol and compared it to LPS stimulation. LPS induced significant increase in CCR2, IL-1β and IL-6. In contrast, lipid or cholesterol addition did not change those gene expressions. In conclusion, we did not find that cholesterol of free fatty acids shifted naïve monocytes to Ly-6C$^{+}$ inflammatory monocytes *in vitro*. We thought not to include those data into the manuscript.

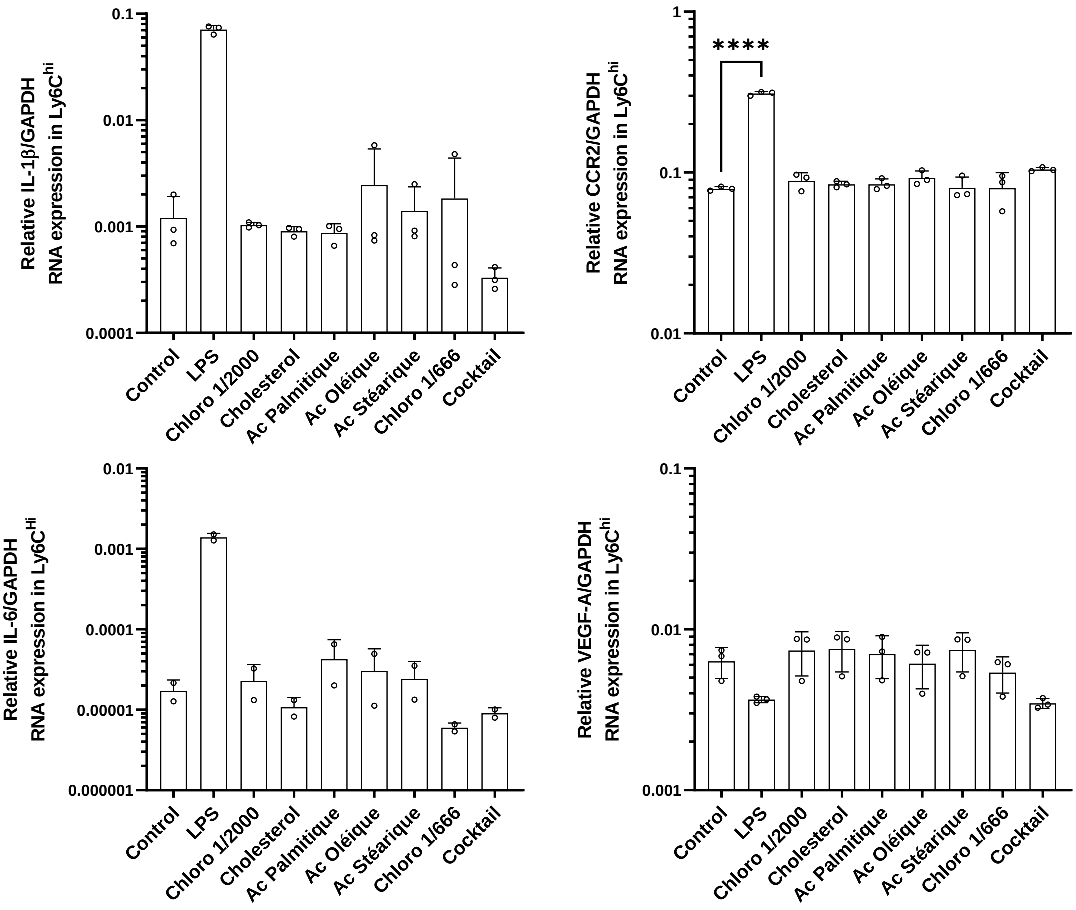


**Fig.2: Cholesterol or fatty acids do not activate monocytes *in vitro*.** Ly6Chi monocytes were isolated from the bone marrow of WT mice and incubated for 24 hours with the following agents: cholesterol (50mg/ml) or control (Chloroform 1/2000), oleic, palmitic, stearic acid or with a mix of oleic, palmitic and stearic acid (50mM each) or control (Chloroform 1/666)

**Reviewer:**-Inflammatory monocytes are classically defined as Ly-6ChighCD11b+CD115+CCR2highCX3CR1low and patrolling monocytes are Ly-6Clow CD11b+CD115+CCR2lowCX3CR1high. It is not clear the gating strategy used in figure 1A and 1B, but presence of CCR2 marker is fundamental for migration to inflamed tissue or tumors and should be added to the gating strategy.

**Response:** We thank the reviewer for this useful comment. We have now added our gating strategy in blood, spleen and tumor including CCR2 staining (Supplementary Fig.1 and Supplementary Fig.2b)

**Reviewer:** -In supplemental figure 1B, it seems that the effect of HFHCD feeding on TC1 cells growth is not that strong, showing only around 50 % of the mice with higher tumor size compare to CD feeding at the endpoint. This may suggest a direct effect of HFHCD in cancer cells proliferation additional to the monocytosis and increased recruitment of immune cells to the tumor, being then TC1 cells less sensitive than B16F10 cells.

**Response:** We fully agree with the Reviewer's comment, which led us to test this important question. We have tested the proliferation of tumor cells in response to cholesterol, fatty acids (identified in the

HFHCD and in HFHCD tumors), and in response to tumor explant supernatants. We have also quantified cell proliferation by Ki67 staining in sections of paraffin-embedded tumors. We specifically quantified Ki67 in tumoral zones (defined after HES staining, under the control of an anatomopathologist, Dr E. Brabencova, added as co-author of the paper), and found an increased rate of proliferation in the HFHCD group. Those data are shown in Fig.3 e, f and g. We found that fatty acids, consisting of a pool of oleic, palmitic and stearic acids, induced B16-F10 proliferation in comparison to stimulation with control vehicle (chloroform), *in vitro*. These data suggest a direct effect of HFHCD in cancer cell proliferation, which most likely parallel myeloid cell contribution to tumor growth exacerbation. We thank the Reviewer for this helpful comment.

**Reviewer:** -If dietary cholesterol is reduced by changing HFHCD feeding to CD at the moment of tumor cells injection or days after, do circulating Ly-6Chigh cells levels go back to those observed in mice fed with CD from the beginning of the experiment? Then, does tumor growth slow down? Is it possible that cholesterol rich diet cause irreversible epigenetic changes in circulating monocytes?

**Response:** We performed a new experiment to address the Reviewer's comment (Fig. 7 and Supplementary Fig. 7). We injected B16-F10 cells to 3 groups of mice, one being transitioned from a HFHCD to a CD (HFHCD-CD) at the time of cell injection. As expected, cholesterol levels were normalized in the HFHCD-CD. We found that tumor growth slowed down to the CD group level. Medullar Ly6C$^{hi}$ monocyte level decreased as well. Common myeloid progenitor cells and granulocytic myeloid progenitors of HFHCD-CD reached CD group level. Circulating Ly6C$^{hi}$ monocytes remained elevated. Similar counterintuitive observations were made in other models where monocytes control the disease progression. For instance, we have previously published that cholesterol normalization in ApoE$^{-/-}$ mice decreased atherosclerosis plaque size despite unaltered monocyte numbers in circulation (Potteaux et al, JCI 2011 PMID 21505265). More recently, HFD discontinuation in obese mice efficiently induced weight loss despite moderate effect on monocyte numbers in circulation (Liu et al Mol Immunol 2020, PMID 31778913)

**Reviewer:** -The transcriptomic changes in the tumors may not be representative of tumor-associated macrophages transcriptome. Moreover, the representation of transcriptomic data is very poor, enriched pathways and most differentially expressed genes should be shown more clearly in the paper (including fold change and p-value), highlighting genes involved in angiogenesis, myeloid cells activation and immunosuppression.

**Response:** We agree that the transcriptomic changes in the tumors are not representative of tumor-associated macrophage transcriptome and that representation was very poor in the first version of our manuscript. We apologize for that. We changed the data representation by showing the heatmap of the top 100 genes differentially expressed between the CD and HFHCD group with the gene names (Supplementary Fig. 5). We also included in Fig.6a a Volcano plot of all expressed genes, with *Vegfa* and differentially expressed genes from significantly enriched pathway highlighting. Genes differentially expressed in tumor under HFHCD together with fold changes are listed in Supplemental table 3. We found interesting to mention that enriched pathways include "negative regulation of myeloid leukocyte differentiation" in the HFHCD group, despite increased accumulation of leukocytes. This is in line with our new data showing, by luminex analysis, that MDSCs from HFHCD tumors secrete more VEGF-A but no more IL-1β and TNF-α (Supplementary Fig. 6a). We specified in the text that "Interestingly, despite the use of tumor bulk transcriptomes, "regulation of myeloid leukocyte differentiation" and "negative regulation of myeloid leukocyte differentiation" pathways were enriched ($p = 0.013$ and 0.015 respectively), as well as several metabolic pathways …". The text has been modified in consequences (page 9 line 24 and 10).

**Reviewer:** -FDG uptake monitored by PET-CT scan shows increase total FDG uptake (but not mean intensity) in tumors of HFHCD-fed mice but the authors claim that it might be due to tumor size and not increased metabolic activity in tumor cells. Activated inflammatory macrophages show increased glycolytic activity and therefore they also uptake FDG similar to cancer cells. If there is an enrichment of monocytes/macrophages in the tumors (as shown in figure 3B) and we assume that the proliferative rate of cancer cells is higher (because the tumor growth is boosted) as suggested in figure 5D), after HFHCD feeding, then the FDG-PET-CT results are hard to reconcile.

**Response:** In order to gain more information on the possible metabolic changes in intra-tumoral leukocytes and tumor cells, we performed new experiments where we monitored glucose and lipid

uptakes *in vivo* (Fig.3). We injected specific probes to mice under CD or HFHCD and quantified probe uptake, *in vivo*, in cells within the tumor by flow cytometry. We found that glucose and lipid combustion differed between cellular subsets in tumors but the pattern was not modulated by the diet. This agrees with our PET-Scan results (Supplementary Fig. 3). Nevertheless, we found that fatty acid composition in tumors differed between the 2 groups. We observed increased proportion of mono-unsaturated fatty acids and a higher omega-6/omega-3 ratio in the HFHCD tumors (Fig. 3). We found that the proliferation rate of B16-F10 cells was increased *in vitro* in response to the mix of fatty acids and in response to tumor explant supernatant. *In vivo*, as mentioned earlier, we found increased levels of Ki67 staining. Altogether, HFHCD does not affect the metabolism of cells in the tumor microenvironment, but rather modulates tumor cell lipogenesis, which probably additionally support tumor cell survival and proliferation.

**Reviewer:** -Figure 4B does not show increased levels of Ly-6Chigh in blood after HFHCD feeding in PBS liposomes treated group, which does not agree with data in figure 1A and the main hypothesis of the manuscript. What does it make Ly-6Chigh cells in HFHCD fed mice more susceptible to clodronate-mediated depletion if Ly-6Clow are thought to be more efficient phagocytes?

**Reviewer:** There are many controversial data in the literature about the effect of clodronate liposomes and neutrophils depletion, it is shown in supplemental figure 3 that HFHCD increased neutrophils count in the blood and they increased even more after clodronate. Is there increased recruitment in the tumors?

**Response:** In HFHCD group, we think that Ly-6C$^{hi}$ monocytes were more susceptible to clodronate depletion because they were more numerous than Ly-6C$^{lo}$ monocytes. It might only be the result of competition between the 2 subsets. Nevertheless, we agree that clodronate liposome depletion is not a very elegant way to address the role of myeloid cells in tumor development. We fully accept and learn from this criticism. We then decided to perform more elegant experiments and to remove the clodronate liposome depletion from the manuscript. Instead, we showed the direct implication of myeloid cells in tumor exacerbation under HFHCD by blocking myeloid cell accumulation through various approaches. We treated mice with an anti-CD115 antibody or with pharmacologic inhibitory agents. Both approaches limited tumor growth in the HFHCD. Similar conclusions were found in Il-1β$^{-/-}$ mice, where monocyte expansion is abrogated under HFHCD. Those data are shown in Fig.5.

**Reviewer:** Some reports have suggested a prognostic value for neutrophil blood count, is it possible that neutrophils/monocytes ratio correlated with tumor size or growth?

**Response:** In contrast to Ly6C$^{hi}$ monocytes, neutrophil blood count is not modulated by the HFHCD (Fig. 1). Although we found that monocytes directly contribute to tumor growth, we now think that monocyte blood count alone cannot predict tumor size. Indeed, our adoptive transfer experiments (Fig. 4c, d) show that monocyte migration is increased upon HFHCD. Furthermore, as suggested by the Reviewer, we found that tumor cell proliferation is increased in condition of HFHCD, which most likely contribute to tumor growth as well.

**Reviewer:** -Data in figure 5 shows CD45+ cells but it is not clear whether Ly-6Chigh population is responsible for the increased cytokine and chemokine production in the tumor.

**Response:** There was a mistake in the first version of the manuscript, we apologize about that. Luminex was performed on total B16-F10 tumor lysates and not on CD45+ cells. Those data are not correctly identified in Fig. 4a. Due to technical limitation, we were not able to specifically isolate Ly6C$^{hi}$ cells for Luminex experiments. Nevertheless, we have now provided new Luminex data on the supernatant of MDSCs (which contain Ly6C$^{hi}$ cells) isolated from tumors of the 2 groups (Fig. 6b and Supplementary Fig. 6a). Interestingly, we found that MDSCs from HFHCD group produce higher amount of VEGF-A than MDSCs from CD group, but they do not produce more inflammatory cytokines.

**Reviewer:** -A role for IL-1β and VEGF-A in HFHCD induced tumor growth is evaluated but it is not fully clear the link between these two molecules rather than that both are mainly produced by myeloid

cells in the tumor.
**Response:** We are grateful for this comment. We had shown that IL-1β controls the level of monocytes in circulation and MDSC accumulation in tumors (Fig. 5c and Supplementary Fig. 4a) but we do agree that we had not clearly addressed its role in tumors. We have performed several new experiments to address this important question.

1- We show, by flow cytometry analysis on tumors, that among immune cells, pro-IL-1β is mainly expressed by myeloid cells (Supplementary Fig. 4c)
2- In the tumor, we found that MDSCs produced similar levels of IL-1β in both groups (Supplementary Fig. 4d) despite high immunosuppressive activities in HFHCD group
3- IL-1β deficiency did not alter immunosuppressive activities of MDSCs (Supplementary Fig. 4b)
4- Specific conditional modulation of IL-1β signaling in myeloid cells by generating LysMCre$^{+/-}$/IL-1R1$^{fl/fl}$ did not alter tumor expansion in response to a HFHCD (Supplementary Fig. 4d)
5- *In vitro* stimulation of bone marrow-derived macrophages and B16-F10 cells with VEGF-A or IL-1β did not influence the production of IL-1β and VEGF-A respectively (Supplementary Fig. 6c, d).

Altogether, these data show that pro-tumoral roles of IL-1β and VEGF-A are different. IL-1β controls monocyte production whereas VEGF-A shapes the tumor microenvironment. IL-1β and VEGF-A would then play non-redundant pro-tumoral activities, which would be spatiotemporally different.

Reviewer #2 (Remarks to the Author); expert on inflammation and cancer:

In this paper, Tran et al. aim to assess the role of mild dyslipidemia in promoting a systemic inflammatory phenotype, accelerating tumor initiation in solid tumors. For this purpose, a mouse model of melanoma was used to show that short-term (two-week) feeding with a high fat/high cholesterol diet (HFHCD) leads to an IL-1β-dependent increase in circulating inflammatory Ly6Chi monocytes, associated with heightened infiltration of myeloid cells within the tumor and accelerated tumor initiation and outgrowth. In this HFHCD setting, myeloid cells are the primary source of proangiogenic VEGFA in the tumor microenvironment. Monocyte depletion reduced immune cell infiltration as well as tumor growth in mice fed with HFHCD, which was recapitulated by specific inhibition of VEGF-A in myeloid cells and in IL1β constitutive KO mice, suggesting that VEGFA secreted by myeloid cells is an important mediator of tumorigenesis in mice on a HFHCD.
The originality of this paper largely lies in investigating the impact of low-grade inflammation and silent dyslipidemia on tumorigenesis but also on the immune microenvironment, rather than in the context of symptomatic dyslipidemia in obese/metabolic syndrome settings, as has been previously done in different tumors models. However, the present study falls short on thoroughly analyzing the impact of HFHCD-driven mild dyslipidemia on the full immune landscape, and the relevance of this model used to generate low-grade inflammation and subclinical dyslipidemia is poorly defined. Moreover, the lack of mechanistic insights into how mild dyslipidemia functionally regulates systemic and local inflammation limits the originality of this study, especially in light of the fact that the majority of the findings of this paper pertaining to IL1B and VEGFA roles in tumorigenesis are already known in different tumor types and context.

**Response:** We thank the reviewer for her/his suggestions. Addressing the comments has significantly enhanced the clarity of the manuscript. Below are point-by-point responses to your major points.

General/major points:

**Reviewer:** 1) The authors have not clearly defined the “low-grade inflammation” associated with their mouse model. A thorough analysis of the leukocyte blood content is lacking, as the authors only show a quantification of circulating Ly6Chi and Ly6Clo monocytes (and later mention a neutrophilia in Supplementary figure 3). A comprehensive analysis of all leukocyte populations is needed to characterize “low-grade” systemic inflammation, both in control mice and tumor-bearing mice, to confirm that it differs from inflammation induced by classic high fat diets in mouse models of obesity or metabolic syndromes.

**Response:** We thank the Reviewer for her/his comments. First, we would like to insist on the fact that, in contrast to many other published studies, we investigated the role of a non-obesogenic high fat high cholesterol diet in tumor growth. We now have added a full characterization of the diet compositions and immune changes induced by the high fat high cholesterol diet (HFHCD) and compared it to an obesogenic 60% HFD. We show in Fig. 1a and Supplementary fig.1 a complete analysis of blood leukocyte counts and spleen leukocyte levels and activation in mice under CD, HFHCD and under obese high fat diet (60% HFD). The first paragraph of our result section details these data and confirms that low-grade inflammation induced by HFHCD differs from a classical obese diet. We have also better characterized the phenotype of macrophages and T cell subsets in tumors and spleen of tumor-bearing mice (Fig.2 and Supplementary Fig.2).
Our data clearly show that the 2 diets have different effects on the immune system and point out the importance and novelty of studying how mild-dyslipidemia controls tumor expansion. We have modified the title and the all manuscript to minimize confusions in the diets.

**Reviewer:** 2) On a similar note, the choice of melanoma as a model system is not obvious, and should be justified.

**Response:** Targeting myeloid cells has become attractive in melanoma patients. First, circulating monocytes/myeloid-derived suppressor cells (MDSCs) is associated with higher risk of melanoma progression (PMID 25353097). Second, the recruitment and expansion of MDSCs is a possible mechanism of limited immunotherapy efficacy in melanoma (PMID 27178742, 24357148). Third, recent papaers suggest that targeting MDSCs in melanoma patients may ameliorate the efficacy of immunotherapy (PMID 30121453).
We have now added in the introduction “In melanoma patients, enrichment of circulating monocytes/myeloid-derived suppressor cells (MDSCs) is associated with higher risk of disease progression (PMID 25353097)”. This precedes a new paragraph introducing MDSCs, which was previously missing.

**Reviewer:** 3) To establish the originality of their model of silent dyslipidemia in a non-obese setting, the study should present a comprehensive analysis of the dyslipidemic state (blood cholesterol but also lipid accumulation in tumor tissue) as well as mouse weights, and compare this to classic high fat diet and obesity/metabolic syndrome mouse models (e.g. Song et al Oncotarget 2017, PMID: 28410190). It is not clear how the HFHCD used in this paper is different, neither are the key cell types responding to this diet.

**Response:** As mentioned in the Reviewer’s point 1, we have now brought extended data on mouse inflammatory status under HFHCD and in comparison to mice under obese diet for 2 weeks as well (60% HFD). The composition of diets is shown in Supplemental Table 1. Data are shown in Fig. 1 and Supplementary Fig.1. In Fig.1 we also show cholesterol levels and mouse and spleen weights. In contrast to 60% HFD, HFHCD increases total cholesterol levels but did not influence body weight or spleen weight (Fig. 1 a-c). Leukocyte subsets in blood and spleen are quite different in both groups. Cell activation markers in spleen also differ between the 2 groups. Those data are reported in the first paragraph of our result section and show that the consumption of a HFHCD for 2 weeks leads to a specific low-grade inflammation, which has never been tested in tumorigenesis before.

In the second paragraph of the result section “HFHCD accelerates melanoma tumor development in C57BL/6J mice” we addressed the role of HFHCD on lipid accumulation in tumor tissues and on cell metabolism. We compared fatty acid composition in CD and HFHCD tumors by gas chromatography–flame ionization detection (GC–FID). We found a significant increase in mono-unsaturated fatty acids and a higher omega-6/omega-3 ratio in the HFHCD-derived tumors. We also found that lipids and tumor explant supernatant stimulate B16-F10 proliferation (Fig. 3 e,f). These data are shown in Fig 2. and fatty acid composition in diets is indicated in Supplemental table 2.

**Reviewer:** 4) No proper justification has been given regarding the choice of studying silent dyslipidemia, e.g. do cases of silent dyslipidemia have increased tumor incidence or a shorter survival when diagnosed with cancer? Further correlation should be done using datasets of clinical samples of lean/overweight/obese cancer patients to ascertain the relevance of studying mild dyslipidemia,

especially in melanoma- could this be associated with differences in immune checkpoint blockade response for instance.

**Response:** A growing body of literature identified subgroups of normal weight individuals with negative health issues similar to those observed in obese patients (Iyengar NM et al, JAMA Oncol 2019, PMID 30520976). Those clinical papers raise the alarm bell on the risk of inadequately categorizing normal BMI patients for low risk of cancer and give proper justification to our study. The Me-Can collaborative study found a correlation between serum triglycerides and the risk of several cancers, among which melanoma was listed (Borena W et al, *Cancer Causes Control* 2011, PMID 21140204).

On the other hand, the American Heart Association together with the American College of Cardiology refined in 2018 the need for optimal hypercholesterolemia management of cancer patients (Grundy SM et al, *JACC*, 2019, PMID 30423393). Controlling latent atherosclerosis had become a lower priority in patients with cancers. Nevertheless, improvement in life expectancy in patients with cancer has led to the finding that some patients with early-stage cancer are most likely to die from a cardiovascular disease that from their original neoplasm (Park NJ et al, Plos One, 2017, PMID : 28934233)

Establishing correlation between inflammatory/lipid markers and risk of cancer development is challenging to assess because clinical data of cancer patients rarely contain inflammatory markers and lipid profile. We think that our study brings here the importance of investigating, in lean individuals, the link between inflammatory/lipid circulating markers typically observed in obese patients and the risk of developing cancer.

**Reviewer:** 5) While the authors have quantified the different immune cell populations infiltrating the tumor (Figure 3), additional characterization of the immune microenvironment should be addressed. What is the functional role of these immune cells, e.g. do macrophages display a more immunosuppressive phenotype compared to CD?

**Response:** We have now added in Fig. 2 and Supplementary Fig. 2 a more complete characterization of the immune microenvironment of both groups of mice. In HFHCD group, we found that tumor macrophages displayed a more M2-like phenotype (Fig. 2h) and that tumor CD8 T cells had decreased activation markers. The decreased anti-tumoral capacity of leukocytes from HFHCD is shown in Fig. 2J. The higher immunosuppressive capacity of MDSCs from the HFHCD group is shown in Supplementary Fig. 4b.

**Reviewer:** 6) The RNAseq data set is a valuable resource of information which is not optimally used in this study (Figure 2). Not only does it contain information regarding immune activation, it also contains information about the activity of metabolic pathways. It will be important to show how the diet effects the metabolism of the tumor cells. Do they rely more on lipid-metabolism? How does this correlate with the corresponding immune response? Linking changing metabolic profile of the tumor in response to the diet to the immune response is needed to provide valuable insights regarding the underlying mechanism of the effect of mild dyslipidemia.

**Response:** We changed the data representation of the transcriptomic analysis by showing the heatmap of the top 100 genes differentially expressed between the CD and HFHCD group with the gene names (Supplementary Fig. 5). We also included in Fig.6a a Volcano plot of all expressed genes, with *Vegfa* and differentially expressed genes from significantly enriched pathway highlighted. Genes differentially expressed in tumor under HFHCD together with fold changes are listed in Supplemental table 3. We found interesting to mention that enriched pathways include "negative regulation of myeloid leukocyte differentiation". This is in line with our new data showing, by luminex analysis, that MDSCs from HFHCD tumors secrete more VEGF-A but not IL-1β and TNF-α (Supplementary Fig. 6a). We specified in the text that "Interestingly, despite the use of tumor bulk transcriptomes, "regulation of myeloid leukocyte differentiation" and "negative regulation of myeloid leukocyte differentiation" pathways were enriched (p= 0.013 and 0.015 respectively), as well as several metabolic pathways …". The text has been modified in consequences (page 9, line 24 and 10).

In order to gain more information on the possible metabolic changes in intra-tumoral leukocytes and tumor cells, we performed new experiments where we monitored glucose and lipid uptakes *in vivo* (Fig.3). We injected specific probes to mice under CD or HFHCD and quantified probe uptake in cells within the tumor by flow cytometry. We found that glucose and lipid combustion differed between cellular subsets *in vivo* in tumors but the pattern was not modulated by the diet. This agrees with our PET-Scan results (Supplementary Fig. 3). Nevertheless, we found that fatty acid composition in tumors differed between the 2 groups. We observed increased proportion of mono-unsaturated fatty acids and a higher omega-6/omega-3 ratio in the HFHCD tumors. We found that B16-F10 cells proliferation rate was increased *in vitro* in response to the mix of fatty acids and in response to tumor explant supernatant. *In vivo*, as mentioned earlier, we found increased levels of Ki67 staining. Altogether, HFHCD does not affect the metabolism of cells in the tumor microenvironment, but rather modulates tumor cell lipogenesis, which probably additionally support tumor cell survival and proliferation.

**Reviewer:** 7) The choice of using IL-1b KO mice to is disputable. Indeed, Il-1b is produced by many immune and non-immune cells and has been broadly involved in a large number of inflammatory responses (Dinarello Mol Med 2014, PMID: 25549233). The authors suggest that it is IL-1b secreted by immune cells that controls the inflammation observed in their HFHCD model, but there is no data showing the potential cellular source of IL-1b; use of a conditional KO model where IL-1b is only suppressed in myeloid cells would be necessary to support that statement. Furthermore, IL-1b is well known to drive many inflammatory responses, including cancer-associated systemic inflammation and infiltration of immune cells in tumor tissues (Wellenstein et al Nature 2019 PMID: 31367040, Incio et al Cancer Discov. 2016, PMID: 27246539, Catstano et al Nat Cell Biol 2018 PMID: 30154549). It was therefore expected that the inflammatory response caused by HFHCD would be impacted by full loss of IL-1b, and further studies to establish a mechanistic link between pro-atherogenic diet and IL-1b-mediated monocytosis would be required to make this finding novel.

**Response:** We agree with the Reviewer comment and we have now added novel data which bring novelty to our findings.
We had shown, in the first version of our manuscript, that IL-1β deficiency impacted myeloid cell number in circulation and avoided Ly6C$^{hi}$ monocytosis in response to HFHCD. These data have been enriched and are shown in Supplementary Fig. 4a. We had also demonstrated that IL-1β deficiency limits the accumulation of MDSCs in tumors of HFHCD-fed mice. We had also found that IL-1β deficiency did not alter the increased immunosuppressive function of MDSCs in HFHCD group (Supplementary Fig. 4b).
First, the Reviewer asked us to identify the cellular source of IL-1β. In the tumor microenvironment, flow cytometry analysis showed that among immune cells, pro-IL-1β was mainly expressed by myeloid cells as no expression was detected in T cells or in CD45- cells (Supplementary Fig. 4c). Lack of IL-1β expression was confirmed in B16-F10 cell line by qPCR. We found that the expression levels of IL-1β in MDSC isolated from tumors of CD and HFHCD-fed mice were similar (Supplementary Fig. 4d).
Second, the reviewer asked us to use a conditional KO model where IL-1β is only suppressed in myeloid cells. We were not able to find IL-1β $^{fl/fl}$ mice. However, we found IL-1R1$^{fl/fl}$ mice and crossed them with to LysMCre mice. When put on HFHCD, IL-1R1$^{\Delta LysM}$ mice increased tumor size whereas they decreased their level of pro-IL-1 β in tumor myeloid cells (Supplementary Fig. 4e). Our data show that modulated production of IL-1 β by myeloid cells does not alter the protumoral effect of HFHCD.

**Reviewer:** 8) The authors chose to use clodronate liposomes to deplete myeloid cells, which has a few caveats including lack of specificity. The use of transgenic mouse models of inducible monocyte/macrophage depletion (Kanter et al Proc Natl Acad Sci U S A. 2012 PMID: 22308341, Goren Am J Pathol 2009 PMID: 19528348) or specific targeting of Ly6Chi monocytes by antibody-mediated depletion (Mack et al J Immunol 2001 PMID: 11254730) would be more appropriate to answer a specific question about the role of increased Ly6Chi monocytes in tumor growth acceleration. Also, the specific depletion of Ly6C low monocytes using this technic is surprising in light of these mentioned studies, the authors should elaborate on these results.

**Response:** We agree that clodronate liposome depletion is not a very elegant way to address the role of myeloid cells in tumor development. We fully accept and learn from this criticism. We then decided to perform more elegant experiments and to remove the clodronate liposome depletion from the manuscript. Instead, we showed the direct implication of myeloid cells in tumor exacerbation under HFHCD by blocking myeloid cell accumulation through various approaches. We treated mice with an

anti-CD115 antibody or with pharmacologic inhibitory agents. Both approaches limited tumor growth in the HFHCD. Similar conclusions were found in Il-1$\beta^{-/-}$ mice, where monocyte expansion is abrogated under HFHCD. Those data are shown in Fig.5.

**Reviewer:** 9) The molecular mechanisms by which mild dyslipidemia affects monocytosis is not addressed in this study- is this related to systemic regulation of monocytic precursors' differentiation or does this regulation take place in the bone marrow? These are important aspects that should be experimentally addressed in this study.

**Response:** We analyzed the composition of myeloid progenitor cells in the 2 groups of mice. We have now added results showing that Common Myeloid Progenitors and Ly6C$^{hi}$ monocytes were increased in the bone marrow of mice under HFHCD (Fig.7). We obtained similar results in mice with (Fig. 7a) or without tumors.
Data on mice without tumors have not been included in the manuscript

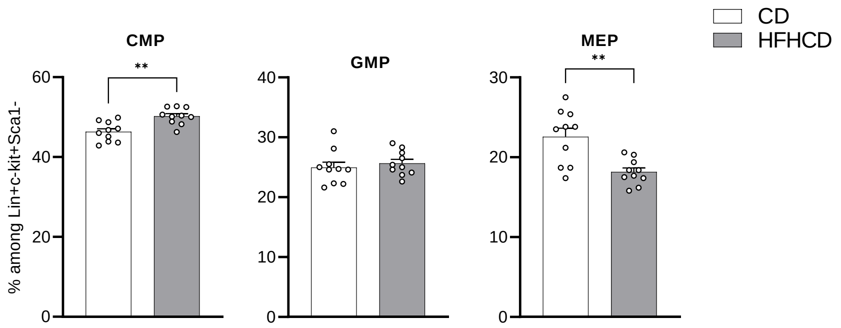


Interestingly, interruption of the HFHCD reduced Ly6C$^{hi}$ monocytes production in the bone marrow, showing that normalization of lipids and cholesterol levels regulate monocyte levels.

**Reviewer:** 10) Little to no analyses of spatial distribution of immune cells in these tumors is presented- this should be included to determine whether niche effects occur related to hypoxia, vascularization etc..in the context of HFHCD.

**Response:** We had previously tested immunohistochemistry staining on frozen section of tumors. We found that the tumor sections were of very poor quality, and decided not to show those stainings. Murine melanoma sectioning is very challenging and we found very few publications with nice pictures of B16-F10 tumor stainings. We then decided to repeat our experiment and paraffin-embedded tumor for sectioning. We found that markers of vascularization (CD31, αSMA) and Ki67 stainings were increased shown in Fig.3 and Fig.7. Quantifications were made under the supervision of an anatomo-pathologist (Dr Brabencova, now added as a co-author).

**Reviewer:** Specific comments:

11) Fig 1A: is it a percentage of CD45+ cells? Or total leukocytes?
**Response:** We have modified the graphs. Now in Fig.1 are represented blood cell counts.

**Reviewer:** 12) Fig 1E: this data is somewhat unconvincing, it does not show a correlation
**Response:** We have removed this figure. We have provided a better characterization of the model in fig.1.

**Reviewer:** 13) Fig 1D: PET-scan is not an appropriate method to measure tumor metabolism in this context, as this will mostly reflect tumor size. Further metabolic analyses should be performed.

**Response:** In order to address the reviewer comment, we performed new experiments where we monitored glucose and lipid uptakes *in vivo* (Fig.3). We injected specific probes to mice under CD or HFHCD and quantified probe uptake in cells within the tumor by flow cytometry. We found that glucose and lipid combustion differed between cellular subsets *in vivo* in tumors but the pattern was not modulated by the diet. This agrees with our PET-Scan results (Supplementary Fig. 3). Nevertheless,

we found that fatty acid composition in tumors differed between the 2 groups. We observed increased proportion of mono-unsaturated fatty acids and a higher omega-6/omega-3 ratio in the HFHCD tumors. We found that B16-F10 cells proliferation rate was increased *in vitro* in response to the mix of fatty acids and in response to tumor explant supernatant. *In vivo*, we found increased levels of Ki67 staining. Altogether, HFHCD does not affect the metabolism of cells in the tumor microenvironment, but rather modulates tumor cell lipogenesis, which probably additionally support tumor cell survival and proliferation.

**Reviewer:** 14) Supp fig 2A: with this method, could they also label monocytes directly in the tumor and not just circulating monocytes?

**Response:** Now on Fig.4b. We agree that we cannot exclude possible staining of already accumulated myeloid cells in the tumor. We did not use this methodology to quantify myeloid cell recruitment in tumors but to qualitatively appreciate that MDSC subsets in the tumors are phenotypically close to monocytes and neutrophils.

**Reviewer:** 15) Line 382: the authors conclude on increased monocyte infiltration of the tumor in HFHCD mice, however this is not clearly supported by the data shown in Supp fig 2B (small number of biological replicates, difference not statistically significant). This experiment should be repeated to strengthen the conclusion.

**Response:** We have now removed this figure and performed adoptive transfer of CD45.1 Ly6Chi monocytes to CD45.2 recipient mice (Fig.4 c, d). With this technique, we were able to quantitatively address the recruitment of Ly6Chi monocytes isolated from CD or HFHCD mice into tumors of CD or HFHCD-fed mice. The results are reported in paragraph "Circulating Ly6Chi monocytes accumulate more in tumors under HFHCD".

**Reviewer:** 16) Fig 3A: this graph represents a count of CD45+ cells, however the increase observed could simply be due to a larger tumor size. This data needs to be normalized to tumor size (e.g. # CD45+ cells per 100g tumor)
**Response:** We have now represented percentage of leukocyte subsets in tumors.

**Reviewer:** 17) Fig 3D: it seems like the figure legend is inaccurate. The colors on the tSNE plot represent different cell populations, not different expression levels.

**Response:** We apologize for this inaccuracy. We have now modified the legend.

**Reviewer:** 18) Fig 4: clodronate liposome injections are not described in the method section, nor referenced

**Response:** We have removed the clodronate liposome experiments and added new experiments using CD115 antibody and chemokine antagonists.

**Reviewer:** 19) Fig 4B and C: why did the authors perform a Mann-Whitney test instead of ANOVA?
**Response:** Those data have been removed and replaced by other experiments (Fig 5)

**Reviewer:** 20) Fig 4D: matching data from the control CD group should be represented in this figure
**Response:** We have removed the clodronate liposome experiments

**Reviewer:** 21) Fig 5: why have the authors performed these assays on the whole CD45+ population? It would be interesting to see this data for more specific subsets, particularly on LyChi monocytes.

**Response:** There was a mistake in the first version of the manuscript, we apologize about that. Luminex was performed in total tumors and not in tumor $CD45^+$ cells. Those data are not correctly identified in Fig. 4a. Due to technical limitation, we were not able to specifically isolate $Ly6C^{hi}$ cells for Luminex experiments. Nevertheless, we have now provided new Luminex data on the supernatant of MDSCs (which contain $Ly6C^{hi}$ cells) isolated from tumors of the 2 groups (Fig. 6b and Supplementary Fig. 6a). Interestingly, we found that MDSCs from HFHCD group produce higher amount of VEGF-A than MDSCs from CD group, but they do not produce more inflammatory cytokines.

**Reviewer:** 22) Fig 5A: are there any differences between CD and HFHCD without LPS and IFNgamma stimulation (basal levels)? Why use splenocytes and not monocyte-derived cells for example?

**Response:** We have removed this experiment from the second version of that manuscript. The use of splenocytes was not convincing and we think that removing it clarifies the message.

**Reviewer:** 23) Fig 5B: There is a large difference in the number of data points for IL-1b KO compared to WT mice, which might cause the difference to be not significant for IL1b KO but significant for WT. A repeat experiment would be needed to have comparable group sizes.

**Response:** Unfortunately we were not able to repeat this experiment as we had to decrease our mouse colony during the first COVID containment. Instead we have been able to use other strategies to confirm that impaired accumulation of myeloid cells in tumors limited tumor expansion under HFHCD (Fig. 5).

**Reviewer:** 24) Fig 6C: Il-1b is not just dispensable for MSDC activity; it looks like there is a higher suppression in Il-1b KO mice, suggesting that Il-1b reduces MSDC immunosuppressive activity.

**Response:** We agree with the reviewer comment, it is true that IL-1β deficiency led to more efficient immunosuppression of T cell proliferation. We have no clear explanation for that. Our new experiments suggest that MDSCs from HFHCD tumors do not rely on IL-1β to accelerate tumor growth (Supplementary Fig. 4d,e and Supplementary Fig. 6a). We think that the HFHCD tumor microenvironment orientates accumulated MDSCs toward an exacerbated angiogenic phenotype, at the expense of a stable inflammatory phenotype. Disruption of IL-1β signaling in LysM$^{cre+/-}$ IL-1R1$^{fl/fl}$ mice did not alter exacerbation of tumor growth under HFHCD. It is possible that IL-1β deficiency increase the immunosuppressive function of MDCSs. We think that experimentally addressing this point is out of the scope of our study.

**Reviewer:** 25) Fig 5D: it would be interesting to see the matching data for mice on control diet.
**Response:** The Reviewer question was to add leukocyte subset proportions in Il-1β$^{-/-}$ mice under CD (Fig 6d in the first version). We have now added these data in Supplementary Fig. 4a.

**Reviewer:** 26) Fig 7D: it is not specified whether the data shown comes from mice on CD or HFHCD
**Response:** Now in Fig. 6e, we have corrected the legend: "Correlation of VEGF-A concentration in tumor with tumor weight, in VEGF-A$^{\Delta\ LysM}$ mice and control littermates, under CD and HFHCD"

**Reviewer:** 27) Supp fig 4c and d: do the authors have an explanation for the decrease in %CD11b+ in blood and %MDSC in tumor in HFD mice compared to CD, when previous figures show increased number of CD11b+ cells in blood and accumulation of MSDC in tumor? Is it a % of CD45+ cells that is shown in this supplementary figure? In supp fig 4c, is there also no change if we look more specifically at 11b+ Ly6Chi subset in blood?

**Response:** We do not have explanation for this observation but the low number of animals and intragroup heterogeneity do not allow us to conclude. We removed these data that are confusing.

**Reviewer:** 28) Fig 7A: The authors should indicate which dots belong to the CD or the HFHCD?
**Response:** We have removed this graph in light of new data presented in the manuscript.

**Reviewer:** 29) Fig 7F: can the authors develop on the meaning of ICAM-1 increase in total tumor?
**Response:** In agreement with previous studies showing that VEGF-A can inhibit ICAM-1 expression (Bouzin C et al, J Immunol 2007, PMID 17237399), we found an increase in ICAM-1 total expression in the tumor in response to HFHCD (Fig. 6g).

Reviewer #3 (Remarks to the Author); expert on angiogenesis:

The link between high-fat diet and cancer progression, has been previously studied. There are several mechanisms have been proposed including the secretion of different fatty acids to promote resistance

of tumor cells and their increased stemness. Immune cells, mesenchymal stem cells and even adipocytes have been demonstrated to affect tumor progression. In this study the authors focused on an in-depth mechanisms of tumor progression in mice fed with high-fat diet involving myeloid derived suppressor cells (MDSCs), which has been previously demonstrated as well. However, the involvement of IL1b and VEGF in this process has not been studied before.
The authors demonstrated that high-fat diet promotes the infiltration of MDSCs to tumors. In turn they secrete VEGFA and IL1b which contribute to tumor progression. The study uses different tools, mostly *in vivo* tools, to evaluate the immune cell composition and the effects of immune cells on tumor growth, cytokine secretion among other parameters.
The reviewer feels that this manuscript does not fit this journal for the following reasons: First, the novelty of the study is questionable, as many additional studies described the basic phenomenon mentioned in this study (Clements 2018 PMID 29345342).

**Response:** We thank the Reviewer for her/his comments. First, we would like to insist on the fact that, in contrast to the paper by Clements VK et al 2018 and to many other published studies, we investigated the role of a non-obesogenic high fat high cholesterol diet in tumor growth. We now have added a full characterization of the diet compositions and immune changes induced by the high fat high cholesterol diet (HFHCD) and compared it to an obesogenic 60% HFD (Fig.1). Our data clearly show that the 2 diets have different effects on the immune system and point out the importance and novelty of studying how mild-dyslipidemia controls tumor expansion. We have modified the title and the all manuscript to minimize confusions in the diets.

**Reviewer:** Second, there are some sophisticated methods that should have been used in order to study the immune composition in the tumor and the involvement of specific MDSC population which promotes tumor growth, e.g., adoptive transfer experiments of myeloid cells from mice fed with high fat diet compared to control mice.

**Response:** We have now performed additional experiments to address the reviewer's concerns. A better characterization of leukocyte phenotype in the tumor is shown in Fig.2 and Supplementary Fig.2. We did adoptive transfer of Ly6Chi monocytes from mice under chow diet or HFHCD to tumor-bearing mice under CD or HFHCD to specifically address the migratory capacities of circulating monocytes (Fig. 4). We also purified MDSCs from CD and HFHCD tumors and analyzed the secretory profile by Luminex and flow cytometry (Fig. 6 and Supplementary Fig. 6).

**Reviewer:** Third, several possible mechanisms involved microbiome, angiogenesis per se, and other direct tumor activities have not been evaluated.

**Response:** We have now addressed the direct role of HFHCD on tumor and immune cell metabolism. We first used the fluorescent D-glucose derivative (2-NBDG) probe to monitor glucose uptake *in vivo*. Then we used fluorescently labelled palmitate (C16 BODIPY) to assess lipid uptake *in vivo*. We analyzed probe uptake by flow cytometry and found that myeloid cells preferentially consumed glucose and lipids, with no difference between the two diets. Next, we compared fatty acid composition in tumors by gas chromatography–flame ionization detection and found a significant increase in proportion of mono-unsaturated fatty acids (MUFAs) and a higher omega-6/omega-3 ratio in the HFHCD-derived tumors. Then, we quantified, *in vitro*, the proliferation rate of tumor cells in response to cholesterol, fatty acids and tumor explant supernatants. All these data are reported in Fig. 3 and in the paragraph "HFHCD accelerates melanoma tumor development in C57BL/6J mice"

We added immunohistochemistry quantification of angiogenic and proliferation markers (Fig. 3 and Fig. 7), which suggests the pro-angiogenic and pro-tumoral effect of HFHCD.

**Reviewer:** Fourth, the use of primary tumors until they reach 200mm3 raises many questions. Why the authors terminate the experiment in this early phase?
**Response:** The experiments were performed until d15, when tumors reached 200mm$^2$ (and not 200mm$^{3)}$.

**Reviewer:** Fifth, the lack of data on metastasis is also surprising especially when studying IL1b and angiogenesis both of which are known to promote metastasis, and the use of B16F10 melanoma promotes pulmonary metastasis.

**Response:** According to the literature, in the B16-F10 model, metastases are observed when cells are injected intravenously. Otherwise, metastasis can be observed after tumor resection (Jung AJ *Carcinogenesis*, 2015; Burghoff S *BMC Cancer* 2014, PMID 25465225; Kubo H *Cancer Immunol Res* 2017 PMID 28811289) In our work, B16-F10 cells were injected subcutaneously and not resected. As now mentioned in the result section, "Within this timeframe, we did not observe any metastasis in the lung nor in the liver".

**Reviewer:** Sixth, some data of IL1b does not fit with previous published studies by Apte's group, one of the major expert in the field. The authors did not find it important to address these discrepancies, e.g., IL1b KO mice in B16 melanoma tumors.
**Response:** Several publications by Apte's group showed the pro-tumoral role of Il-1β (Kaplanov I, PNAS 2019 PMID 30545915). More recently, Apte and collaborators demonstrated the dual role of IL-1β in cancer progression. They showed that blocking IL-1β reduced primary tumor growth but accelerated tumor spread (PMID 25887886, 21300825). Similar contrasted effects were found in the paper by Tulotta C et aln NPJ Breast Cancer, 2021 PMID 34290237). IL-1β effects would depend on specific tumor microenvironment. We do not think that our data do not fit with current knowledge around IL-1β.

**Reviewer:** Seventh, the presentation of the results is no consistent. For example, the number of mice per each immune cell evaluation by flow cytometry raises question (n=4-5 mice per group) when the number of mice in the tumor growth studies was above 10 per group.
**Response:** Within one experiment, mouse tumors were either used for flow cytometry, qPCR, cell sorting or IHC (as not all experiments are feasible on the same tumor), which explain why mouse number is discordant between the techniques.

**Reviewer:** Based on all of these general comments, this reviewer feels that the manuscript is not suitable for publication in this journal.
Below, the reviewer included additional specific comments for the benefit of the authors.
1. I am not sure that the style of the abstract is acceptable. This reviewer suggests that the style of the abstract will be more conservative.
**Response:** We have now modified the abstract

**Reviewer:** 2. OT mice are not defined in methods
**Response:** OT-1 mice are now defined in methods

**Reviewer:** 3. Many abbreviations throughout the text are not defined first time mentioned.
**Response:** We have now corrected this mistake

**Reviewer:** 4. In many places the Greek alphabet letters e.g., beta and gamma are written as b and g. This should be corrected.
**Response:** We have now corrected this mistake

**Reviewer:** 5. As the authors probably aware of, MDSC is a heterogenous population including both monocytic and granulocytic phenotypes with sometimes distinct function in tumors. Can the authors comment on which MDSC populations. This should be define already in the intro and method.
**Response:** We agree with the Reviewer and we have added a paragraph about MDSCs in the introduction.

**Reviewer:** 6. Studies have already demonstrated that MDSCs promote angiogenesis via VEGF. There are studies published a decade ago, showing that VEGF expressed explicitly on myeloid cells, support angiogenesis. This is not a novel part in the study.
**Response:** The novelty is that under a HFHCD (which differ from an obesogenic diet), VEGF-A increase in the tumor is mostly attributable to myeloid source. We used a conditional VEGF-A depletion model to demonstrate that myeloid-derived VEGF-A is responsible for the exacerbation of tumor growth under HFHCD.

**Reviewer:** 7. In the era of microbiome, one would ask whether the effect seen on tumor growth is associated with changes in the gut flor, which then affect the cellular composition of immune cells. While addressing this question is not in the scope of this manuscript, while still can be a mechanism, it should be mentioned at least in the discussion. In addition, an adoptive transfer of MDSC from mice

fed with high-fat diet should be given to mice fed on normal diet in order to evaluate whether the effect on tumor growth disappears. In addition, a reverse experiment of adoptive transfer from mice on normal diet to mice with high-fat diet should change the growth of tumors. These studies are critical to evaluate the impact of MDSC on tumor growth in the context of high fat diet.
**Response:** We performed adoptive transfer with $Ly6C^{hi}$ monocytes to quantify monocyte recruitment to tumors. While we agree that injecting MDSC isolated from tumors of mice on CD or HFHCD would be of great interest, the amount of MDSCs needed (several millions) is not technically feasible due to limitation in cell number. Instead, we blocked MDSC accumulation in the tumor of mice, which revealed the impact of MDSC in the context of HFHCD. We also performed Luminex analysis on MDSCs isolated from tumors on CD or HFHCD.
We found that MDSC are educated in the tumor, which become high producers of VEGF-A but not inflammatory cytokines under HFHCD.
We mentioned the possible role of microbiome in the discussion.

**Reviewer:** 8. The metabolic reprogramming and hypoxia described are not shown in the transcriptomic analysis. how come?
**Response:** We know have provided new graphic presentation of the transcriptomic data (Fig.6 and Supplementary Fig. 5). We have also analyzed tumor and immune cell metabolism (Fig. 3)

**Reviewer:** 9. It is not clear to this reviewer how tumor size of 100-200mm3 weigh 1000mg. For tumors to reach 1000mg they should be in a size of at least 1000-1500mm3. Something is wrong here.
**Response:** Our data mention tumor size in $mm^2$

**Reviewer:** 10. Why the authors did not let the tumor growth much longer to see whether the differences in tumor growth are real? It seems unreasonable to terminate the experiment in 200mm3.
**Response:** Tumor size was measure in $mm^2$ and not $mm^3$

**Reviewer:** 11. .t12. The tSNE plot (figure 3D) is redundant to the results provided in Figure 3BC and can be included in supplemental data.
**Response:** We have modified the figure presentation and hope it does not feel redundant anymore.

**Reviewer:** 13. The B16F10 tumors are metastatic. The fact that the authors have seen an increase in several types of myeloid cells, including monocytic MDSCs suggests that the effect is not only in the primary tumor but also at the metastatic sites. What was the condition of the lungs in mice fed with high fat diet compared to normal diet.
**Response:** As mentioned above, there was no metastasis in our model. We attribute the increase in myeloid cells mainly to the HFHCD and change in lipids and cholesterol distributions.

**Reviewer:** 14. Choldronate liposomes are not specific, and deplete several types of phagocytic cells including some macrophages, alveolar macrophages, neutrophils and others. A better approach should be carried out especially when macrophages and other CD45 monocytic cells enriched in the tumors of high fat diet mice compared with normal diet mice. In addition, the authors did not indicate the mortality rate of the mice administered with chlodronate injections, nor did they mention it in methods.
**Response:** We did not observe mortality after clodronate. We have been using clodronate in many other published studies and never found clodronate to kill mice when properly injected. We agree that clodronate depletion is not specific. We now have removed those data and instead used more elegant strategies to block myeloid cell accumulation in tumors (Fig. 5). We added new experiments using CD115 antibody and chemokine antagonists.

**Reviewer:** 15. The authors focused on the stroma of the tumor to recruit myeloid cells. However, they failed to show whether tumor cells secrete similar factors, therefore indicating that the effect is more general in the tumor microenvironment.
Response: We have performed qPCR and Elisa on B16-F10 cells and found that they produce VEGF-A but not IL-1β (Page 9, line 5).

**Reviewer:** 16. The results with IL1b KO (Figure 6A) do not fit with previously published studies. IL1bKO mice on their own display reduced tumor growth as tested in B16 melanoma. This has not been seen in the authors' data, therefore the reviewer wonder whether IL1b is indeed a factor involved

in the data presented. Thus, the fact that the authors demonstrated reduced tumor growth in IL1b KO mice is misleading and the conclusions do not support the results as well as do not fit with the literature.

**Response:** We found that IL-1β deficiency alters neutrophil levels under chow diet. Melanoma growth was not significantly different in mice IL-1$\beta^{+/+}$ and IL-1$\beta^{-/-}$. We have no clear explanation for that. However, under HFHCD, IL-1β deficiency impaired Ly6C$^{hi}$ monocyte expansion in blood, MDSC accumulation in tumors and tumor development. These data support a pro-tumoral role of IL-1β under HFHCD through increased production of monocytes.

**REVIEWER COMMENTS**

Reviewer #1 (Remarks to the Author):

The authors have certainly listened to the reviewers and have taken their recommendations to heart. The manuscript has been substantially improved. Although, we still do not understand the mechanism through which HFHCD alters the myeloid population in the tumors, the revised manuscript clearly shows that the effect of HFHCD is rapid and reversible. That may be of importance in the treatment of human cancer patients. I do, however, have a few suggestions/comments that need to be addressed prior to final acceptance. This should not require another review cycle.

-The data regarding the effect of HFHCD on B16-F10 and myeloid cell metabolism are not particularly illuminating or interesting. The only meaningful result in the increased Ki67 staining of tumors growing in HFHCD fed mice, but this may belong better in Fig 2 while the remainder of Fig. 3 can be moved to supplementary data.
-IL-1β is a well-established neutrophil growth and survival factor. To determine whether the effect of IL-1β ablation is due to neutrophils or Ly6Chi monocyte expansion the authors should try to selectively deplete both cell populations and determine the effect on tumor growth.
-The effect of CCR2 antagonism mainly correlates with increased tumor size in CD fed mice, rather than decreased tumor size in HFHCD fed mice. Please explain.

Reviewer #2 (Remarks to the Author):

The manuscript has been significantly strenghtened by the additional experiments incorporated by the authors. Particularly, the use of IL1bfl/fl mice was very insightful into showing that myeloid cells are the dominant population secreting IL1b, and the use of anti-CSF1 Ab nicely complement the clodronate liposome approach they previously used.

While the additional analyses on the mechanisms by which mild dyslipidemia affect monocytosis have been touched upon in this revision, it is still unclear what are the molecular players involved in HFHCD increase in CMP production- it would be important to perform cytokine arrays in the blood or BM to determine the factors at the bases of this myeloid progenitor skewing, or whether this is fully dependent on lipids. Invitro differentiation experiments could also be considered.

Author responses to review comments on Nature Communications manuscript NCOMMS-20-39668

**Mild dyslipidemia accelerates tumor growth in mice through expansion of Ly6C$^{hi}$ monocytes and acquisition of a myeloid cell-derived VEGF-A signature.**

Please find hereunder the point-by-point responses

**Responses to Reviewers:**

We would like to thank again the reviewers for their time and effort in reviewing our manuscript. Below is a point-by-point response to your comments.

## REVIEWER COMMENTS

### Reviewer #1 (Remarks to the Author):

The authors have certainly listened to the reviewers and have taken their recommendations to heart. The manuscript has been substantially improved. Although, we still do not understand the mechanism through which HFHCD alters the myeloid population in the tumors, the revised manuscript clearly shows that the effect of HFHCD is rapid and reversible. That may be of importance in the treatment of human cancer patients. I do, however, have a few suggestions/comments that need to be addressed prior to final acceptance. This should not require another review cycle.

**Reviewer:** The data regarding the effect of HFHCD on B16-F10 and myeloid cell metabolism are not particularly illuminating or interesting. The only meaningful result in the increased Ki67 staining of tumors growing in HFHCD fed mice, but this may belong better in Fig 2 while the remainder of Fig. 3 can be moved to supplementary data.

**Response:** We thank the reviewer for his/her suggestions and we have modified the figures and text as recommended.

**Reviewer:** IL-113 is a well-established neutrophil growth and survival factor. To determine whether the effect of IL-113 ablation is due to neutrophils or Ly6Chi monocyte expansion the authors should try to selectively deplete both cell populations and determine the effect on tumor growth.

**Response:** We fully agree that it would be interesting to evaluate whether selective depletion of neutrophils and Ly6C$^{hi}$ monocytes would recapitulate IL-1[illegible] ablation in mice. Previous unpublished experiments performed in our lab were unsatisfying when using anti-GR1 (RB6-8C5) or anti-Ly6G

(1A8) antibodies to deplete monocytes and/or neutrophils. Indeed, we observed transient effects and rebound phases, which eventually led to contradictory results. Repetitive injections of antibodies even led to some toxicity in mice. This lack of efficiency was also reported by others (Moses et al. J. Leukoc. Biol. 2016, (PMID 27252522); Ribechini et al Eur. J. Immunol. 2009, (PMID 19830733) ; Ma et al, J. Leukoc. Biol. 2012, (PMID 23077247)). Recently, Boivin et al, pointed out the limitation of using these antibodies, in part because treated mice rapidly develop anti-antibodies (Boivin G and al. *Nat Com*mun. 2020, (PMID 32488020)). Altogether, these publications show that monocyte and neutrophil depletions remain very difficult to reliably assess, *in vivo*.

-The effect of CCR2 antagonism mainly correlates with increased tumor size in CD fed mice, rather than decreased tumor size in HFHCD fed mice. Please explain.

**Response:** At this point, we can only speculate about potential explanations for this observation. Increased tumor size in mice under CD after CCR2 antagonism might be due to 1- partial depletion of anti-tumoral cytotoxic immune cells and 2- rebound effects in myeloid cell production.

1- CCR2/CCL2 signaling is not specific of monocytes and could also promote the recruitment of cytotoxic immune cells such as NK cells, γδ T cells and T $CD8^+$ cells (Luther et al. *Nat Immunol*, 2001 (PMID 11175801); Fujimura et al. *Sci Rep,* 2015 (PMID 26206182)). Lanca et al. *J Immunol* 2013 (PMID 23686489) have shown that anti-CCL2 neutralization inhibited γδ T cell recruitment in tumor and enhanced tumor growth. We cannot exclude that in our model, inhibiting CCR2/CCL2 also impacted the recruitment of $CCR2^+$ cytotoxic immune cells, thus leading to increased tumor size in CD mice. Indeed, our group and others have shown that there is a cross-talk between cytotoxic $CD8^+$T lymphocytes and macrophages. Thus, the absence of macrophage that precedes the recruitment of $CD8^+$T cells after vaccination inhibits the therapeutic efficacy of anti-tumor vaccines (Thoreau et al. *Oncotarget* 2015 (PMID 26337837)). In addition, $CD8^+$T cells reprogram macrophages towards an anti-tumor phenotype required for tumor shrinkage after cancer vaccine (Van Der Sluis et al. *Cancer Immunol Res* 2015 (PMID 25888578)).

This effect was not observed in mice under HFHCD probably due to higher frequency of monocytes/MDSC in blood, making them more sensitive to inhibition of the CCR2/CCL2 axis than other immune subpopulations.

2- We injected the antagonist once per day to avoid toxicity, which may not have provided a continuous effect. It has been described that interruption of exposure to CCR2/CCL2 can induce a rebound, accelerate monocyte migration and promote tumor growth (Bonaface et al. *Nature,* 2014 (PMID 25337873)).

Reviewer #2 (Remarks to the Author):

The manuscript has been significantly strengthened by the additional experiments incorporated by the authors. Particularly, the use of IL1bfl/fl mice was very insightful into showing that myeloid cells are the dominant population secreting IL1□, and the use of anti-CSF1 Ab nicely complement the clodronate liposome approach they previously used.

**Reviewer:** While the additional analyses on the mechanisms by which mild dyslipidemia affect monocytosis have been touched upon in this revision, it is still unclear what are the molecular players involved in HFHCD increase in CMP production- it would be important to perform cytokine arrays in the blood or BM to determine the factors at the bases of this myeloid progenitor skewing, or whether this is fully dependent on lipids. Invitro differentiation experiments could also be considered.

**Response:** To address the reviewer's comment, we performed a multiplex bead-based immunoassay of growth factors known to favor myeloid progenitors expansion in bone marrow supernatants (obtained as described by Frodermann et al. *Nat Med* 2019 (PMID 31700184)) and in blood plasma of tumor-free mice fed with a CD or with a HFHCD for 2 weeks. GM-CSF, G-CSF, M-CSF, SDF-1/CXCL12 and Leptin were measured. As shown in Supplementary figure 4b (where only detectable cytokines are shown), we found a trend in increased levels of M-CSF and G-SFC and a significant increase in leptin in the bone marrow supernatants of mice under HFHCD, when compared to mice under CD. Leptin is a pro-inflammatory adipokine known to enhance myelopoiesis in mice (Frodermann et al, *Nat Med* 2019 (PMID: 31700184), Trottier et al. *PNAS* 2012 (PMID 22538809). The concurrent increase in G-CSF, M-CSF and leptin in the bone marrow of mice under HFHCD is in line with the increase in CMP production. Flow cytometry and transcriptomic analysis also revealed a higher expression of CXCR4 among HSPC and myeloid progenitors Lin-ckit+Sca1- (assessed only by flow cytometry) (Sup Figure 4c), which most likely ensured the immobilization of progenitor cells in the bone marrow.

We found that CXCL12 tended to decrease in the bone marrow and to increase in the plasma of mice under HFHCD. CXCL12 gradient between bone marrow and peripheral blood was significantly disrupted in mice under HFHCD, which most likely led to the mobilization of Ly6C$^{hi}$ monocytes to the blood compartment observed in this group (Sup Figure 4d). In agreement with this finding, we observed a reduced expression of CXCR4 on mature CD11b+ cells in bone marrow ((Sup Figure 4e), which most likely favored their release into circulation, as previously reported by Chong et al. (*J Exp Med*, 2016 (PMID 27811056))

These results have been added in Sup Fig4 and in the manuscript on page 7 line 21

**REVIEWERS' COMMENTS**

Reviewer #2 (Remarks to the Author):

The authors have addressed the concerns satisfactorily

**Responses to Reviewers:**

We would like to thank again the reviewers for their time and effort in reviewing our manuscript. Below is a point-by-point response to your comment.

## REVIEWER COMMENTS

Reviewer #2 (Remarks to the Author):

The authors have addressed the concerns satisfactorily

**Response:** We thank the reviewer for his comment
